# Synthesis and Characterization of Copper Complexes Featuring a Redox-Active ONO Ligand in Three Molecular Oxidation States

**DOI:** 10.1021/acs.inorgchem.5c01578

**Published:** 2025-05-30

**Authors:** David D. Hebert, Ankita Puri, Daniel Ye, Allison McAninch, Amanda Chisholm, Maxime A. Siegler, Marcel Swart, Isaac Garcia-Bosch

**Affiliations:** † Department of Chemistry, 6612 Carnegie Mellon University, Pittsburgh, Pennsylvania 15213, United States; ‡ 1466 Johns Hopkins University, Baltimore, Maryland 21218, United States; § University of Girona, Campus Montilivi (Ciències), IQCC, Girona 17004, Spain; ∥ ICREA, Pg. Lluís Companys 23, Barcelona 08010, Spain

## Abstract

In this research article, we report the synthesis, characterization, and reactivity of a family of Cu complexes bearing a tridentate iminosemiquinone ligand and ancillary amine ligands ((^sq^ONO)­Cu^II^(L)*_n_*, *n*: 1 or 2). The complexes were obtained following a one-pot synthetic protocol by mixing Cu^II^, 3,5-di-*tert*-butylcatechol, and aqueous ammonia in the presence of an amine base. The Cu complexes were structurally characterized by single-crystal X-ray diffraction analysis (SC-XRD). Cyclic voltammetry measurements showed that the Cu complexes reached three molecular oxidation states in a reversible fashion. The reaction between the Cu-iminosemiquinone complex (^sq^ONO)­Cu^II^(L) with cobaltocene (1e^–^ donor) and ferrocenium (1e^–^ acceptor) produced the corresponding reduced and oxidized complexes. Structural and spectroscopic characterization (SC-XRD, UV–vis, and EPR) of the Cu complexes in the three oxidation states, namely, [(^cat^ONO)­Cu^II^(L)]^−^, (^sq^ONO)­Cu^II^(L), and [(^bq^ONO)­Cu^II^(L)]^+^, suggest that the redox events are ligand-based. DFT computations also formulated the complexes as Cu^II^ species with the ONO ligand in different oxidation states. For the Cu^II^-iminosemiquinone complexes, we calculated small energetic differences between their singlet and triplet states (*S* = 0 vs *S* = 1), which explain their magnetic behavior in solution. Our results provide evidence of how Cu-radical metalloenzymes might tune their electronic structure to modulate their reactivity.

## Graphical abstract

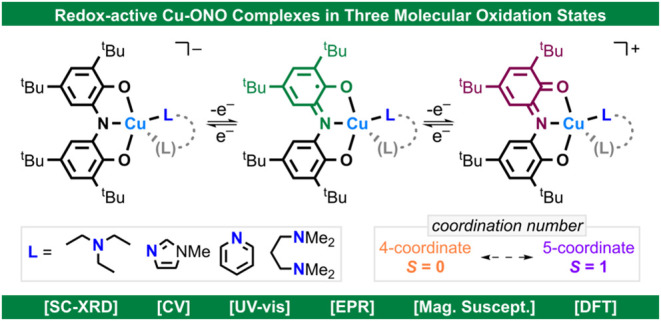

## Introduction

Copper-dependent metalloenzymes, such as galactose oxidase and lytic polysaccharide monooxygenases (LPMOs), leverage cooperativity between copper ions and redox-active amino acid residues (e.g., tyrosine) to carry out oxidative transformations of organic molecules under mild biological conditions. In these enzymes, tyrosine residues (and their corresponding tyrosyl radicals) serve as reservoirs for protons and electrons, facilitating substrate oxidation or protecting the enzyme from deleterious side reactions during uncoupled turnover.^1–4^

Mechanistic studies on the reactivity of galactose oxidase suggest that its reactivity is defined by two states separated by two protons and two electrons: a “protonated” Cu^I^ species that reacts with O_2_ to produce H_2_O_2_ and a “deprotonated” diamagnetic Cu^II^-tyrosyl species that binds and dehydrogenates the alcohol substrate (Figure 1A).^1^ In LPMOs, the role of the tyrosine/tyrosyl redox couple in the catalytic cycle remains a topic of debate. While some studies propose that Cu^II^-tyrosyl intermediates participate in substrate C–H hydroxylation,^5^ recent reports suggest that paramagnetic Cu^II^-tyrosyl species instead function as part of a protective mechanism to prevent autoxidation of the active site in the absence of the substrate (Figure 1B).^2–4^

**Figure 1:**
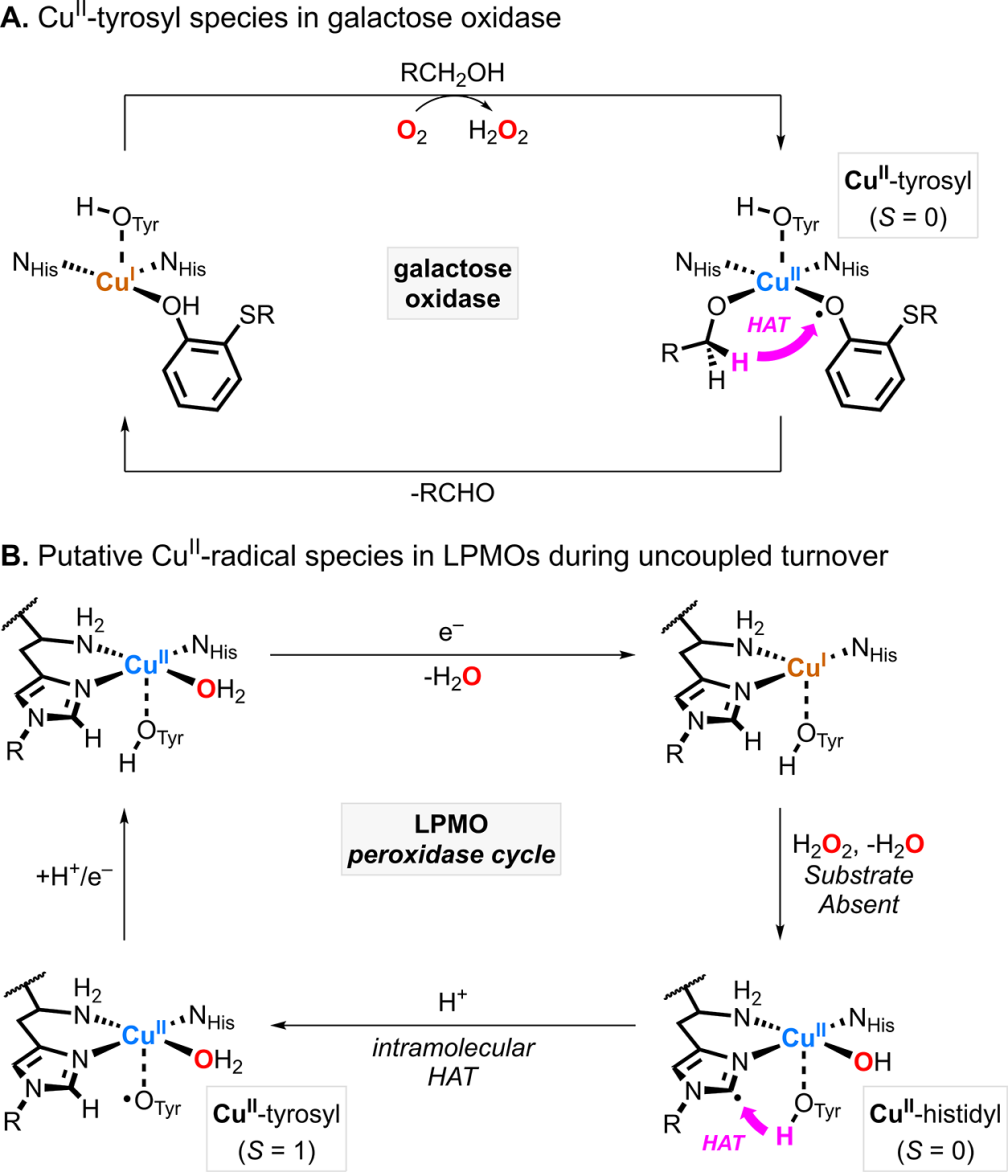
Formation and reactivity of the Cu^II^-radical species in galactose oxidase (A) and lytic polysaccharide monooxygenases (LPMOs) (B).

The discovery of these unique Cu-dependent metalloenzymes has inspired the development of synthetic inorganic complexes designed to mimic the electronic structure and reactivity of Cu-radical intermediates.^6–10^ A key challenge in this field lies in understanding how modifications to the coordination geometry and ligand environment influence the electronic properties and reactivity of Cu complexes bearing redox-active ligands. Such insights are crucial for elucidating the mechanisms and harnessing the chemistry of these metalloenzymes.

The tridentate bis­(3,5-di*tert*-butyl-2-hydroxyphenyl)­amine (ONO) ligand has emerged as a particularly versatile scaffold, capable of forming stable complexes with a diverse range of transition metal^11–22^ and main group^23–26^ ions. This redox-active ligand supports three distinct molecular oxidation states: [^cat^ONO]^3–^ (catecholate-like), [^sq^ONO]^2–^ (iminosemiquinonate), and [^bq^ONO]^−^ (iminobenzoquinonate), each separated by one electron (Scheme 1).

**Scheme 1:**
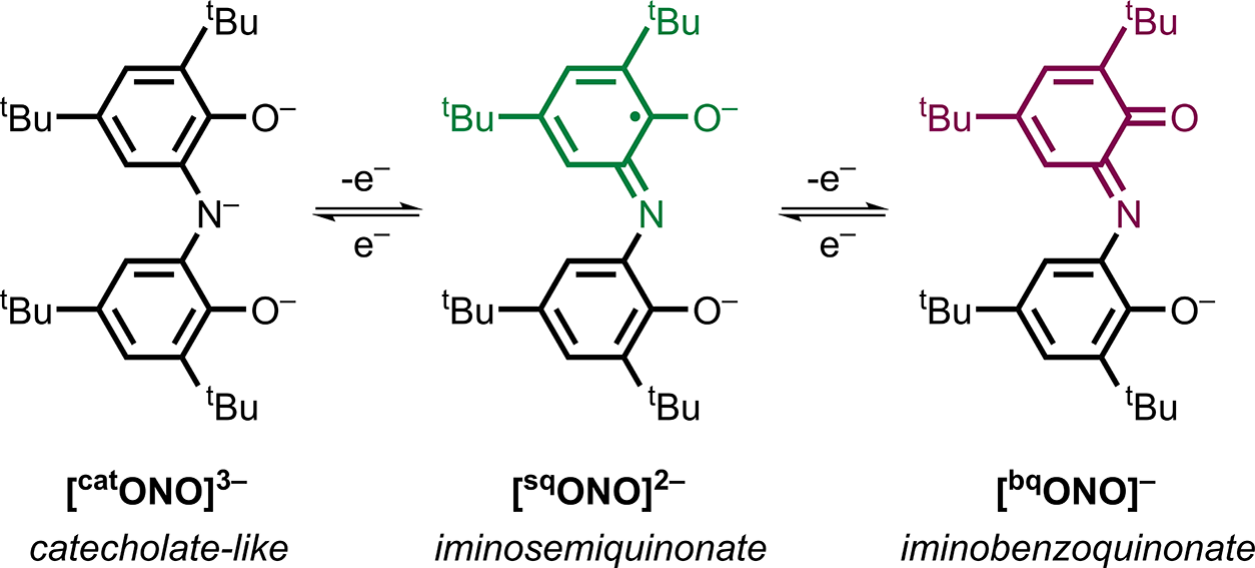
Oxidation States of the ONO Ligand

The ability of the ONO ligand to access multiple oxidation states enables it to act cooperatively with coordinated metal ions to facilitate multielectron redox processes.^20,22,27,28^ For example, the Cu-iminosemiquinone complex (^sq^ONO)­Cu­(NEt_3_) reported by Wieghardt and co-workers^22^ (Figure 2A) was shown to be a highly active catalyst for the aerobic oxidation of alcohols to aldehydes through a process mechanistically similar to galactose oxidase. However, despite their relevance to Cu-dependent metalloenzymes, examples of Cu complexes incorporating the ONO ligand remain scarce (Figure 2A).^11,19,22^ Moreover, existing examples of Cu complexes containing the [^sq^ONO]^2–^ ligand lack adequate spectroscopic and structural characterization.

**Figure 2:**
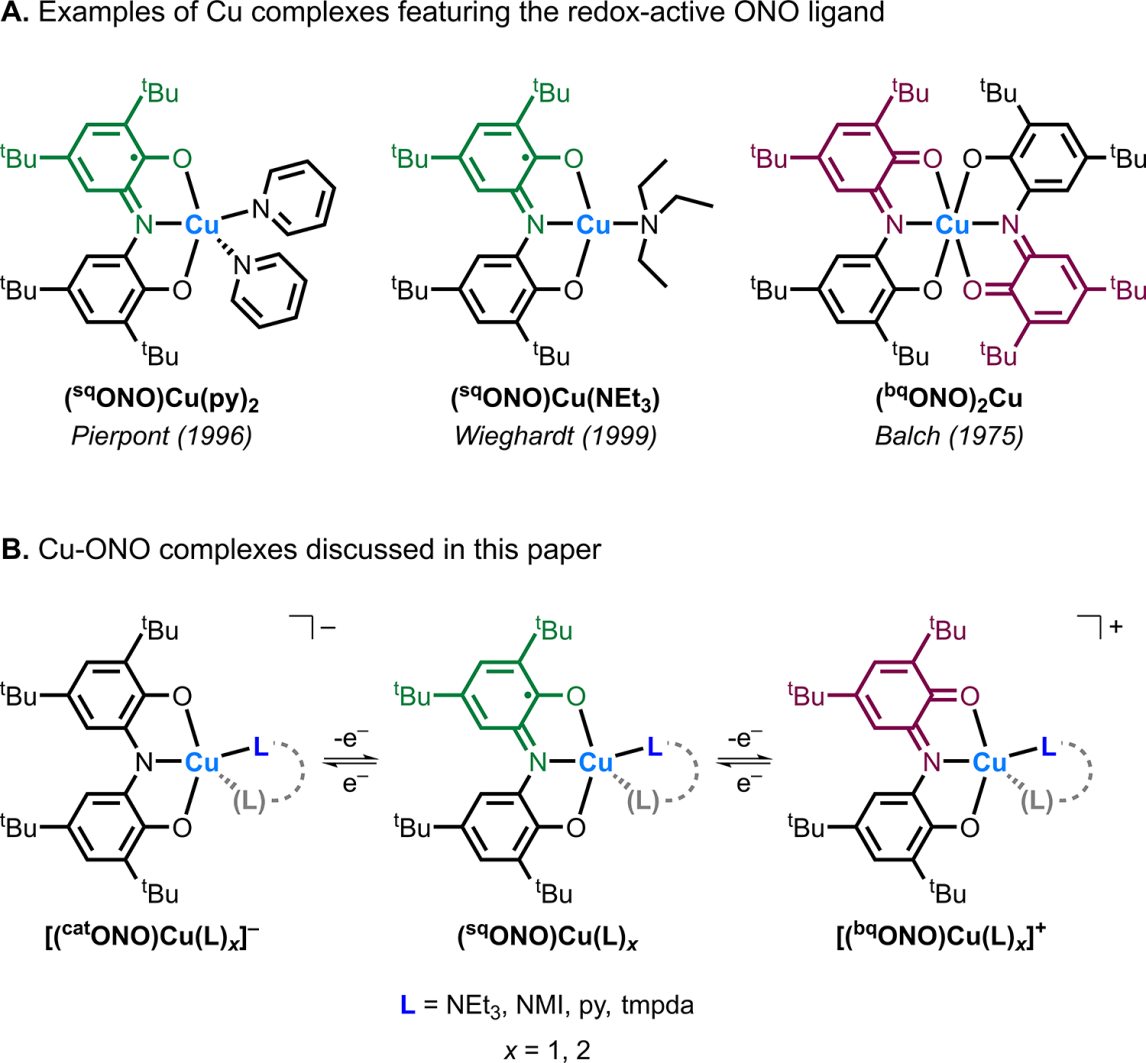
(A) Examples of previously reported Cu complexes featuring the redox-active ONO ligand. (B) The Cu-ONO complexes discussed in this paper include triethylamine (NEt_3_), *N*-methylimidazole (NMI), pyridine (py), and *N*,*N*,*N*′,*N*′-tetramethyl-1,3-propanediamine (tmpda).

To address this, we synthesized a series of Cu-iminosemiquinone complexes with the general form (^sq^ONO)­Cu­(L) to investigate the influence of the ancillary amine ligand (L = NEt_3_, NMI, py, tmpda) on their electronic structures (Figure 2B). The Cu complexes were characterized by single-crystal X-ray diffraction (SC-XRD), cyclic voltammetry, UV–vis spectroscopy, EPR spectroscopy, and magnetic susceptibility measurements (Evans method). Additionally, we structurally and spectroscopically characterized the one-electron oxidized and reduced forms of the complexes and performed density functional theory (DFT) calculations to gain further insight into their spin states and electronic structures.

## Results and Discussion

### Synthesis of the (^sq^ONO)­Cu­(L) Complexes

The (^sq^ONO)­Cu­(L) complexes described in this study were prepared using a modified procedure for the synthesis of (^sq^ONO)­Cu­(py)_2_ reported by Pierpont and co-workers.^19^ In a typical reaction, the desired amine ligand (L), 3,5-di-*tert*-butylcatechol (3,5-DTBC), CuCl_2_·2H_2_O, and aqueous NH_4_OH were combined in acetonitrile and stirred under an aerobic atmosphere (air or O_2_) for 4 h at room temperature (Figure 3). Cooling the reaction mixture to −5 °C and subsequent vacuum filtration afforded the complexes as dark powders. Purification of the Cu-iminosemiquinone complexes was achieved by dissolving the crude products in diethyl ether under an inert atmosphere, followed by filtration and evaporation of the solvent under reduced pressure (Scheme S1A).

**Figure 3:**
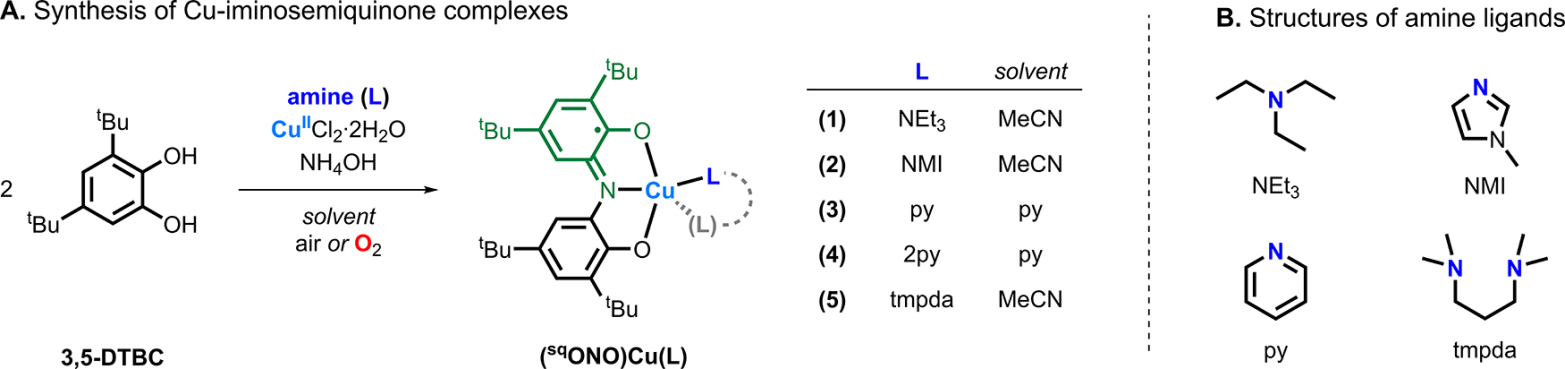
(A) General synthetic scheme and reaction conditions for Cu-iminosemiquinone complexes **1–5**. (B) The structures of the ancillary amine ligands (L) used in the synthesis of the (^sq^ONO)­Cu­(L) complexes.

The pyridine complexes (^sq^ONO)­Cu­(py) and (^sq^ONO)­Cu­(py)_2_ were synthesized using the same procedure described above, substituting pyridine for acetonitrile as both the solvent and the ancillary amine ligand. Notably, both pyridine complexes could be isolated from a single reaction mixture (Scheme S1B). Following filtration of the crude reaction mixture as described above, purification of the resulting solid with diethyl ether yielded the monopyridine complex (^sq^ONO)­Cu­(py). A small crop of dipyridine complex (^sq^ONO)­Cu­(py)_2_ was subsequently recovered from the filtrate after storage at −5 °C for over 48 h.

With our modified synthetic procedure, we demonstrate that this one-pot method can be used to prepare Cu-iminosemiquinone complexes with a variety of alkyl and aromatic amine ligands. Compared to the synthesis of (^sq^ONO)­Cu­(NEt_3_) previously reported by Wieghardt and co-workers,^22^ this approach offers the advantage of forming the [^sq^ONO]^2–^ ligand *in situ*, thereby eliminating the need to prepare the O_2_- and light-sensitive trifluoroacetate (TFA) salt proligand [(^cat^ONO)­H_4_]­[TFA].

The synthesis of (^sq^ONO)­Cu­(py)_2_ originally reported by Pierpont and co-workers was conducted under an O_2_ atmosphere.^19^ To investigate whether the ambient atmosphere could be used in place of O_2_, we performed parallel syntheses of the Cu-iminosemiquinone complexes under both O_2_ and ambient atmospheres. The choice of an aerobic atmosphere was found to have a moderate but notable impact on the yields of the complexes (Table 1). Reactions conducted under an O_2_ atmosphere generally afforded higher yields compared with those performed under air. However, the reduced yield may be an acceptable trade-off to avoid the need for a dedicated O_2_ source (e.g., an oxygen cylinder).

**Table 1:** Isolated Yields (g) of (^sq^ONO)­Cu­(L)_*x*_ Complexes Synthesized under Oxygen and Ambient Atmospheres

complex	L	solvent	x	yield with O_2_ (%)	yield with Air (%)
1	NEt_3_	MeCN	1	0.76 (52%)	0.54 (37%)
2	NMI	MeCN	1	0.80 (57%)	0.43 (31%)
3	py	py	1	0.76 (54%)	0.34 (24%)
4	py	py	2	0.10 (6%)	0.22 (14%)
5	tmpda	MeCN	1	1.00 (65%)	1.10 (72%)

Efforts to synthesize Cu-iminosemiquinone complexes with vicinal diamine ligands, such as 2,2′-bipyridine (bpy) or *N*,*N*,*N*′,*N*′-tetramethylethane-1,2-diamine (tmeda), were unsuccessful, yielding primarily ether-insoluble products. Similarly, ligand exchange reactions of isolated Cu-iminosemiquinone complexes with vicinal diamine ligands, for example, (^sq^ONO)­Cu­(py) + bpy → (^sq^ONO)­Cu­(bpy) + py, were also unproductive.

### Characterization of the Cu Complexes by SC-XRD

The SC-XRD structures for the (^sq^ONO)­Cu­(L)_*x*_ complexes **1**–**5** are shown in Figure 4 (see further details in the Supporting Information). Single crystals of **1**–**3** and **5** were obtained by the slow evaporation of diethyl ether solutions under anaerobic conditions. Crystals of **4** were obtained after separation of **3** from the crude reaction mixture by filtration and storage of the resulting filtrate at −5 °C for over 48 h (Scheme S1B). We note that while complex **4** was previously reported by Pierpont and co-workers,^19^ it had not been characterized by SC-XRD.

**Figure 4:**
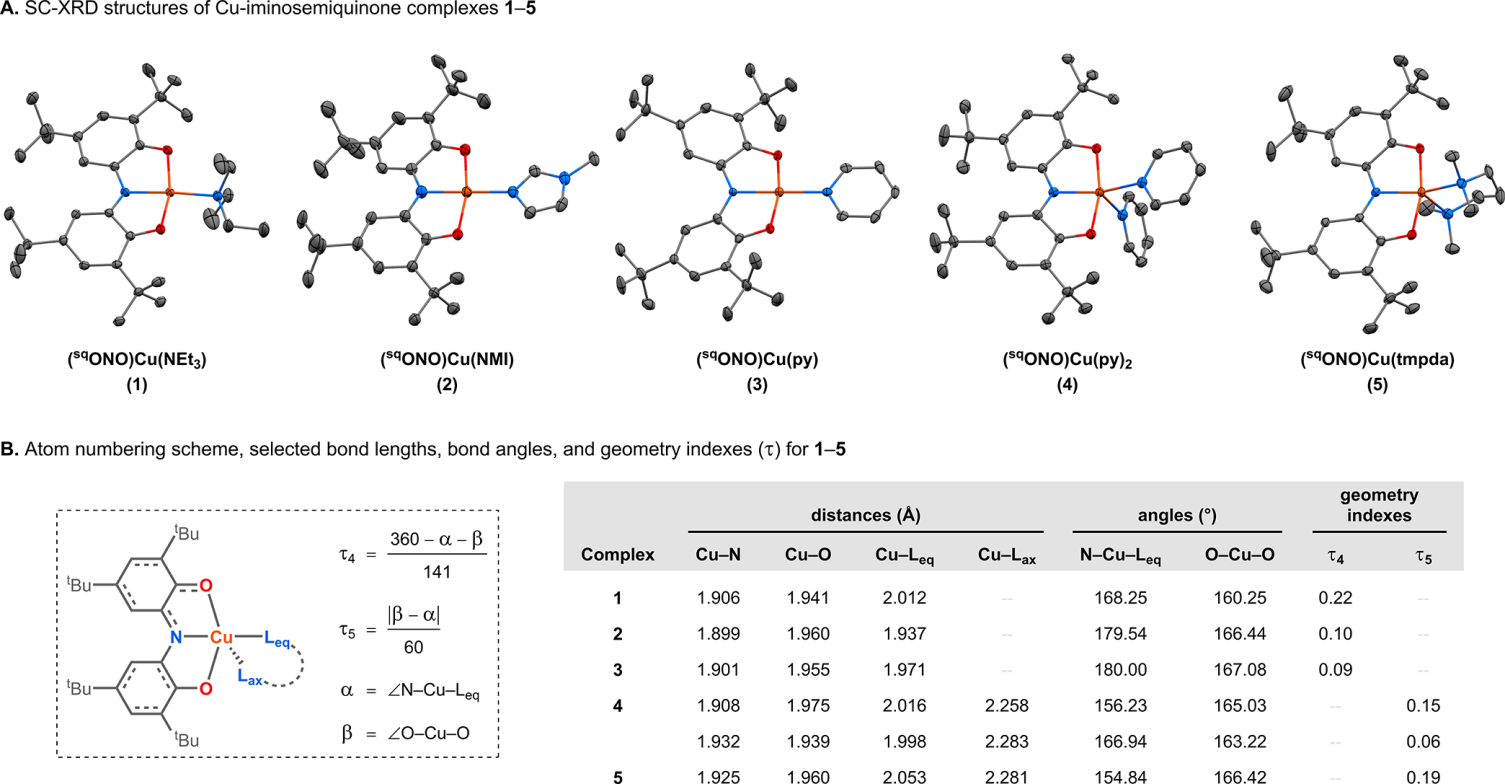
(A) SC-XRD structures of (^sq^ONO)­Cu­(L)_*x*_ complexes **1–5**. Thermal ellipsoids are shown at a 50% probability level. Disorder, hydrogen atoms, and lattice solvent molecules are omitted for clarity. (B) The atom numbering scheme and selected bond lengths, bond angles, and geometry indices^29^ (τ_4_: 0 = square planar, 1 = tetrahedral; τ_5_: 0 = square pyramidal, 1 = trigonal bipyramidal) for **1–5**. Cu–O bond lengths represent the average of both Cu–O bonds. Structural parameters are provided for both Cu complexes present in the unit cell of **4**. See further details (including refined bond distances and angles with standard uncertainties) in the Supporting Information.

Crystallographic analysis revealed that complexes **1**–**3** adopt square-planar geometries. Complexes **2** and **3** are highly square-planar (τ_4_ ≤ 0.1), whereas **1** shows moderate tetrahedral distortion (τ_4_ = 0.22), due to the slight displacement of the NEt_3_ ligand out of the (ONO)Cu plane (∠N–Cu–L_eq_ = 168.25°; Figure 4). The five-coordinate complexes **4** and **5** adopt slightly distorted square-pyramidal geometries (τ_5_ = 0.06–0.19; Figure 4). Two crystallographically independent structures are present in the unit cell of complex **4** (Figure S4).

Across the series of complexes, only minor variations in the Cu-L_eq_ bond distances were observed (ΔCu–L_eq_ = 0.116 Å). Among complexes with the same coordination number, aromatic amine ligands (NMI, py) yielded Cu-L_eq_ bonds shorter than those of aliphatic amine ligands (NEt_3_, tmpda). Differences in the Cu–N and Cu–O bond lengths to the ONO ligand were comparatively small (ΔCu–N = 0.033 Å; ΔCu–O = 0.036 Å), with shorter bonds generally observed in the four-coordinate complexes.

### Electrochemistry

Cyclic voltammogram measurements were performed for each complex (1 mM) in N,N-dimethylformamide (DMF) with [NBu_4_]­PF_6_ (0.1 M) as the supporting electrolyte (Figure 5). All complexes exhibited two reversible one-electron processes at −0.40 and −1.08 V versus Fc^+/0^ (Table 2). These redox events are attributed to ligand-centered processes and are assigned to the [(^bq^ONO)­Cu­(L)]^+^/(^sq^ONO)­Cu­(L) and (^sq^ONO)­Cu­(L)/[(^cat^ONO)­Cu­(L)]^−^ couples, respectively (see further discussion in the following sections).

**Figure 5:**
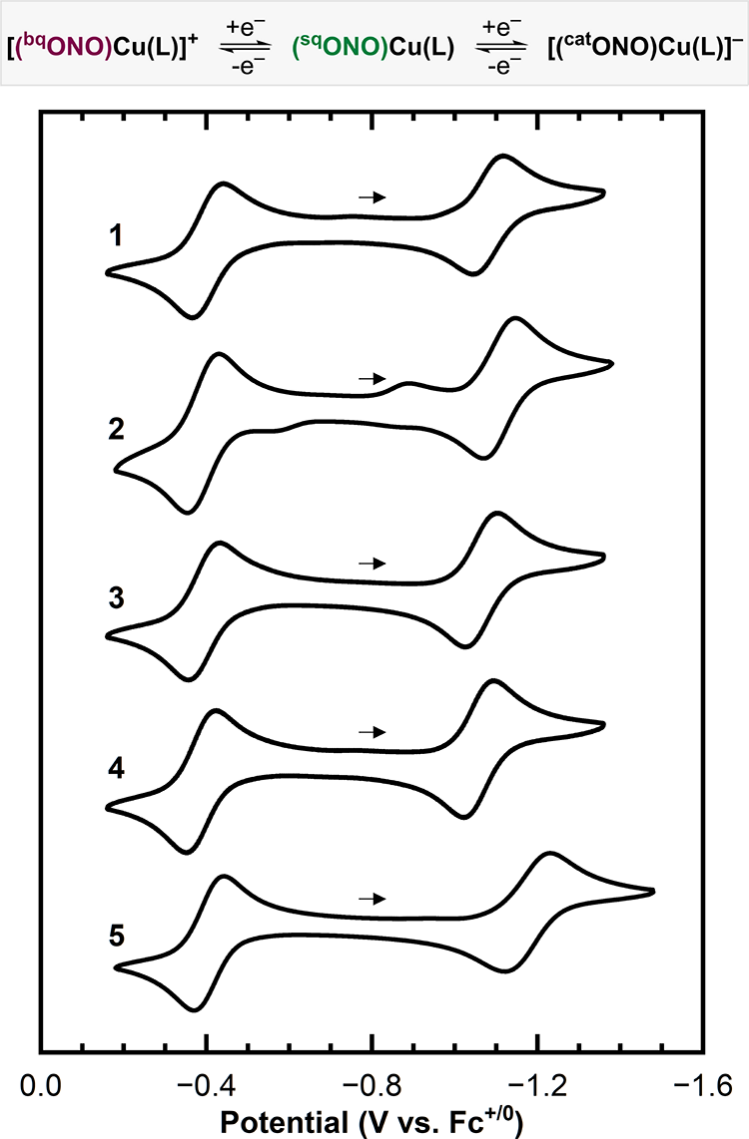
Cyclic voltammograms of the (^sq^ONO)­Cu­(L)_*x*_ complexes collected in DMF at room temperature under argon with 0.1 M [NBu_4_]­PF_6_ as the supporting electrolyte.

**Table 2:** Cyclic Voltammetry Redox Potentials (V) and Peak-to-Peak Separations (Δ*E*, mV) of (^sq^ONO)­Cu­(L) Complexes in DMF Relative to the Fc^+/0^ Redox Couple

complex^a^	L	*E*_1/2_ V vs Fc^+/0^ (Δ*E*, mV)^b^	
1	NEt_3_	–0.40 (75)	–1.08 (75)
2	NMI	–0.39 (72)	–1.11 (76)
3	py	–0.40 (76)	–1.06 (75)
4	2py	–0.39 (72)	–1.06 (73)
5	tmpda	–0.41 (68)	–1.18 (108)

a 1 mM complex in DMF with the 0.1 M [NBu_4_]­PF_6_ supporting electrolyte.

b Scan rate = 0.1 V/s.

The identity of the ancillary amine ligand had a modest influence on the redox potentials, particularly the [(^bq^ONO)­Cu­(L)]^+^/(^sq^ONO)­Cu­(L) couples, which all occurred within a narrow range of 20 mV (−0.39 to −0.41 V). The (^sq^ONO)­Cu­(L)/[(^cat^ONO)­Cu­(L)]^−^ couples showed slightly greater variability, spanning 50 mV (−1.06 to −1.11 V) for complexes **1**–**4** with monodentate ancillary amine ligands. However, the (^sq^ONO)­Cu­(L)/[(^cat^ONO)­Cu­(L)]^−^ couple for **5** is shifted cathodically (to more negative potentials) to −1.18 V and is quasi-reversible (Δ*E* = 108 mV). This cathodic shift likely results from the stronger electron-donating ability of the bidentate tmpda ligand, and the quasireversibility suggests a possible change in its binding mode (bidentate or monodentate). We note that the similarity in redox potentials between the two pyridine complexes **3** and **4** is likely due to the dissociation of the apical pyridine ligand of **4** in solution in the absence of excess pyridine.^27^

### Synthesis and Characterization of the One-Electron Oxidized and Reduced Forms of **5**

The one-electron-oxidized (**5-bq**) and reduced (**5-cat**) forms of complex **5** were synthesized by treatment of an acetonitrile/toluene solution of **5** with ferrocenium hexafluorophosphate (FcPF_6_) or cobaltocene (CoCp_2_), respectively, under anaerobic conditions (Figure 6). Workup and isolation of the complexes afforded **5-bq** as a green powder and **5-cat** as a dark purple powder. Single crystals of **5-bq** suitable for SC-XRD were obtained by layering hexane on a toluene solution, and single crystals of **5-cat** were obtained by layering diethyl ether on an acetonitrile/toluene solution at –35 °C.

**Figure 6:**
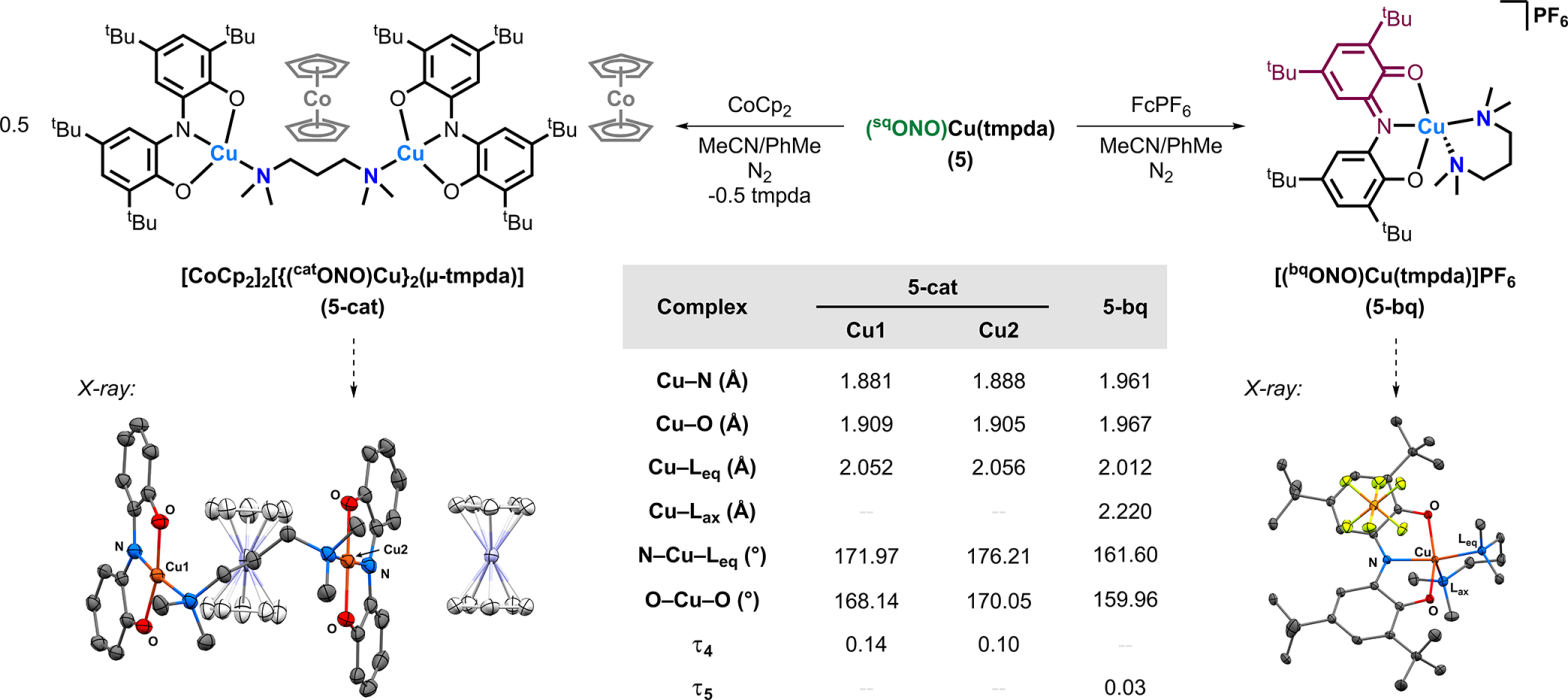
Scheme for the synthesis of the one-electron reduced (**5-cat**) and oxidized (**5-bq**) forms of **5** (top). SC-XRD structures of **5-cat** and **5-bq**, along with selected bond distances, bond angles, and geometry indices, are shown (bottom). Cu–O bond lengths represent the average of both Cu–O bonds. Hydrogen atoms are omitted from both structures for clarity, as well as the ONO ligand *tert*-butyl groups and lattice solvent molecules of **5-cat**. Thermal ellipsoids are shown at a 50% probability level. See further details (including refined bond distances and angles with standard uncertainties) in the Supporting Information.

X-ray diffraction experiments revealed that the reduction of **5** with CoCp_2_ led to the dimerization of two Cu centers with the loss of one tmpda ligand (Figure 6; note: CoCp_2_^+/0^ *E*_1/2_ = −1.29 V vs Fc^+/0^ in DMF).^30^ The remaining tmpda ligand bridges the two Cu centers in a monodentate fashion, resulting in a dinuclear species formulated as [CoCp_2_]_2_[{(^cat^ONO)­Cu}_2_(μ-tmpda)] (**5-cat**), as confirmed by elemental analysis. Two cobaltocenium [CoCp_2_]^+^ counterions are present in the unit cell, with one cobaltocenium ion sandwiched between the bridged (ONO)Cu fragments. Both Cu centers possess slightly distorted square-planar geometries (τ_4_ = 0.14, 0.10) and exhibit contracted Cu–N (0.044 Å, 0.037 Å) and Cu–O (0.051 Å, 0.055 Å) bonds compared to **5**.

Oxidation of **5** with FcPF_6_ afforded a mononuclear species formulated as [(^bq^ONO)­Cu­(tmpda)]­PF_6_ (**5-bq**), as determined by SC-XRD (Figure 6) and confirmed by elemental analysis. The structure of **5-bq** features a square-pyramidal geometry similar to that of **5** but with reduced trigonal bipyramidal distortion (τ_5_ = 0.03). Only minor changes in Cu–ONO bond distances are observed relative to **5**, including a 0.036 Å elongation of the Cu–N bond. The oxidized complex also exhibits slightly shorter Cu–L_eq_ and Cu–L_ax_ distances, contracted by 0.041 and 0.061 Å, respectively.

### Metrical Oxidation State Analysis

The “metrical oxidation state” (MOS) method outlined by Brown and co-workers^31^ was used to calculate the MOS of the ONO ligand in the SC-XRD structures **1**–**5**, **5-cat**, and **5-bq**. The MOS is a value that correlates the intraligand bond lengths with the apparent oxidation state of the ligand, where a value of −3 corresponds to catecholate-like ([^cat^ONO]^3–^), −2 to iminosemiquinonate ([^sq^ONO]^2–^), and −1 to iminobenzoquinonate ([^bq^ONO]^−^) as shown in Figure 7A. The “typical” intraligand bond distances^31^ for each oxidation state of the ONO ligand are shown in Figure 7B (dashed lines).

**Figure 7:**
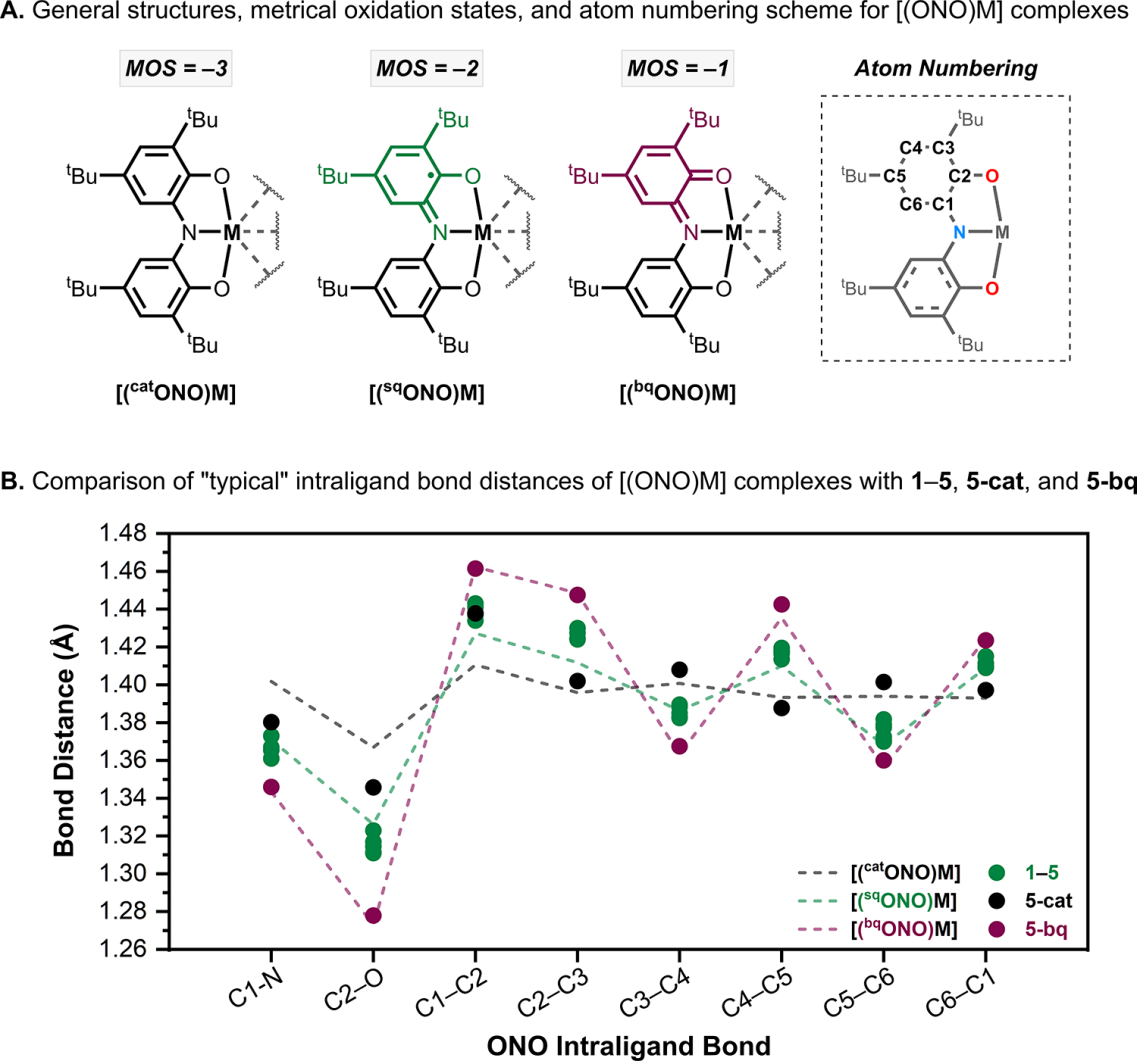
(A) ONO ligand atom numbering scheme, general structures, and metrical oxidation state (MOS) values of [(^cat^ONO)­M], [(^sq^ONO)­M], and [(^bq^ONO)­M] complexes. (B) Comparison of the ONO intraligand bond distances in **1–5** (green ●), **5-cat** (black ●), and **5-bq** (maroon ●) with the “typical” bond distances^31^ of [^cat^ONO]^3–^ (black dashed line), [^sq^ONO]^2–^ (green dashed line), and [^bq^ONO]^−^ (maroon dashed line) ligands in [(ONO)­M] complexes. Note: The intraligand bond lengths of **1–5**, **5-cat**, and **5-bq** are the average of chemically equivalent bonds in the SC-XRD structures.

The ONO ligand displays distinct changes in intraligand bond distances depending on its oxidation state. For example, in the [^cat^ONO]^3–^ state, the intraligand C–C bond distances are nearly equal (Figure 7B, black dashed line), consistent with an aromatic system. In contrast, the one- and two-electron oxidized forms of the ligand exhibit alternating C–C bond lengths (Figure 7, green and maroon dashed lines), reflecting their quinone-like structure. Specifically, the C3–C4 and C5–C6 bonds contract, and the C1–C2, C2–C3, and C4–C5 bonds elongate, corresponding to increased double-bond and single-bond character, respectively. Additionally, the oxidized ligands feature shorter C1–N and C2–O bonds than the [^cat^ONO]^3–^ ligand due to greater double-bond character. These bond length changes are most pronounced in the [^bq^ONO]^−^ state and, to a lesser extent, in the [^sq^ONO]^2–^ state.

The average ONO intraligand bond distances obtained from the SC-XRD structures of **1**–**5**, **5-cat**, and **5-bq** are plotted in Figure 7B (circles). The Cu-iminosemiquinone complexes **1**–**5** (green circles) all display intraligand bond lengths consistent with the “typical” bond lengths of [^sq^ONO]^2–^ ligands (green dashed line) found in other [(^sq^ONO)­M] complexes. This is reflected in the calculated MOS values of the ONO ligands in **1**–**5**, which are all close to −2 (Table 3).

**Table 3:** Average Intraligand Bond Lengths (Å) and Metrical Oxidation State (MOS) of the ONO Ligand in Complexes **1–5**, **5-cat**, and **5-bq** Derived from SC-XRD Structures^a^

complex	C1–N	C2–O	C1–C2	C2–C3	C3–C4	C4–C5	C5–C6	C6–C1	MOS
1	1.366	1.312	1.438	1.428	1.383	1.420	1.378	1.412	–1.8
2	1.361	1.317	1.434	1.425	1.390	1.418	1.370	1.410	–1.9
3	1.373	1.323	1.437	1.424	1.383	1.418	1.379	1.409	–2.0
4	1.366	1.315	1.443	1.425	1.388	1.414	1.382	1.410	–1.9
5	1.367	1.311	1.441	1.430	1.385	1.417	1.373	1.415	–1.8
5-cat	1.380	1.346	1.438	1.402	1.408	1.388	1.402	1.397	–2.6
5-bq	1.346	1.278	1.462	1.448	1.368	1.443	1.360	1.424	–1.0

a The intraligand bond lengths represent the average of chemically equivalent bonds in the SC-XRD structures. See further details (including refined bond distances with standard uncertainties) in the Supporting Information.

Analysis of the intraligand bond lengths of reduced complex **5-cat** (Figure 7, black circles) indicated a MOS value of −2.6 for both (ONO)­Cu fragments (Table 3), consistent with the reduction of the ligand to the [^cat^ONO]^3–^ state. The ONO C–C bonds show minimal bond length alternation; however, the C1–C2 bond is significantly elongated (1.438 Å) relative to the other C–C bonds. Additionally, the C1–N and C2–O bonds in this complex are shorter than those typically found in [(^cat^ONO)­M] complexes (Figure 7, black dashed line). These deviations contribute to the higher-than-expected MOS value obtained for **5-cat** (−2.6 vs −3.0).

The oxidized complex **5-bq** (Figure 7, maroon circles) exhibits intraligand bond lengths which are in close agreement with previously reported [(^bq^ONO)­M] complexes (Figure 7, maroon dashed line). A MOS value of – 1.0 (Table 3) was calculated for the ONO ligand, which is in good agreement with the assignment of **5-bq** as a Cu^II^–iminobenzoquinonate complex.

### UV–Vis Spectroscopy

UV–vis absorption spectra of **1**–**5** were recorded at room temperature in DMF under argon. The spectra exhibit several common features: a sharp, intense band at 393–399 nm (ε = 15400–21000 M^–1^cm^–1^), a weak feature at 510–524 nm (ε = 1570–2010 M^–1^cm^–1^), and a medium-intensity band at 993–1020 nm (ε = 4960–9510 M^–1^cm^–1^) (Table S15). The absorption features of **1**–**5** are consistent with previously reported metal complexes featuring the [^sq^ONO]^2–^ ligand,^18,25,32^ and support the assignment of the complexes as (^sq^ONO)­Cu^II^(L) species rather than (^bq^ONO)­Cu^I^(L) or (^cat^ONO)­Cu^III^(L) valence tautomers (see further discussion below).

Chemical reduction and oxidation of the Cu-iminosemiquinone complexes produced distinct spectral changes consistent with ligand-centered redox processes (Figures 8 and S8–S10). For example, treatment of **1** with one equivalent of CoCp_2_ resulted in quenching of the absorption features associated with the [^sq^ONO]^2–^ ligand above 400 nm (Figure 8A). This loss of absorption features suggests the reduction of the ONO ligand to the catecholate-like state, as other metal complexes bearing the [^cat^ONO]^3–^ ligand^16^ or the catecholate-like forms of analogous NNN^33–37^ and SNS^38^ pincer ligands similarly lack significant absorptions above 400 nm. On the basis of these spectral changes, we assign the reduced species **1-cat** as a Cu^II^-catecholate complex. The same assignment is given to the reduced forms of **2**–**5** (e.g., **2-cat**, **3-cat**, etc.), as the reduction of these complexes with CoCp_2_ yielded spectra nearly identical to **1-cat** (Figures 8D and S8–S10).

**Figure 8:**
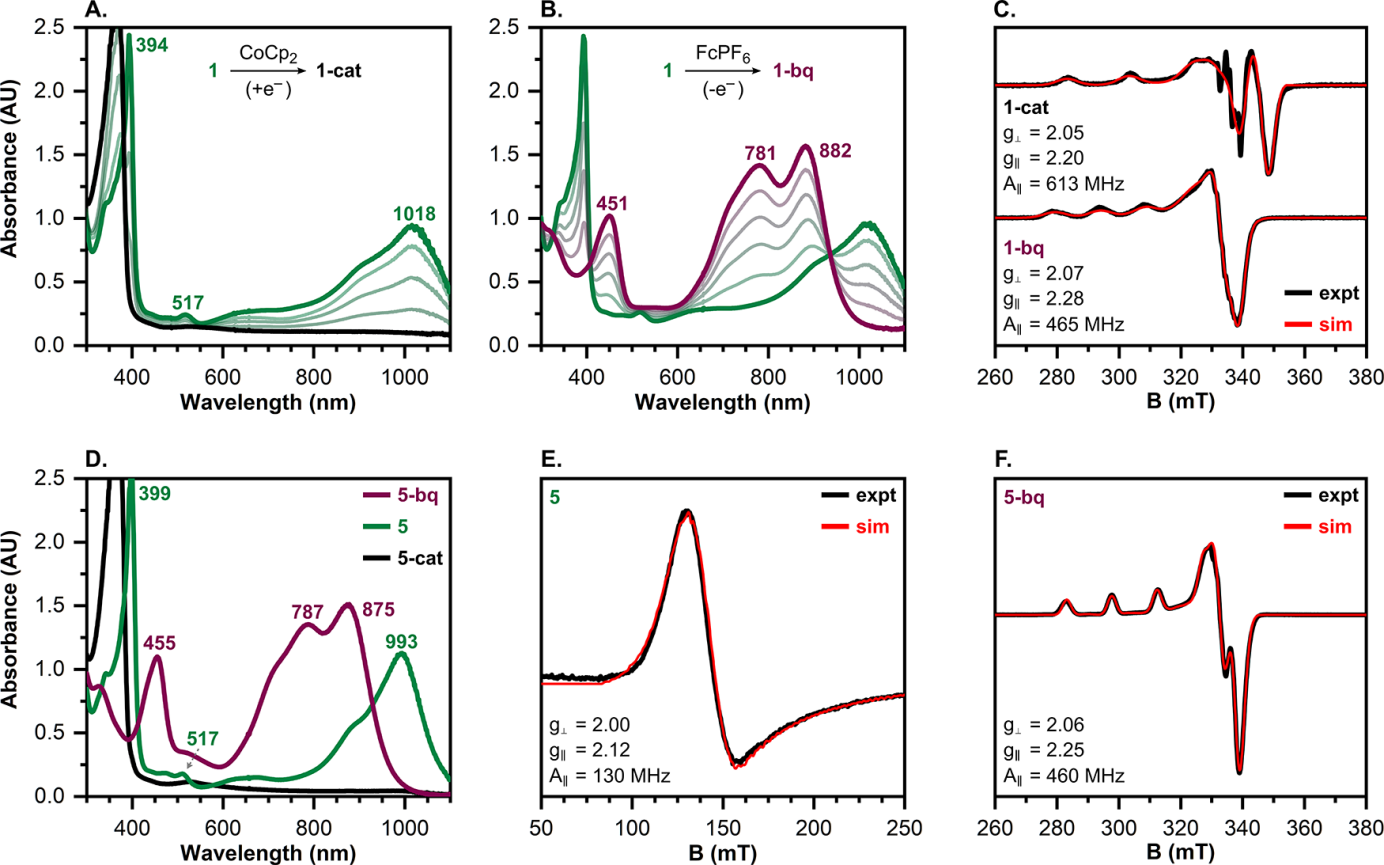
(A) UV–vis spectral changes upon reduction of **1** with 1 equiv. CoCp_2_ (0.25 equiv. added per spectrum) to generate **1-cat**. (B) UV–vis spectral changes upon oxidation of **1** with 1 equiv. FcPF_6_ (0.2 equiv. added per spectrum) to generate **1-bq**. (C) Experimental perpendicular mode EPR spectra (black traces) and simulations (red traces) of **1-cat** and **1-bq** in frozen DMF solutions (Cu = 2 mM). (D) UV–vis spectra of **5** (green trace), **5-bq** (maroon trace), and **5-cat** (black trace), generated by the addition of 1 equiv. FcPF_6_ and CoCp_2_ to **5**, respectively. (E) The experimental parallel mode EPR spectrum (black trace) and simulation (red trace) of a frozen toluene solution of **5** ([Cu] = 10 mM). (F) The experimental perpendicular mode EPR spectrum (black trace) and simulation (red trace) of a frozen 1:1 (v/v) DMF/toluene solution of **5-bq** (generated by addition of 1 equiv. FcPF_6_ to **5**; [Cu] = 10 mM). Note: All UV–vis spectra were recorded in DMF at 25 °C under argon ([Cu] = 0.125 mM). EPR spectra were recorded in perpendicular mode at 19 K and in parallel mode at 5 K.

Oxidation of **1** with one equivalent of FcPF_6_ led to the isosbestic formation of an intensely green-colored species **1-bq** with strong absorption features at 451 nm (ε = 8450 M^–1^cm^–1^), 781 nm (ε = 11850 M^–1^cm^–1^), and 882 nm (ε = 12900 M^–1^cm^–1^) (Figure 8B). These intense absorptions are typical of complexes containing the [^bq^ONO]^−^ ligand.^11,15,21,25^ Given these characteristic absorptions, the oxidized complex **1-bq** is assigned as a Cu^II^–iminobenzoquinonate complex. Similar spectra were obtained upon oxidation of **2**–**5** with FcPF_6_, albeit with slight shifts in their λ_max_ values (Figures 8D and S8–S10 and Table S16). These oxidized species (e.g., **2-bq**, **3-bq**, etc.) are likewise formulated as Cu^II^–iminobenzoquinonate complexes.

### EPR Spectroscopy

X-band electron paramagnetic resonance spectra were collected on frozen solutions of complexes **1–3** and **5** in DMF, toluene, and DMF/toluene mixtures. In all cases, the four-coordinate complexes **1–3** were EPR silent in both perpendicular and parallel modes, consistent with a diamagnetic *S* = 0 ground state. The observed diamagnetism suggests the unpaired spins of the Cu^II^ center (*S*_Cu_ = 1/2) and the [^sq^ONO]^2–^ ligand (*S*_ONO_ = 1/2) are coupled in an antiferromagnetic fashion.^22^ However, while the lack of EPR signals alone is not definitive evidence of a Cu^II^–iminosemiquinonate electronic state (as the valence tautomers (^bq^ONO)­Cu^I^(L) and (^cat^ONO)­Cu^III^(L) would also be EPR silent), the characteristic UV–vis spectra of these complexes provided additional support for this assignment. In contrast to the four-coordinate complexes, five-coordinate complex **5** displayed a broad EPR signal at *g*_∥_ = 2.12 in parallel mode (Figure 8E), indicative of a paramagnetic *S* = 1 ground state arising from the ferromagnetic coupling of the unpaired spins on the Cu^II^ center and [^sq^ONO]^2–^ ligand.

The one-electron reduced and oxidized forms of **1** and **5** were characterized by perpendicular mode EPR spectroscopy (Figure 8C,F). The EPR spectra of **1-cat** and **1-bq** were collected on frozen DMF solutions. Due to the relatively poor solubility of **5** in DMF at EPR concentrations, spectra of **5-cat** and **5-bq** were collected on frozen 1:1 (v/v) DMF/toluene mixtures. Axial resonances typical of Cu^II^ ions were observed for these species, providing further evidence that the redox events are ligand-centered. The g values and hyperfine coupling constants (*A*_∥_) for the oxidized complexes **1-bq** (*g*_⊥_ = 2.07, *g*_∥_ = 2.28, and *A*_∥_ = 465 MHz) and **5-bq** (*g*_⊥_ = 2.06, *g*_∥_ = 2.25, and *A*_∥_ = 460 MHz) are comparable, suggesting similar electronic structures. In contrast, the reduced complex **1-cat** exhibits a significantly larger *A*_∥_ value (613 MHz), indicating a greater localization of the unpaired spin on the Cu^II^ center. The EPR spectrum of **5-cat** showed the presence of at least three different Cu^II^ species (Figure S11); however, the complexity of the spectrum precluded the determination of any EPR parameters.

### Evans Method Magnetic Susceptibility

Magnetic susceptibilities of the complexes were measured in solution using the Evans method. In C_6_D_6_ at 298 K, μ_eff_ values of 2.09–2.52 μ_B_ were measured for **1**–**3** (Table S17). These values fall between the theoretical spin-only μ_eff_ values for *S* = 1/2 (1.73 μ_B_) or *S* = 1 (2.83 μ_B_) spin systems. This behavior, coupled with the absence of Cu^II^ EPR signals for **1**–**3** in perpendicular mode, suggests that the observed magnetic susceptibilities arise from an equilibrium between singlet and triplet states. Such a scenario may occur when the energy gap between singlet and triplet states is small enough to permit the thermal population of both spin states at room temperature. In contrast, complex **5** has a μ_eff_ of 2.95 μ_B_, slightly higher than the theoretical spin-only value of 2.83 μ_B_ for an *S* = 1 spin state, but within the 5% error margin typically associated with Evans method measurements.^39^ Variable-temperature Evans method experiments showed that the solution magnetic moment of **1** was temperature-independent over the range 280–333 K, while **5** exhibited a slight temperature-dependent decrease in μ_eff_ over the same temperature range (Figure S12). Similar magnetic behavior has also been reported for metal complexes with ONO and related ligands in solution using the Evans method.^31^

### Computational Studies

DFT calculations were performed to investigate the electronic structures of complex **1** and its oxidized and reduced forms (**1-bq** and **1-cat**, respectively; Figure 9). For complex **1**, C_S_ symmetry was enforced to obtain optimized structures for both the open-shell singlet (**1** ^**S**^) and triplet (**1** ^**T**^) spin states. The oxidation states of the metal and the ONO ligand were determined by evaluating the d-orbital occupancies, spin density plots, and the metrical oxidation state (MOS) values.

**Figure 9:**
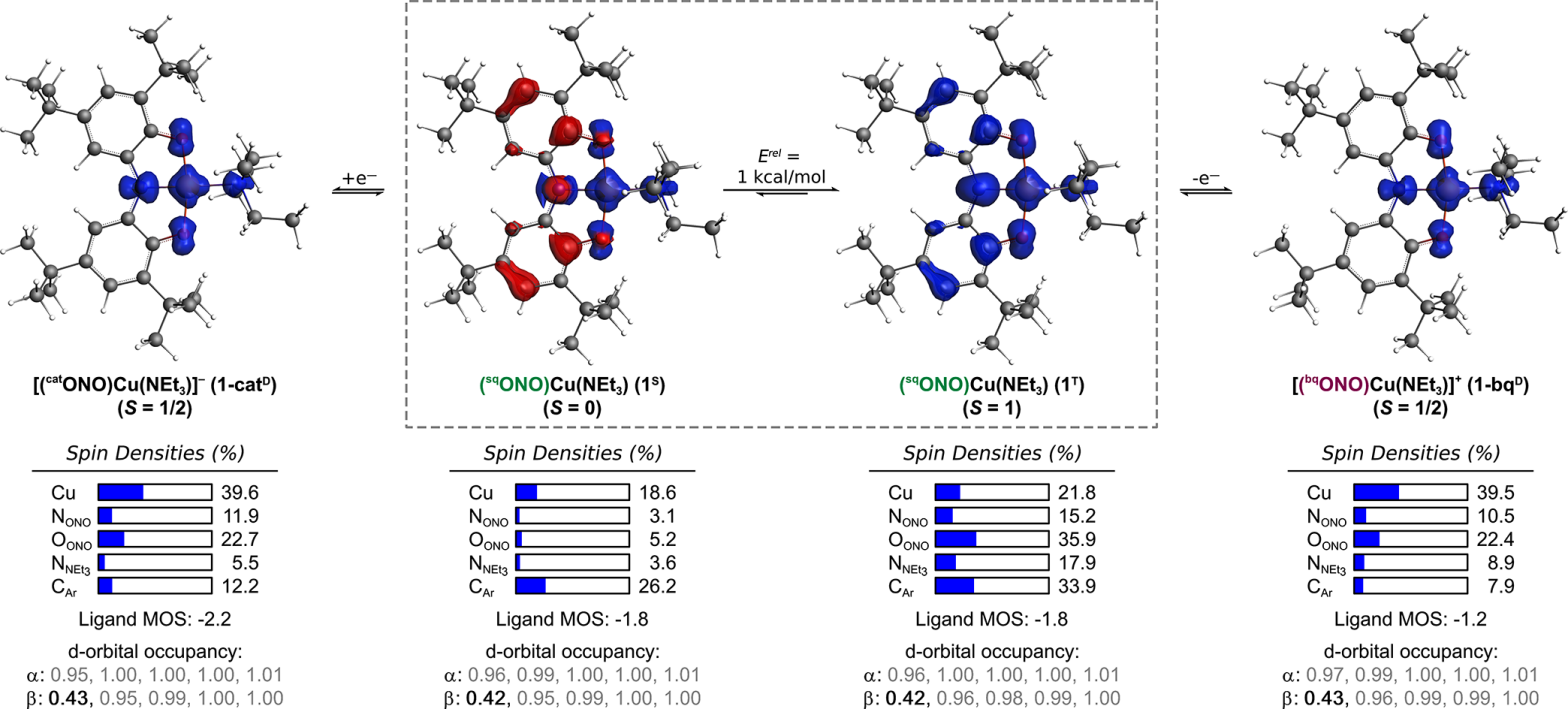
DFT-optimized structures, spin density plots, d-orbital occupancies, and ONO ligand metrical oxidation states (MOS) for **1-cat** (doublet state, **1-cat** ^**D**^), **1** in open-shell singlet (**1** ^**S**^) and triplet states (**1** ^**T**^), and **1-bq** (doublet state, **1-bq** ^**D**^).

Both **1** ^**S**^ and **1** ^**T**^ exhibit d-orbital occupancies consistent with Cu^II^ d^9^ centers (*note:* d-orbital occupancy values below 0.7 indicate unfilled orbitals),^40^ suggesting a Cu^II^-iminosemiquinone electronic structure for both spin states. Additionally, the spin density plots for **1** ^**S**^ and **1** ^**T**^ depict substantial spin density on the Cu center and the ONO ligand, consistent with the Cu^II^ ligand-radical complexes. The MOS values of the ligands of the **1** ^**S**^ and **1** ^**T**^ (determined from the computed intraligand bond lengths) align with the experimental value obtained by SC-XRD (MOS^DFT^ = MOS^SC‑XRD^ = −1.8), supporting their assignment as [^sq^ONO]^2–^ ligands.

The relative energy (*E* ^rel^) between the **1** ^**S**^ and **1** ^**T**^ structures is small (1 kcal·mol^–1^), with the triplet spin state slightly lower in energy than the open-shell singlet. While the assignment of a triplet ground state to **1** contradicts the diamagnetic ground state determined by EPR, the small *E* ^rel^ supports the idea that the magnetic susceptibility measured by the Evans method may reflect a thermal equilibrium between singlet and triplet states.

Computations on **1-cat** and **1-bq** in the doublet (*S* = 1/2) and quadruplet (*S* = 3/2) spin states were also carried out (Figures 9 and S13 and S14). For both systems, the doublet state (**1-cat** ^**D**^ and **1-bq** ^**D**^) is substantially lower in energy than the corresponding quadruplet states (*E* ^rel^ > 10 kcal·mol^–1^, Table S18). The computed d-orbital occupancies and spin density plots for **1-cat** ^**D**^ and **1-bq** ^**D**^ indicate that both complexes contain d^9^ Cu^II^ centers, with spin density localized primarily on the Cu center. The ligand MOS values varied upon reduction of **1** to **1-cat** ^**D**^ (from −1.8 to −2.2) and upon oxidation of **1** to **1-bq** ^**D**^ (from −1.8 to −1.2), suggesting that these redox events are ligand-based.

Symmetry-enforced structures (C_S_ symmetry) of complex **5** in the open-shell singlet (**5** ^**S**^) and triplet (**5** ^**T**^) spin states were computed (Figure 10). The d-orbital occupancies and spin density plots for both states support their assignment as Cu^II^ ligand-radical complexes. The triplet state **5** ^**T**^ is 1.8 kcal·mol^–1^ lower in energy than open-shell singlet **5** ^**S**^, consistent with the *S* = 1 ground state observed by EPR and in agreement with magnetic susceptibility measurements.

**Figure 10:**
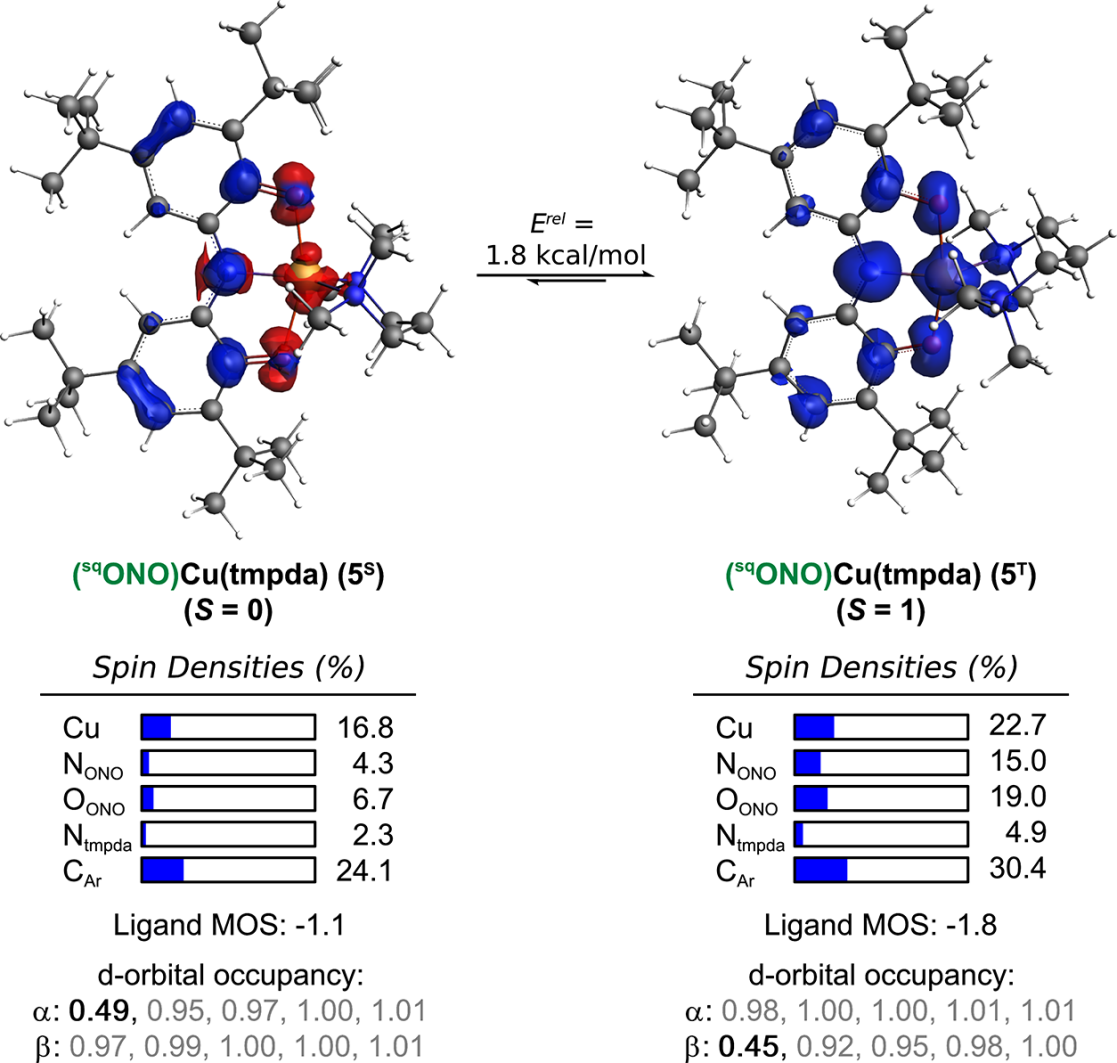
DFT-optimized structures, spin density plots, d-orbital occupancies, and ONO ligand metrical oxidation state (MOS) for **5** in the open-shell singlet (**5** ^**S**^) and triplet (**5** ^**T**^) spin states.

The MOS of the ONO ligand in **5** ^**T**^ matches the value obtained by SC-XRD (MOS^DFT^ = MOS^SC‑XRD^ = −1.8), consistent with a [^sq^ONO]^2–^ ligand. In contrast, the apparent oxidation state of the ONO ligand in **5** ^**S**^ is [^bq^ONO]^−^ (MOS = −1.1). The assignment of **5** ^**S**^ as the valence tautomer (^bq^ONO)­Cu^I^(tmpda), however, is not supported by the computed d-orbital occupancy or spin density plot. Compared to **5** ^**T**^, the ONO ligand in **5** ^**S**^ is more quinone-like, with an average C–O bond contraction of 0.042 Å and average C1–C2 and C2–C3 bond elongations of 0.032 and 0.024 Å, respectively, contributing to the increased MOS value (Figure S22).

Finally, we computed symmetry-enforced structures of complexes **2**–**4** in both open-shell singlet and triplet spin states (see Supporting Information). In all cases, the triplet states were lower in energy than the corresponding open-shell singlets. Solid-state SQUID measurements previously determined an *S* = 1 ground state for complex **4**,^19^ consistent with the DFT calculations. However, the assignment of a triplet ground state to four-coordinate complexes **2** and **3** is not supported by EPR experiments.

As with complex **1**, the calculated *E* ^rel^ between the singlet and triplet states (Table S18) for the four-coordinate complexes **2** and **3** was small (∼1 kcal·mol^–1^), supporting the magnetic susceptibility data measured by the Evans method. A slightly greater *E* ^rel^ was calculated for five-coordinate complex **4** (1.7 kcal·mol^–1^), comparable to **5** (1.8 kcal·mol^–1^).

The experimentally determined ONO intraligand bond lengths and MOS values from SC-XRD are best reproduced in the triplet structures of **2**–**4** (Table S18 and Figures S20 and S21). On the other hand, the open-shell singlet structures of complexes **3** and **4** exhibit pronounced changes in ligand MOS values: complex **3** shows an increase to −0.8 (more quinone-like, Figure S20), while **4** shows a decrease to −2.7 (more catecholate-like, Figure S21).

### Contextualization of Our Findings

Borovik and co-workers recently reported the synthesis and characterization of a series of Cu-radical complexes in which the ground spin state was influenced by the identity of the ancillary ligand (Figure 11A).^34^ The authors concluded that variations in the number of H-bonding interactions led to changes in the metal–ligand angles, thereby tuning the overlap between the Cu d-orbitals and the redox-active ligand orbitals, resulting in either a ferromagnetic (poor overlap, *S* = 1) or an antiferromagnetic (good overlap, *S* = 0) coupling of the unpaired spins of the Cu^II^ center and the [^sq^NNN′]^2–^ ligand.

**Figure 11:**
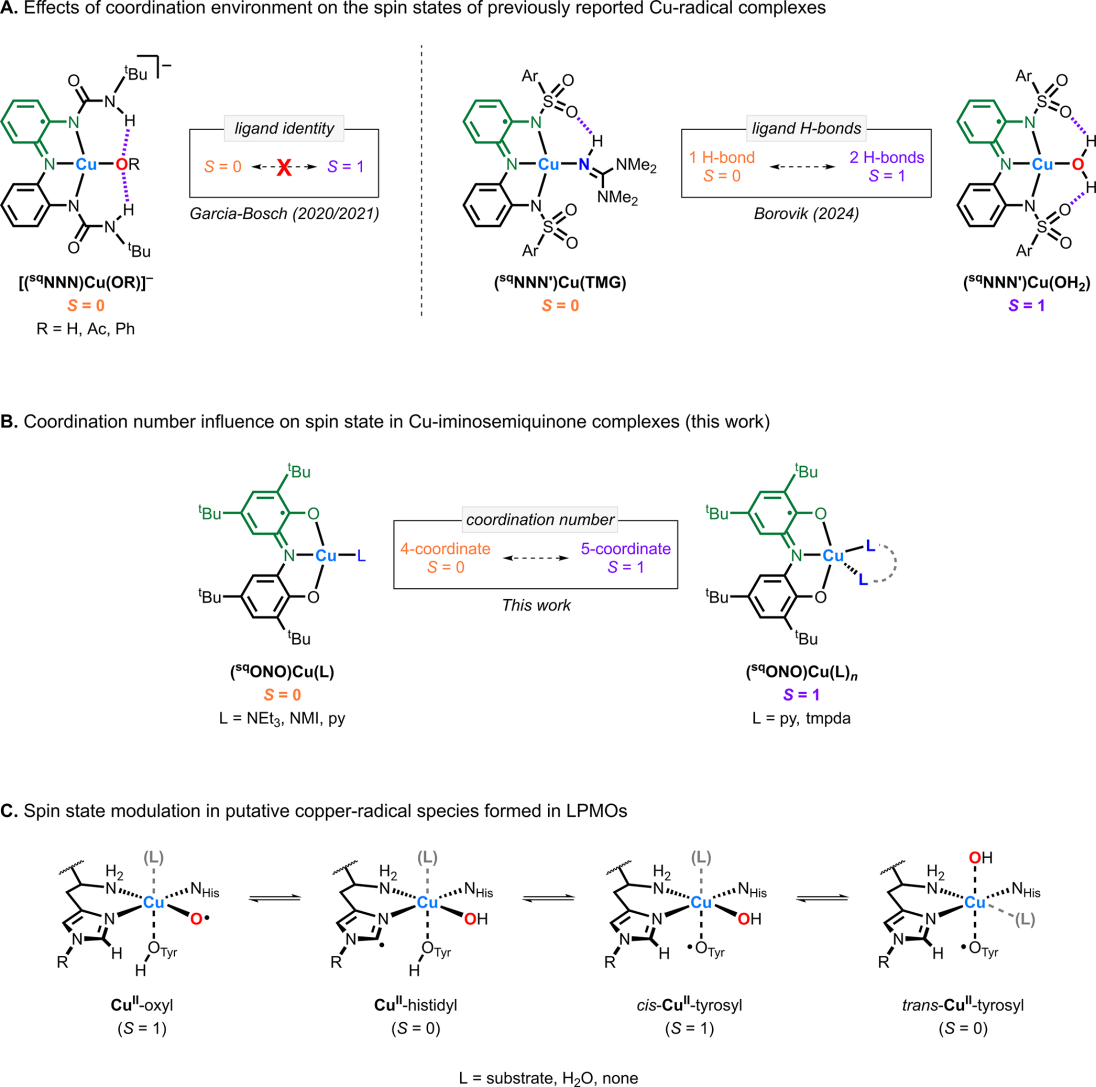
(A) Effects of changes in coordination environment on the spin states of previously reported Cu^II^ ligand-radical complexes. (B) The effect of coordination number on the spin state of the Cu-iminosemiquinone complexes described in this work. (C) Modulation of the spin state of putative Cu-radical species in LPMOs.

Our group has previously studied Cu-radical complexes featuring a similar tridentate [^sq^NNN]^2–^ ligand scaffold with various ancillary anionic ligands (hydroxide, acetate, phenolate).^41^ In these systems, all [(^sq^NNN)­Cu­(OR)]^−^ complexes were diamagnetic over a broad temperature range (∼10 K to room temperature), suggesting that changes in the identity of the ancillary ligand alone were insufficient to induce a paramagnetic ground state. In the present work, we demonstrate that the ground spin state of Cu-radical complexes can be modulated by the coordination number (four- vs five-coordinate), with the four-coordinate species favoring the singlet (*S* = 0) ground state and the five-coordinate species adopting a triplet (*S* = 1) ground state. (Figure 11B). This finding presents a distinct yet complementary strategy for tuning the spin states of Cu complexes with redox-active ligands.

In LPMOs, it has been proposed that small changes in the coordination sphere of the Cu active site result in changes in the spin state of the reaction intermediates, which may play a role in controlling their reactivity (oxygenase vs oxidase, see Figure 11C). The synthetic model systems studied by Borovik’s group and our lab (Figure 11A,B) provide evidence of how these enzymes can tune their electronic structure and, potentially, their reactivity.

## Conclusions

In this work, we report a modified one-pot synthesis of a family of Cu-iminosemiquinone complexes featuring various ancillary amine ligands with the general formula (^sq^ONO)­Cu­(L) (L = NEt_3_, NMI, py, tmpda). Electrochemical studies revealed that these complexes can reversibly access three distinct molecular oxidation states. Spectroscopic characterization and DFT calculations indicate that these redox events are ligand-centered. The one-electron oxidized and reduced forms of (^sq^ONO)­Cu­(tmpda) were isolated and structurally characterized by SC-XRD, revealing changes in ONO intraligand bond lengths of the ligand consistent with ligand oxidation and reduction. To the best of our knowledge, this electron-transfer series represents one of the few reported examples of a heteroleptic copper complex bearing identical supporting ligands for which three oxidation states have been structurally characterized.

Magnetic susceptibility measurements and computations indicate that the singlet (*S* = 0) and triplet (*S* = 1) spin states of the (^sq^ONO)­Cu­(L) complexes are close in energy, similar to the Cu^II^-tyrosyl intermediates of galactose oxidase^41^ and LPMOs.^42^ The ground spin state of the Cu-iminosemiquinone species is influenced by the coordination number, with four-coordinate complexes favoring an *S* = 0 ground state and five-coordinate complexes adopting an *S* = 1 ground state.

Future studies will investigate the effects of the ancillary amine ligand on the kinetics and thermodynamics of proton-coupled electron transfer (PCET), as well as the catalytic activity of the (^sq^ONO)­Cu­(L) complexes in alcohol dehydrogenation reactions.

## Experimental Section

### General Considerations

Air-free handling of the copper complexes was performed inside an MBRAUN UNIlab Pro SP glovebox system with a N_2_ working gas. Combustion analysis was performed by Midwest Micro Lab (Indianapolis, IN). UV–vis spectra, EPR spectra, and cyclic voltammograms were visualized and depicted using OriginPro 2024b.^43^

### Materials

All reagents were purchased from commercial suppliers and used as received without further purification. Copper­(II) chloride dihydrate (99+%), pyridine (ACS), 3,5-di-*tert*-butylcatechol (99%), and ammonium hydroxide (28–30%, ACS) were purchased from Thermo Scientific (Waltham, MA). Triethylamine (>99.5%), *N*-methylimidazole (99%), and cobaltocene (CoCp_2_) were purchased from Sigma-Aldrich (St. Louis, MO). *N*,*N*,*N*′,*N*′-tetramethylpropane-1,3-diamine (99+%) was purchased from Acros Organics (Geel, Belgium). Ferrocenium hexafluorophosphate (FcPF_6_) was prepared according to a literature procedure.^44^ Dimethylformamide (DMF) was dried over 4 Å molecular sieves, distilled under a partial vacuum, sparged with N_2_, and stored in a glovebox before use. All other solvents were purchased at the highest level of purity and further purified, dried, and deoxygenated by passing through an MBRAUN MB SPS-7 activated alumina solvent purification system. Deuterated solvents were purchased from Cambridge Isotope Laboratories (Tewksbury, MA) and used as received.

### Crystallographic Methods

Reflection intensities were measured at 110 K using either a SuperNova diffractometer (with Atlas detector) with Mo *K*α radiation (λ = 0.71073 Å) or Cu Kα radiation (λ = 1.54178 Å) or a Rigaku XtaLAB Synergy R diffractometer (with a rotating-anode X-ray source and an HyPix-6000HE detector) with Cu Kα radiation (λ = 1.54178 Å). Data collection, refinement of cell dimensions, and data reduction were performed using CrysAlisPro (see the Supporting Information for more details).

### Electrochemical Methods

Electrochemical measurements were collected using a CH Instruments 620E Electrochemical Workstation (CH Instruments, Austin, TX) with a standard three-electrode setup consisting of a 4 mm glassy carbon working electrode, platinum wire counter electrode, and a nonaqueous Ag/Ag^+^ reference electrode (CHI112, CH Instruments) containing 10 mM AgNO_3_ in CH_3_CN. Cyclic voltammograms were measured on DMF solutions of the Cu complexes ([Cu] = 1 mM, [NBu_4_PF_6_] = 100 mM; prepared in a glovebox) at room temperature under a gentle flow of argon gas.

### Spectroscopic Methods

UV–vis spectra were collected using an Agilent Cary 8454 diode array spectrophotometer equipped with a Unisoku CoolSpeK cryostat (UNISOKU Co., Hirakata, Japan) for temperature control and magnetic stirring.

Data were collected using the Agilent UV–visible ChemStation software, and spectra were processed using the uv_pro Python library.^45^ All spectra were measured at 25 °C under a flow of argon gas using a custom-made 1 cm path quartz Schlenk cuvette. X-band EPR spectra of frozen solutions were recorded on a Bruker ELEXSYS spectrometer equipped with an Oxford liquid helium cryostat and a Bruker bimodal cavity. The microwave frequency was calibrated with a frequency counter, and the magnetic field was measured with an NMR gaussmeter. The sample temperature was calibrated against a CX-1050 Cernox sensor mounted inside an EPR tube. A modulation amplitude of 0.5 mT and frequency of 100 kHz was used for all EPR spectra. NMR spectra were collected using a Bruker Avance NEO 500 MHz NMR spectrometer to acquire spectra with 16 cumulative scans.

### General Preparation of (^sq^ONO)­Cu­(L) Complexes (L = NEt_3_, NMI, py, tmpda)

3,5-Di-*tert*-butylcatechol (1.11 g, 5 mmol, 2 equiv), acetonitrile (9 mL), and the appropriate amine base (L, 1.2 equiv) were combined in a 20 mL vial equipped with a magnetic stir bar. Copper­(II) chloride dihydrate (426 mg, 2.5 mmol, 1 equiv) was added to the stirred solution, resulting in a rapid color change from dark yellow/brown to dark green/black. After stirring the heterogeneous mixture for 3 min, concentrated (29% w/v) aqueous ammonium hydroxide (0.36 mL, 5.52 mmol, 2.2 equiv) was added, and the mixture darkened in color. A dark blue precipitate was noted, which slowly disappeared (partially or completely) over the course of the reaction. The mixture was then either (*a*) stirred in open air for 4 h or (*b*) sparged with O_2_ for 5 min and stirred under an O_2_ atmosphere (balloon) for 4 h. The resulting dark green/black mixture was cooled to −5 °C, and the complex was collected by vacuum filtration in air (note: as the complexes are prone to aerobic oxidation in solution, the filtration should be performed expeditiously, and the use of additional solvent for transferring or rinsing the material should be avoided). The collected solid was dried under high vacuum and brought into a N_2_-filled glovebox, after which it was dissolved in diethyl ether and filtered through a glass frit to remove inorganic copper. The solvent was removed under reduced pressure to give the complex as a dark-colored powder. Crystals suitable for X-ray diffraction were obtained by the slow evaporation of a diethyl ether solution under anaerobic conditions.

#### (^sq^ONO)­Cu­(NEt_3_) (**1**)

Triethylamine (0.42 mL, 3.01 mmol, 1.2 equiv) was used. The product is a dark green powder. Yield: 0.76 g (52%) with O_2_; 0.54 g (37%) with air. This compound has been previously reported.^22^ Anal. Calc. (found) for C_34_H_55_CuN_2_O_2_·0.5Et_2_O: C, 69.53 (69.25); H, 9.44 (9.69); N, 4.77 (4.49). UV–vis (DMF) λ_max_ [nm] (ε [M^–1^cm^–1^]): 394 (18960), 517 (1670), 1018 (7540). Evans method (C_6_D_6_, 298 K) μ_eff_ [μ_B_]: 2.09.

#### (^sq^ONO)­Cu­(NMI) (**2**)

*N*-methylimidazole (0.24 mL, 3.01 mmol, 1.2 equiv) was used. The product is a dark green powder. Yield: 0.80 g (57%) with O_2_; 0.43 g (31%) with air. Anal. Calc. (found) for C_32_H_46_CuN_3_O_2_: C, 67.63 (67.49); H, 8.16 (8.48); N, 7.39 (7.58). UV–vis (DMF) λ_max_ [nm] (ε [M^–1^cm^–1^]): 393 (15400), 524 (1800), 918 (4990), 1016 (4960). Evans method (C_6_D_6_, 298 K) μ_eff_ [μ_B_]: 2.28.

#### (^sq^ONO)­Cu­(py) (**3**)

The complex was prepared in an analogous manner as described in the general synthesis above, with pyridine (9 mL) used in place of acetonitrile, serving as both the solvent and the amine ligand. The product is a dark brown powder. Yield: 0.76 g (54%) with O_2_; 0.34 g (24%) with air. Anal. Calc. (found) for C_33_H_45_CuN_2_O_2_·0.5Et_2_O·0.5H_2_O: C, 68.76 (68.37); H, 8.41 (8.25); N, 4.58 (4.66). UV–vis (DMF) λ_max_ [nm] (ε [M^–1^cm^–1^]): 394 (20000), 517 (1730), 1018 (8160). Evans method (C_6_D_6_, 298 K) μ_eff_ [μ_B_]: 2.52.

After the isolation of crude **3** from the reaction mixture by filtration, storage of the collected pyridine filtrate at – 5 °C for >48 h resulted in the crystallization of a small crop of **(** ^**sq**^ **ONO)­Cu­(py)**_**2**_ **(4)**. Dark green crystals were collected by vacuum filtration in air. Yield: 0.10 g (6%) with O_2_; 0.22 g (14%) with air. This compound has been previously reported.^19^ Anal. Calc. (found) for C_38_H_50_CuN_3_O_2_: C, 70.83 (71.29); H, 7.82 (7.85); N, 6.52 (6.17). UV–vis (DMF) λ_max_ [nm] (ε [M^–1^cm^–1^]): 394 (21000), 518 (2010), 1016 (9330). Recrystallization of **4** from diethyl ether led to the dissociation of one pyridine ligand, affording crystals of the monopyridine complex **3**.

#### (^sq^ONO)­Cu­(tmpda) (**5**)

N,N,N′,N′-tetramethylpropane-1,3-diamine (0.5 mL, 2.99 mmol, 1.2 equiv) was used. The product is a black powder. Yield: 1.00 g (65%) with O_2_; 1.10 g (72%) with air. Anal. Calc. (found) for C_35_H_58_CuN_3_O_2_·H_2_O: C, 66.26 (66.33); H, 9.53 (9.42); N, 6.62 (6.53). UV–vis (DMF) λ_max_ [nm] (ε [M^–1^cm^–1^]): 397 (20200), 510 (1570), 993 (9510). Evans method (C_6_D_6_, 298 K) μ_eff_ [μ_B_]: 2.95. EPR (parallel mode, 1:1 (v/v) DMF/toluene, 10 K): *g*_⊥_ = 2.00, *g*_∥_ = 2.12, *A*_∥_ = 130 MHz.

#### [CoCp_2_]_2_[{^cat^ONOCu}_2_(μ-tmpda)] (**5-cat**)

In a N_2_-filled glovebox, solid cobaltocene (25.4 mg, 0.13 mmol, 1.7 equiv) was added into an 8 mL scintillation vial containing a forest green solution of complex **5** (50.2 mg, 0.08 mmol) in a 4:1 (v/v) acetonitrile/toluene mixture (2.5 mL). The resulting dark purple-brown heterogeneous mixture was shaken for 2 min, and the complex was precipitated by dropwise addition of the reaction mixture into pentane (20 mL). Removal of the solvent by Pasteur pipet and subsequent drying of the precipitate under high vacuum afforded the complex as a dark purple powder (93 mg, 77%). Single crystals suitable for X-ray diffraction were obtained by layering diethyl ether on an acetonitrile/toluene mixture of the complex (prepared in the same manner as described above) in a 20 mL scintillation vial, followed by storage at −35 °C for >3 d, after which crystals formed on the vial walls. Anal. Calc. (found) for C_83_H_118_Cu_2_Co_2_N_4_O_4_: C, 67.32 (67.35); H, 8.03 (8.00); N, 3.78 (3.72).

#### [(^bq^ONOCu)­(tmpda)]­PF_6_ (**5-bq**)

In a N_2_-filled glovebox, solid FcPF_6_ (28.1 mg, 0.08 mmol, 1.0 equiv) was added into an 8 mL scintillation vial containing a forest green solution of complex **5** (51.7 mg, 0.08 mmol) in a 4:1 (v/v) acetonitrile/toluene mixture (2.5 mL). The resulting intense green mixture was shaken for 2 min and then added dropwise into hexane (20 mL) to give a dark green-brown oil. The solvent was removed by Pasteur pipet, and the residual solvent was evaporated under high vacuum, affording the complex as a green powder (54 mg, 85%). Single crystals suitable for X-ray diffraction were obtained by layering hexane on a toluene solution (filtered through a 0.45 μm PTFE syringe filter) in an NMR tube. Anal. Calc. (found) for C_83_H_118_Cu_2_Co_2_N_4_O_4_: C, 55.21 (55.49); H, 7.68 (7.73); N, 5.52 (5.50).

### UV–vis Oxidation and Reduction of Cu-Iminosemiquinone Complexes **1–5** with FcPF_6_ and CoCp_2_

In a glovebox, a Schlenk cuvette containing a magnetic stir bar was filled with DMF (2.8 mL) and sealed with a rubber septum. DMF solutions of the Cu complex (0.5 mL, 3.75 mM), FcPF_6_ (1.0 mL, 3.75 mM), and CoCp_2_ (1.0 mL, 3.75 mM) were prepared, and the solutions were taken up into gastight microsyringes (250 μL capacity). The Schlenk cuvette and gastight syringes were moved out of the glovebox, and the cuvette containing only DMF was used to blank the instrument. An aliquot (100 μL) of Cu complex solution was injected into the cell through the rubber septum. Then, aliquots (25 μL x 4) of either FcPF_6_ or CoCp_2_ solution were injected to generate the one-electron oxidized or reduced complex, respectively. The final solution had a volume of 3 mL ([Cu] = 125 μM).

### EPR Characterization of Cu-Iminosemiquinone Complexes **1–3** and **5**

In a glovebox, toluene solutions of Cu complexes **1**–**3** were prepared (1.0 mL, 6 mM). Aliquots (50 μL) of each Cu complex solution were added into separate 1 mL vials and diluted with toluene (250 μL). The final solutions had volumes of 300 μL ([Cu] = 1 mM). The solutions were transferred into quartz EPR tubes, which were moved out of the glovebox and frozen in liquid N_2_ for EPR analysis.

EPR characterization of complex **5** was performed at a higher concentration (10 mM) for an improved signal in parallel mode. The sample was prepared in an analogous manner as described above, but the initial toluene solution had a concentration of 20 mM (1.5 mL). An aliquot (300 μL) of this solution was added into a 1 mL vial and diluted with toluene (300 μL). A portion (300 μL) of the diluted solution was transferred to an EPR tube and frozen in liquid N_2_ for analysis.

### EPR Characterization of the Oxidized and Reduced Forms of **1** and **5**

In a glovebox, DMF solutions of **1** (1.0 mL, 5 mM), FcPF_6_ (1.0 mL, 10 mM), and CoCp_2_ (2.0 mL, 10 mM) were prepared. An aliquot (200 μL) of the **1** solution was added to a 1 mL vial, followed by DMF (200 μL). Then, aliquots (50 μL x 2) of either FcPF_6_ or CoCp_2_ solution were added to the vial with intermittent mixing to generate **1-bq** or **1-cat**, respectively. The final solution had a volume of 500 μL ([Cu] = 2 mM). A portion (300 μL) of the solution was transferred into a quartz EPR tube, which was moved out of the glovebox and frozen in liquid N_2_ for the EPR analysis.

Due to the poor solubility of complex **5** in DMF at EPR concentrations, characterization of **5-cat** and **5-bq** was performed with frozen DMF/toluene mixtures. A toluene solution of **5** (1.5 mL, 20 mM) and DMF solutions of FcPF_6_ (0.5 mL, 20 mM) and CoCp_2_ (0.5 mL, 20 mM) were prepared in a glovebox. An aliquot (300 μL) of the **5** solution was added into a 1 mL vial, and then aliquots (150 μL x 2) of either FcPF_6_ or CoCp_2_ solution were added to the vial with intermittent mixing to generate **5-bq** or **5-cat**, respectively. The final solutions had volumes of 600 μL ([Cu] = 10 mM). Portions (300 μL) of each solution were transferred to EPR tubes and frozen in liquid N_2_ for analysis.

### Evans Method Magnetic Susceptibility

Evans method experiments were performed using a 7 in., 5 mm o.d. NMR tube with a coaxial 3 mm o.d. NMR tube insert. In a glovebox, one drop of DCM from a Pasteur pipet was added into C_6_D_6_ (3 mL). An aliquot (600 μL) of this solvent mixture was used to prepare a solution of the Cu complex ([Cu] = 30 mM). The Cu complex solution was added to the 5 mm o.d. NMR tube, and the neat C_6_D_6_/DCM solvent mixture was added to the inner 3 mm o.d. tube. The NMR tube was capped and sealed with PTFE tape and was brought out of the glovebox for NMR analysis. The μ_eff_ of the complex was calculated from the peak separation between the DCM internal standard resonances (see the Supporting Information for more details).

## Supplementary Material



## Notes

SC-XRD analysis details; UV–vis spectra; EPR spectrum; Evans method data; and DFT calculations (PDF)

## Abbreviations

3,5-DTBC: 3,5-di-*tert*-butylcatechol
NMI: *N*-methylimidazole
py: pyridine
tmeda: *N*,*N*,*N*′,*N*′-tetramethylethane-1,2-diamine
tmpda: *N*,*N*,*N*′,*N*′-tetramethylpropane-1,3-diamine
