# Supplementary material for: Synthesis and Characterization of Copper Complexes Featuring a Redox-Active ONO Ligand in Three Molecular Oxidation States
Source: Inorg Chem. 2025 May 30;64(22):11204–18. doi: 10.1021/acs.inorgchem.5c01578 (PMC12152926; doi:10.1021/acs.inorgchem.5c01578)
Supplement: Supplementary file 1 [file ic5c01578_si_001.pdf]

Supplementary Information

for

Synthesis and Characterization of Copper Complexes Featuring a Redox-Active ONO Ligand in Three Molecular Oxidation States

David D. Hebert[†], Ankita Puri[†], Daniel Ye[†], Allison McAninch[†], Amanda Chisholm[†], Maxime A. Siegler[‡], Marcel Swart,[*,ψ] Isaac Garcia-Bosch[*,†]

[†]*Department of Chemistry, Carnegie Mellon University, Pittsburgh, Pennsylvania 15213, United States.*

[‡]*Johns Hopkins University, Baltimore, Maryland 21218, United States.*

[ψ]*University of Girona, Campus Montilivi (Ciències), IQCC, Girona, Spain; ICREA, Pg. Lluís Companys 23, 08010, Barcelona, Spain.*

[*]*Corresponding Authors*

igarciab@andrew.cmu.edu (Isaac Garcia-Bosch); marcel.swart@udg.edu (Marcel Swart)

Contents

1. Synthesis of Cu-iminosemiquinone Complexes 3
2. Crystallography 5
   2.1 Atom Numbering Scheme 5
   2.2 SC-XRD Structures 6
3. UV-vis Spectra 27
   3.1 UV-vis Oxidation and Reduction of Cu-iminosemiquinone Complexes 27
   3.2 UV-vis Absorption Data 30
4. EPR Spectroscopy 32
5. Evans Method Magnetic Susceptibility 33
6. DFT Calculations 35
   6.1 Spin Density Plots 36
   6.2 Spin States, Electronic Structures, and Structural Parameters 43
   6.3 Optimized Cartesian Coordinates 49
7. References 63
8. Appendix 65
   8.1 Abbreviations 65

1. Synthesis of Cu-iminosemiquinone Complexes

The (^{sq}ONO)Cu(L) complexes (L = NEt_3, NMI, tmpda) described in this study were prepared using a modified procedure for the synthesis of (^{sq}ONO)Cu(py)$_2$ reported by Pierpont and coworkers.[1] In a typical reaction, the desired amine ligand (L), 3,5-di-*tert*-butylcatechol (3,5-DTBC), $CuCl_2·2H_2O$, and aqueous NH_4OH were combined in acetonitrile and stirred under an aerobic atmosphere (air or O_2) for 4 h at room temperature. Cooling the reaction mixture to −5 °C and subsequent vacuum filtration afforded the complexes as dark powders. Purification of the Cu-iminosemiquinone complexes was achieved by dissolving the crude products in diethyl ether under an inert atmosphere, followed by filtration and evaporation of the solvent under reduced pressure (**Scheme S1A**).

The pyridine complexes (^{sq}ONO)Cu(py) and (^{sq}ONO)Cu(py)$_2$ were synthesized using the same procedure described above, but with pyridine as the solvent. With these conditions, the initial crude product obtained was the dipyridine complex (^{sq}ONO)Cu(py)$_2$.[1] However, purification of the crude complex from diethyl ether led to loss of one pyridine ligand, yielding the monopyridine complex (^{sq}ONO)Cu(py). A small crop of the dipyridine complex was recovered from the pyridine filtrate obtained after filtering the crude reaction mixture (**Scheme S1B**).

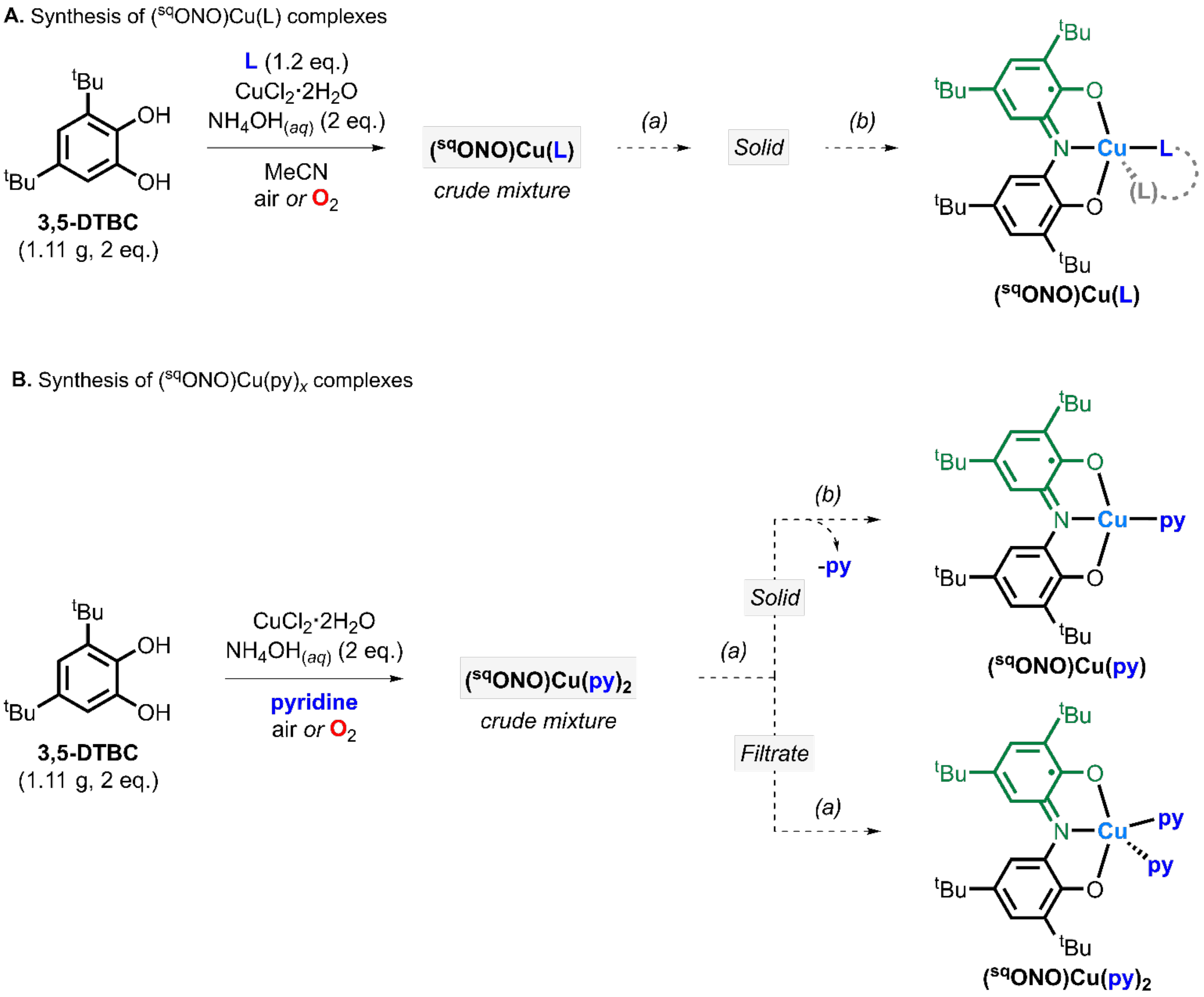

Scheme S1. Detailed synthetic scheme for the synthesis of the (^{sq}ONO)Cu(L) complexes described in this study. Syntheses and isolation of crude products were performed under aerobic conditions. Purification of crude products was performed under an inert atmosphere (glovebox). Conditions: *(a)* Cool to −5 °C, then filter in air; *(b)* Dissolve in Et_2O and filter under N_2, then evaporate under reduced pressure.

2. Crystallography

All reflection intensities were measured using either a SuperNova diffractometer (equipped with Atlas detector) with Mo *K*α radiation (λ = 0.71073 Å) at 110(2) K or using a Rigaku XtaLAB Synergy R diffractometer (equipped with a rotating-anode X-ray source and HyPix-6000HE detector) with Cu *K*α radiation (λ = 1.54178 Å) at 110.00(10) K. Crystals were first deposited onto a microscope slide with Parabar 10312 and cooled under a cold N_2(g) stream to prevent decomposition of the crystals. One single crystal was then quickly picked and mounted on the diffractometer while being flash-cooled at 110 K. The temperature of the data collection was controlled using the system Cryojet (Oxford Cryosystems, Long Hanborough, England) for the SuperNova diffractometer, and using the system Cryostream 1000 from Oxford Cryosystems for the Rigaku XtaLAB Synergy R diffractometer. Data collection, refinement of cell dimensions, and data reduction were performed using the program CrysAlisPro. The following computer programs were used: *CrysAlisPro* 1.171.42.49 (Rigaku OD, 2022), *SHELXT-2018/2* (Sheldrick, 2018), *SHELXS2018/*3 (Sheldrick, 2018), *SHELXL2018/*3 (Sheldrick, 2018), *SHELXL-2019/3* (Sheldrick, 2018), *SHELXTL* v6.10 (Sheldrick, 2008).

2.1 Atom Numbering Scheme

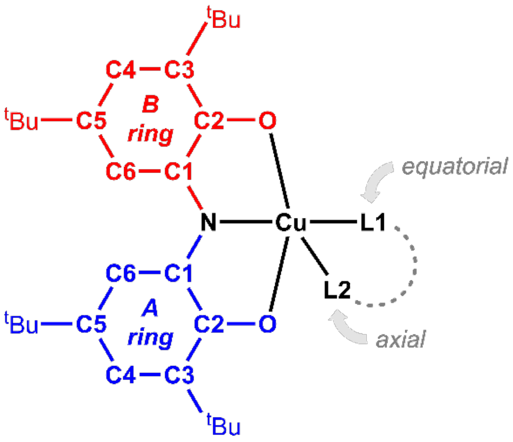

Scheme S2. Atom numbering scheme for the **(ONO)Cu(L)** complexes.

Selected bond lengths and angles are reported according to the atom numbering scheme above.

2.2 SC-XRD Structures

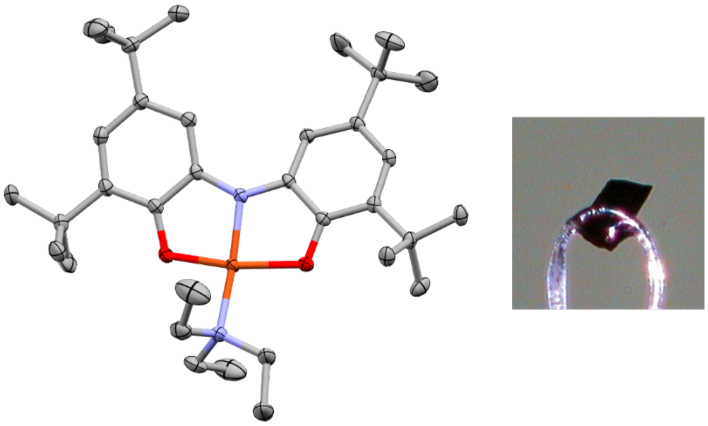

Figure S1. Displacement ellipsoid plot (50% probability level) of **(^{sq}ONO)Cu(NEt_3) (1)** at 110 K. Selected crystallographic bond lengths and bond angles are given in **Table S1**. A photograph of the single crystal used for analysis is shown on the right.

Crystallization: Crystals of **1** suitable for X-ray diffraction analysis were grown by slow evaporation of an Et_2O solution under anaerobic conditions.

Crystal Structure Solving: Reflection intensities were measured at 110(2) K using a SuperNova diffractometer (equipped with Atlas detector) with Cu *K*α radiation (λ = 1.54178 Å). The structure was solved with the program SHELXS-2018/3 (Sheldrick, 2018) and was refined on F^2 with SHELXL-2018/3 (Sheldrick, 2018). Analytical numeric absorption correction using a multifaceted crystal model was applied using the program CrysAlisPro. The H atoms were placed at calculated positions using the instructions AFIX 23, AFIX 43 or AFIX 137 with isotropic displacement parameters having values 1.2 or 1.5 U_{eq} of the attached C atoms.

Structural Description: The structure is ordered. The absolute configuration has been established by anomalous-dispersion effects in diffraction measurements on the crystal, and the Flack and Hooft parameters refine to -0.026(8) and -0.026(7), respectively.

Table S1. Selected crystallogrpahic bond lengths, angles, and geometry index for **(^{sq}ONO)Cu(NEt_3)** (**1**).

**Bond**	**Bond Length (Å)**	
	A ring	***B ring***
**C1–N**	1.380(3)	1.351(3)
**C2–O**	1.316(3)	1.307(3)
**C1–C2**	1.433(3)	1.443(3)
**C2–C3**	1.426(3)	1.429(3)
**C3–C4**	1.384(3)	1.381(3)
**C4–C5**	1.410(3)	1.429(3)
**C5–C6**	1.384(3)	1.371(3)
**C6–C1**	1.407(3)	1.416(3)
**Cu–O**	1.924(2)	1.958(2)
**Cu–N**	1.906(2)	
**Cu–L1**	2.012(2)	
**Bond Angle**	**Angle (°)**	
**O–Cu–O**	160.25(7)	
**N–Cu–L1**	168.25(8)	
**O–Cu–N**	83.75(7)	
**O–Cu–N**	83.41(7)	
**O–Cu–L1**	96.00(7)	
**O–Cu–L1**	99.61(7)	
**Geometry Index**	**Index**	
**τ_4**	0.22	

Table S2. Crystallographic data for **(^{sq}ONO)Cu(NEt$_3$)** (**1**).

(^{sq}ONO)Cu(NEt$_3$) (1) Crystal data	
Chemical formula	$C_{34}H_{55}CuN_2O_2$
M_r	587.34
Crystal system, space group	Orthorhombic, *Iba*2
Temperature (K)	110
a, *b*, *c* (Å)	23.1863 (3), 25.0992 (3), 11.72083 (16)
V (Å^3)	6821.03 (15)
Z	8
Radiation type	Cu $K\alpha$
μ (mm^{-1})	1.12
Crystal size (mm)	0.24 × 0.12 × 0.04
**Data collection**	
Diffractometer	SuperNova, Dual, Cu at zero, Atlas
Absorption correction	Analytical *CrysAlis PRO* 1.171.42.49 (Rigaku Oxford Diffraction, 2022) Analytical numeric absorption correction using a multifaceted crystal model based on expressions derived by R.C. Clark & J.S. Reid. (Clark, R. C. & Reid, J. S. (1995). Acta Cryst. A51, 887-897) Empirical absorption correction using spherical harmonics, implemented in SCALE3 ABSPACK scaling algorithm.
T_{min}, T_{max}	0.844, 0.965
No. of measured, independent and observed [$I > 2\sigma(I)$] reflections	27766, 6435, 6216
R_{int}	0.028
$(\sin \theta/\lambda)_{max}$ (Å^{-1})	0.617
**Refinement**	
$R[F^2 > 2\sigma(F^2)]$, $wR(F^2)$, S	0.025, 0.068, 1.08
No. of reflections	6435
No. of parameters	367
No. of restraints	1
H-atom treatment	H-atom parameters constrained
$\Delta\rho_{max}$, $\Delta\rho_{min}$ (e Å^{-3})	0.21, -0.21
Absolute structure	Flack x determined using 2721 quotients [(I+)-(I-)]/[(I+)+(I-)] (Parsons, Flack and Wagner, Acta Cryst. B69 (2013) 249-259).
Absolute structure parameter	-0.026 (8)

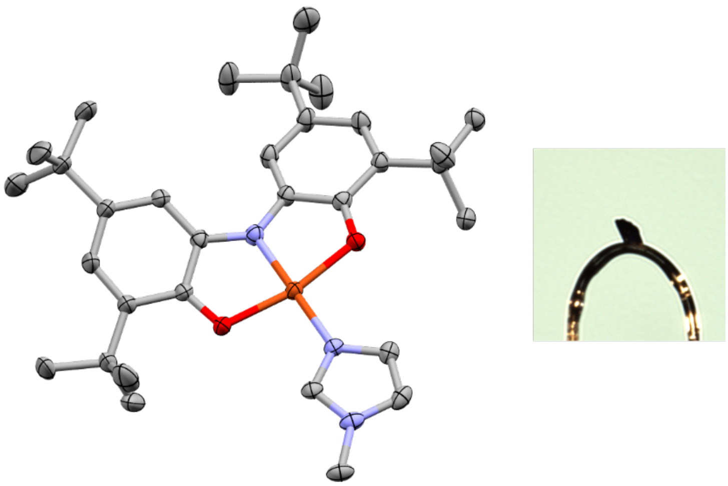

Figure S2. Displacement ellipsoid plot (50% probability level) of **(^{sq}ONO)Cu(NMI) (2)** at 110 K. Disorder was omitted for clarity. Selected crystallographic bond lengths and bond angles are given in **Table S3**. A photograph of the single crystal used for analysis is shown on the right.

Crystallization: Crystals of **2** suitable for X-ray diffraction analysis were grown by slow evaporation of an Et_2O solution under anaerobic conditions.

Crystal Structure Solving: Reflection intensities were measured at 110.00(10) K using a Rigaku XtaLAB Synergy R (equipped with a rotating-anode X-ray source and HyPix-6000HE detector) with Cu *K*α radiation (λ = 1.54178 Å). The structure was solved with the program SHELXT-2018/2 (Sheldrick, 2018) and was refined on F^2 with SHELXL-2019/3 (Sheldrick, 2018). Analytical numeric absorption correction using a multifaceted crystal model was applied using the program CrysAlisPro. The H atoms were placed at calculated positions using the instructions AFIX 43 or AFIX 137 with isotropic displacement parameters having values 1.2 or 1.5 U_{eq} of the attached C atoms.

Structural Description: The structure is mostly ordered. The coordinated N-methylimidazole is disordered over two orientations, and the occupancy factor of the major component of the disorder refines to 0.633(6). The crystal that was mounted on the diffractometer was non-merohedrally twinned, and the two twin components are related by a twofold rotation along [-0.67 0.00 0.74]. The BASF scale factor refines to 0.3806(13).

Table S3. Selected crystallographic bond lengths, angles, and geometry index for **(^{sq}ONO)Cu(NMI)** (**2**).

	Bond Length (Å)	
**Bond**	***A ring***	***B ring***
**C1–N**	1.365(3)	1.357(3)
**C2–O**	1.318(2)	1.316(2)
**C1–C2**	1.431(3)	1.437(3)
**C2–C3**	1.426(3)	1.423(3)
**C3–C4**	1.394(3)	1.385(3)
**C4–C5**	1.416(3)	1.419(3)
**C5–C6**	1.373(3)	1.367(3)
**C6–C1**	1.410(3)	1.409(3)
**Cu–O**	1.958(2)	1.962(2)
**Cu–N**	1.899(1)	
**Cu–L1**	1.937(1)	
**Bond Angle**	**Angle (°)**	
**O–Cu–O**	166.44(7)	
**N–Cu–L1**	179.54(8)	
**O–Cu–N**	83.34(7)	
**O–Cu–N**	83.15(7)	
**O–Cu–L1**	96.92(7)	
**O–Cu–L1**	96.59(7)	
**Geometry Index**	**Index**	
**τ_4**	0.10	

Table S4. Crystallographic data for **(^{sq}ONO)Cu(NMI)** (**2**).

(^{sq}ONO)Cu(NMI) (2) Crystal data	
Chemical formula	$C_{32}H_{46}CuN_3O_2$
M_r	568.26
Crystal system, space group	Monoclinic, *C*2/*c*
Temperature (K)	110
a, *b*, *c* (Å)	31.5176 (5), 6.78132 (10), 30.3676 (5)
β (°)	104.3813 (18)
V (Å^3)	6287.11 (18)
Z	8
Radiation type	Cu *K*α
μ (mm^{-1})	1.21
Crystal size (mm)	0.09 × 0.07 × 0.02
**Data collection**	
Diffractometer	XtaLAB Synergy R, HyPix
Absorption correction	Analytical *CrysAlis PRO* 1.171.42.80a (Rigaku Oxford Diffraction, 2023) Analytical numeric absorption correction using a multifaceted crystal model based on expressions derived by R.C. Clark & J.S. Reid. (Clark, R. C. & Reid, J. S. (1995). Acta Cryst. A51, 887-897) Empirical absorption correction using spherical harmonics, implemented in SCALE3 ABSPACK scaling algorithm.
T_{min}, T_{max}	0.926, 0.983
No. of measured, independent and observed [$I > 2\sigma(I)$] reflections	39820, 7021, 5152
R_{int}	0.036
$(\sin \theta/\lambda)_{max}$ (Å^{-1})	0.616
**Refinement**	
$R[F^2 > 2\sigma(F^2)]$, $wR(F^2)$, S	0.033, 0.084, 0.92
No. of reflections	7021
No. of parameters	368
No. of restraints	174
H-atom treatment	H-atom parameters constrained
$\Delta\rho_{max}$, $\Delta\rho_{min}$ (e Å^{-3})	0.33, -0.33

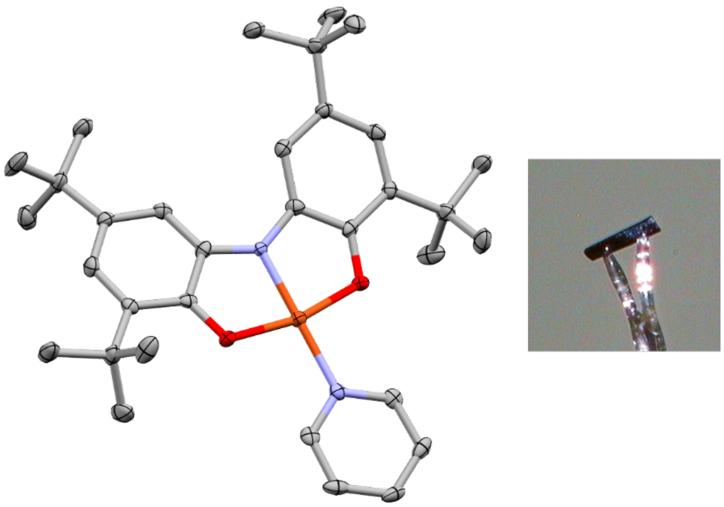

Figure S3. Displacement ellipsoid plot (50% probability level) of **(^{sq}ONO)Cu(py) (3)** at 110 K. Selected crystallographic bond lengths and bond angles are given in **Table S5**. A photograph of the single crystal used for analysis is shown on the right.

Crystallization: Crystals of **3** suitable for X-ray diffraction analysis were grown by slow evaporation of an Et_2O solution under anaerobic conditions.

Crystal Structure Solving: Reflection intensities were measured at 110(2) K using a SuperNova diffractometer (equipped with Atlas detector) with Cu *K*α radiation (λ = 1.54178 Å). The structure was solved with the program SHELXS-2018/3 (Sheldrick, 2018) and was refined on F^2 with SHELXL-2018/3 (Sheldrick, 2018). Empirical absorption correction using spherical harmonics was applied using the program CrysAlisPro. The H atoms were placed at calculated positions using the instructions AFIX 43 or AFIX 137 with isotropic displacement parameters having values 1.2 or 1.5 U_{eq} of the attached C atoms.

Structural Description: The structure is ordered. The crystal that was mounted on the diffractometer was found to be non-merohedrally twinned with two components, and the twin relationship corresponds to a twofold axis along the c direction. The BASF scale factor refines to 0.4807(17).

Table S5. Selected crystallographic bond lengths, angles, and geometry index for **(^{sq}ONO)Cu(py)** (**3**).

**Bond**	**Bond Length (Å)**	
	A ring	***B ring***
**C1–N**	1.373(4)	1.373(4)
**C2–O**	1.323(4)	1.323(4)
**C1–C2**	1.437(5)	1.437(5)
**C2–C3**	1.424(5)	1.424(5)
**C3–C4**	1.382(5)	1.382(5)
**C4–C5**	1.419(5)	1.419(5)
**C5–C6**	1.379(5)	1.379(5)
**C6–C1**	1.410(5)	1.410(5)
**Cu–O**	1.955(2)	1.955(2)
**Cu–N**	1.901(4)	
**Cu–L1**	1.971(4)	
**Bond Angle**	**Angle (°)**	
**O–Cu–O**	167.07(14)	
**N–Cu–L1**	180.00	
**O–Cu–N**	83.54(7)	
**O–Cu–N**	83.54(7)	
**O–Cu–L1**	96.46(7)	
**O–Cu–L1**	96.46(7)	
**Geometry Index**	**Index**	
**τ_4**	0.09	

Table S6. Crystallographic data for **(^{sq}ONO)Cu(py)** (**3**).

(^{sq}ONO)Cu(py) (3) Crystal data	
Chemical formula	$C_{33}H_{45}CuN_2O_2$
M_r	565.25
Crystal system, space group	Monoclinic, *I*2/*a*
Temperature (K)	110
a, *b*, *c* (Å)	6.5156 (8), 19.012 (2), 24.797 (3)
β (°)	90.713 (8)
V (Å^3)	3071.5 (6)
Z	4
Radiation type	Mo *K*α
μ (mm^{-1})	0.74
Crystal size (mm)	0.21 × 0.06 × 0.04
**Data collection**	
Diffractometer	SuperNova, Dual, Cu at zero, Atlas
Absorption correction	Multi-scan *CrysAlis PRO* 1.171.42.49 (Rigaku Oxford Diffraction, 2022) Empirical absorption correction using spherical harmonics, implemented in SCALE3 ABSPACK scaling algorithm.
T_{min}, T_{max}	0.540, 1.000
No. of measured, independent and observed [$I > 2\sigma(I)$] reflections	26274, 3266, 2445
R_{int}	0.144
$(\sin \theta/\lambda)_{max}$ (Å^{-1})	0.617
**Refinement**	
$R[F^2 > 2\sigma(F^2)]$, $wR(F^2)$, S	0.047, 0.104, 0.94
No. of reflections	3266
No. of parameters	181
H-atom treatment	H-atom parameters constrained
$\Delta\rho_{max}$, $\Delta\rho_{min}$ (e Å^{-3})	0.55, -0.40

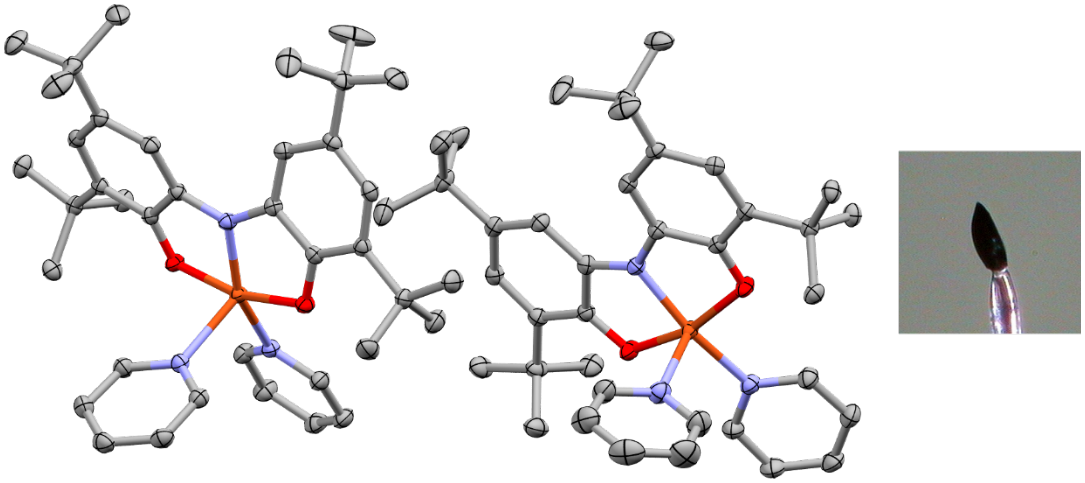

Figure S4. Displacement ellipsoid plots (50% probability level) of the two crystallographically independent Cu complexes of **(^{sq}ONO)Cu(py)$_2$ (4)** at 110 K. The lattice pyridine solvent molecule is omitted for clarity. Selected crystallographic bond lengths and bond angles are given in **Table S7**. *Note:* The left structure corresponds to “Complex 1”, and the right structure corresponds to “Complex 2” in **Table S7**. A photograph of the single crystal used for analysis is shown on the right.

Crystallization: Crystals of **4** suitable for X-ray diffraction analysis were obtained by cooling the pyridine filtrate from the synthesis of **3** (see **Scheme S1**) at -5 °C for >1 week.

Crystal Structure Solving: Reflection intensities were measured at 110 K using a SuperNova diffractometer (equipped with Atlas detector) with Cu *K*α radiation (λ = 1.54178 Å). The structure was solved with the program SHELXS-2018/3 (Sheldrick, 2018) and was refined on F^2 with SHELXL-2018/3 (Sheldrick, 2018). Analytical numeric absorption correction using a multifaceted crystal model was applied using the program CrysAlisPro. The H atoms were placed at calculated positions using the instructions AFIX 43 or AFIX 137 with isotropic displacement parameters having values 1.2 or 1.5 U_{eq} of the attached C atoms.

Structural Description: The asymmetric unit contains two crystallographically independent Cu complexes and one lattice pyridine solvent molecule. The structure is ordered.

Table S7. Selected crystallographic bond lengths, angles, and geometry indices for the two crystallographically independent structures of **(^{sq}ONO)Cu(py)$_2$** (**4**).

	Bond Length (Å) Complex 1		Bond Length (Å) Complex 2	
**Bond**	***A ring***	***B ring***	***A ring***	***B ring***
**C1–N**	1.365(2)	1.361(2)	1.364(2)	1.372(2)
**C2–O**	1.316(2)	1.318(2)	1.307(2)	1.317(2)
**C1–C2**	1.440(2)	1.442(2)	1.447(3)	1.443(2)
**C2–C3**	1.423(2)	1.422(2)	1.428(2)	1.425(2)
**C3–C4**	1.392(2)	1.395(2)	1.379(2)	1.387(2)
**C4–C5**	1.412(3)	1.410(2)	1.416(3)	1.416(2)
**C5–C6**	1.384(2)	1.384(2)	1.379(2)	1.380(3)
**C6–C1**	1.408(2)	1.405(2)	1.417(2)	1.410(2)
**Cu–O**	1.971(1)	1.979(1)	1.935(1)	1.943(1)
**Cu–N**	1.908(2)		1.932(1)	
**Cu–L1**	2.016(2)		1.998(1)	
**Cu–L2**	2.258(1)		2.283(2)	
**Bond Angle**	**Angle (°) Complex 1**		**Angle (°) Complex 2**	
**O–Cu–O**	165.03(5)		163.22(6)	
**N–Cu–L1**	156.23(6)		166.94(6)	
**N–Cu–L2**	107.88(6)		96.83(6)	
**O–Cu–N**	83.09(5)		83.78(6)	
**O–Cu–N**	82.98(5)		84.07(5)	
**O–Cu–L1**	94.21(5)		94.29(6)	
**O–Cu–L1**	96.32(5)		94.94(6)	
**O–Cu–L2**	94.07(5)		98.38(6)	
**O–Cu–L2**	95.40(5)		94.52(5)	
**L1–Cu–L2**	95.86(6)		96.23(6)	
**Geometry Index**	**Index Complex 1**		**Index Complex 2**	
**τ_5**	0.15		0.06	

Table S8. Crystallographic data for **(^{sq}ONO)Cu(py)$_2$** (**4**).

**(^{sq}ONO)Cu(py)$_2$ (4) Crystal data**	
Chemical formula	$2(C_{38}H_{50}CuN_3O_2)\cdot C_5H_5N$
M_r	1367.79
Crystal system, space group	Monoclinic, $P2_1/c$
Temperature (K)	110
a, *b*, *c* (Å)	33.8062 (4), 9.54196 (12), 24.5019 (3)
β (°)	108.7824 (13)
V (Å^3)	7482.87 (17)
Z	4
Radiation type	Cu $K\alpha$
μ (mm^{-1})	1.11
Crystal size (mm)	0.18 × 0.10 × 0.04
**Data collection**	
Diffractometer	SuperNova, Dual, Cu at zero, Atlas
Absorption correction	Analytical *CrysAlis PRO* 1.171.42.49 (Rigaku Oxford Diffraction, 2022) Analytical numeric absorption correction using a multifaceted crystal model based on expressions derived by R.C. Clark & J.S. Reid. (Clark, R. C. & Reid, J. S. (1995). Acta Cryst. A51, 887-897) Empirical absorption correction using spherical harmonics, implemented in SCALE3 ABSPACK scaling algorithm.
T_{min}, T_{max}	0.864, 0.960
No. of measured, independent and observed [$I > 2\sigma(I)$] reflections	60997, 14689, 12463
R_{int}	0.031
$(\sin \theta/\lambda)_{max}$ (Å^{-1})	0.617
**Refinement**	
$R[F^2 > 2\sigma(F^2)]$, $wR(F^2)$, S	0.033, 0.091, 1.02
No. of reflections	14689
No. of parameters	871
H-atom treatment	H-atom parameters constrained
$\Delta\rho_{max}$, $\Delta\rho_{min}$ (e Å^{-3})	0.29, -0.38

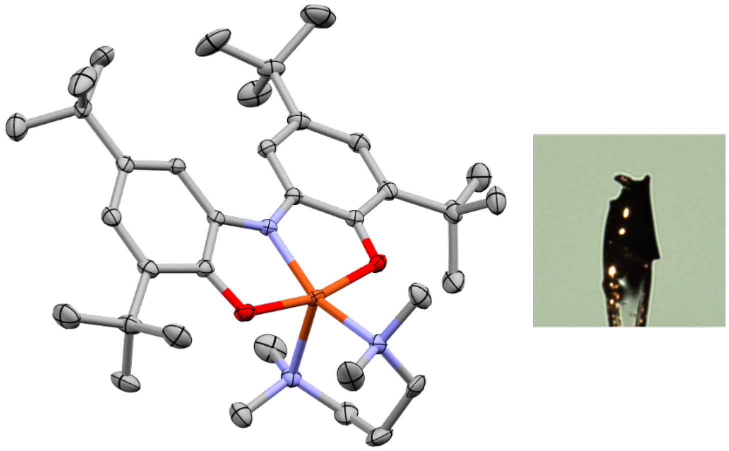

Figure S5. Displacement ellipsoid plot (50% probability level) of **(^{sq}ONO)Cu(tmpda) (5)** at 110 K. Disorder of the tmpda ligand was removed for clarity. Selected crystallographic bond lengths and bond angles are given in **Table S9**. A photograph of the single crystal used for analysis is shown on the right.

Crystallization: Crystals of the **5** suitable for X-ray diffraction analysis were grown by slow evaporation of an Et_2O solution under anaerobic conditions.

Crystal Structure Solving: Reflection intensities were measured at 110.00(10) K using a Rigaku XtaLAB Synergy R (equipped with a rotating-anode X-ray source and HyPix-6000HE detector) with Cu *K*α radiation (λ = 1.54178 Å). The structure was solved with the program SHELXS-2018/3 (Sheldrick, 2018) and was refined on F^2 with SHELXL-2018/3 (Sheldrick, 2018). Analytical numeric absorption correction using a multifaceted crystal model was applied using the program CrysAlisPro. The H atoms were placed at calculated positions using the instructions AFIX 23, AFIX 43 or AFIX 137 with isotropic displacement parameters having values 1.2 or 1.5 U_{eq} of the attached C atoms.

Structural Description: The structure is partly disordered. The tmpda ligand is disordered over two orientations, and the occupancy factor of the major component of the disorder refines to 0.672(3). The absolute configuration has been established by anomalous-dispersion effects in diffraction measurements on the crystal, and the Flack and Hooft parameters refine to -0.024(6) and -0.026(4), respectively.

Table S9. Selected crystallographic bond lengths, angles, and geometry index for **(^{sq}ONO)Cu(tmpda)** (**5**).

Bond	Bond Length (Å) A ring	Bond Length (Å) B ring
**C1–N**	1.375(3)	1.359(3)
**C2–O**	1.314(3)	1.308(3)
**C1–C2**	1.437(3)	1.445(3)
**C2–C3**	1.428(3)	1.432(3)
**C3–C4**	1.386(3)	1.384(3)
**C4–C5**	1.417(3)	1.416(3)
**C5–C6**	1.374(3)	1.371(3)
**C6–C1**	1.412(3)	1.418(3)
**Cu–O**	1.958(2)	1.961(2)
**Cu–N**	1.925(2)	
**Cu–L1**	2.053(7)	
**Cu–L2**	2.281(2)	
**Bond Angle**	**Angle (°)**	
**O–Cu–O**	166.42(7)	
**N–Cu–L1**	154.8(2)	
**N–Cu–L2**	107.56(7)	
**O–Cu–N**	83.71(7)	
**O–Cu–N**	83.50(7)	
**O–Cu–L1**	94.1(2)	
**O–Cu–L1**	95.5(2)	
**O–Cu–L2**	93.95(7)	
**O–Cu–L2**	94.26(7)	
**L1–Cu–L2**	97.6(2)	
**Geometry Index**	**Index**	
**τ_5**	0.19	

Table S10. Crystallographic data for **(^{sq}ONO)Cu(tmpda)** (**5**).

**(^{sq}ONO)Cu(tmpda) (5) Crystal data**	
Chemical formula	$C_{35}H_{58}CuN_3O_2$
M_r	616.38
Crystal system, space group	Orthorhombic, $P2_12_12_1$
Temperature (K)	110
a, *b*, *c* (Å)	12.43647 (12), 12.60505 (11), 22.3442 (2)
V (Å^3)	3502.73 (6)
Z	4
Radiation type	Cu *K*α
μ (mm^{-1})	1.12
Crystal size (mm)	0.21 × 0.13 × 0.03
**Data collection**	
Diffractometer	XtaLAB Synergy R, HyPix
Absorption correction	Analytical *CrysAlis PRO* 1.171.42.95a (Rigaku Oxford Diffraction, 2023) Analytical numeric absorption correction using a multifaceted crystal model based on expressions derived by R.C. Clark & J.S. Reid. (Clark, R. C. & Reid, J. S. (1995). Acta Cryst. A51, 887-897) Empirical absorption correction using spherical harmonics, implemented in SCALE3 ABSPACK scaling algorithm.
T_{min}, T_{max}	0.851, 0.971
No. of measured, independent and observed [$I > 2\sigma(I)$] reflections	36578, 6859, 6566
R_{int}	0.029
$(\sin \theta/\lambda)_{max}$ (Å^{-1})	0.616
**Refinement**	
$R[F^2 > 2\sigma(F^2)]$, $wR(F^2)$, S	0.027, 0.074, 1.07
No. of reflections	6859
No. of parameters	463
No. of restraints	301
H-atom treatment	H-atom parameters constrained
$\Delta\rho_{max}$, $\Delta\rho_{min}$ (e Å^{-3})	0.39, -0.36
Absolute structure	Flack x determined using 2818 quotients [(I+)-(I-)]/[(I+)+(I-)] (Parsons, Flack and Wagner, Acta Cryst. B69 (2013) 249-259).
Absolute structure parameter	-0.024 (6)

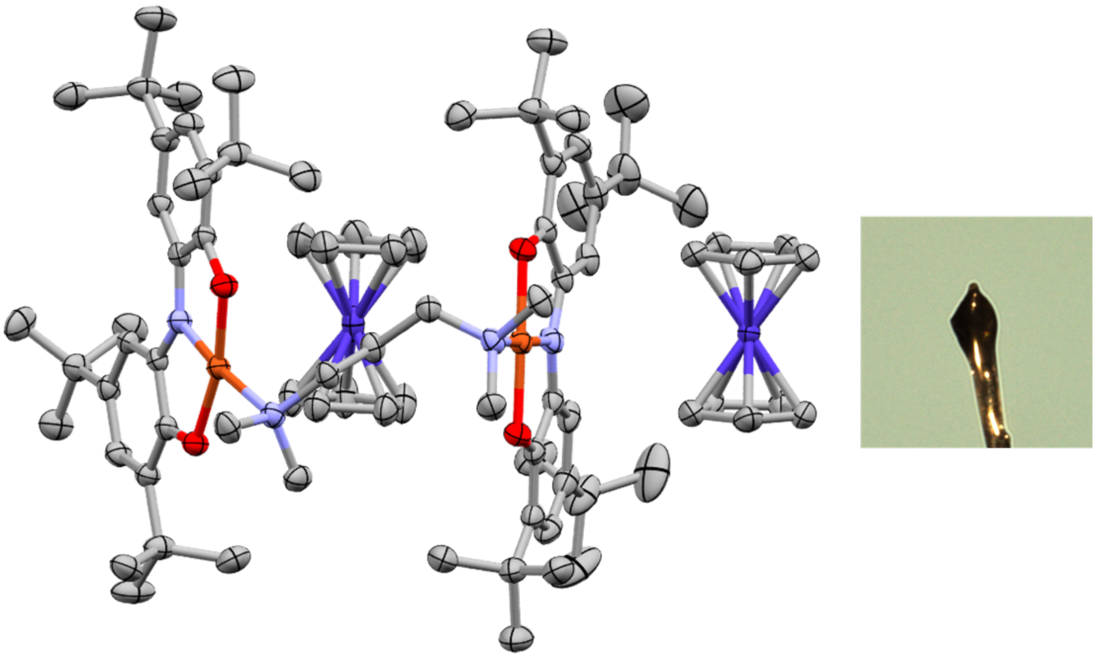

Figure S6. Displacement ellipsoid plot (50% probability level) of **$[\{(^{cat}ONO)Cu\}_2(\mu\text{-}tmpda)][CoCp_2]_2$ (5-cat)** at 110 K. Lattice solvent molecules (Et_2O, MeCN) and disorder of the ligand *tert*-butyl groups were removed for clarity. Selected crystallographic bond lengths and bond angles are given in **Table S11**. *Note:* The left Cu center corresponds to “Cu 1” and the right Cu center corresponds to “Cu 2” in **Table S11**. A photograph of the single crystal used for analysis is shown on the right.

Crystallization: Crystals of the **5-cat** suitable for X-ray diffraction analysis were obtained by layering Et_2O on an acetonitrile/toluene solution after storage at -35 °C for >3 d under anaerobic conditions.

Crystal Structure Solving: Reflection intensities were measured at 110.00(10) K using a Rigaku XtaLAB Synergy R (equipped with a rotating-anode X-ray source and HyPix-6000HE detector) with Cu *K*α radiation (λ = 1.54178 Å). The structure was solved with the program SHELXT-2018/2 (Sheldrick, 2018) and was refined on F^2 with SHELXL-2019/3 (Sheldrick, 2018). Analytical numeric absorption correction using a multifaceted crystal model was applied using the program CrysAlisPro. The H atoms were placed at calculated positions using the instructions AFIX 23, AFIX 43 or AFIX 137 with isotropic displacement parameters having values 1.2 or 1.5 U_{eq} of the attached C atoms.

Structural Description: The structure is partly disordered. Two tert-butyl groups are disordered over two orientations, and the occupancy factors of the major components of the disorder refine to 0.721(5) and 0.518(4). The asymmetric unit also contains two ordered lattice Et_2O and one ordered MeCN lattice solvent molecules. One site (found at one inversion center) most likely contains a disordered mixture of lattice Et_2O/MeCN solvent molecules, and that contribution was removed from the final refinement using the SQUEEZE procedure in Platon (Spek, 2009).

Table S11. Selected crystallographic bond lengths, angles, and geometry indices for **[{(^{cat}ONO)Cu}$_2$(μ-tmpda)][CoCp$_2$]$_2$** (**5-cat**).

	Bond Length (Å) Cu 1		Bond Length (Å) Cu 2	
**Bond**	***A ring***	***B ring***	***A ring***	***B ring***
**C1–N**	1.374(3)	1.384(3)	1.386(3)	1.377(3)
**C2–O**	1.342(3)	1.347(3)	1.352(2)	1.342(3)
**C1–C2**	1.438(3)	1.443(3)	1.431(3)	1.439(3)
**C2–C3**	1.399(3)	1.398(3)	1.405(3)	1.406(3)
**C3–C4**	1.410(3)	1.405(3)	1.410(3)	1.407(4)
**C4–C5**	1.392(3)	1.383(3)	1.387(4)	1.389(3)
**C5–C6**	1.404(3)	1.400(3)	1.399(3)	1.403(4)
**C6–C1**	1.396(3)	1.394(3)	1.400(3)	1.399(3)
**Cu–O**	1.915(1)	1.902(2)	1.908(1)	1.902(1)
**Cu–N**	1.881(2)		1.888(2)	
**Cu–L1**	2.052(2)		2.056(2)	
**Bond Angle**	**Angle (°) Complex 1**		**Angle (°) Complex 2**	
**O–Cu–O**	168.14(7)		170.05(7)	
**N–Cu–L1**	171.97(8)		176.21(8)	
**O–Cu–N**	85.40(7)		85.37(7)	
**O–Cu–N**	85.73(7)		85.57(7)	
**O–Cu–L1**	95.53(7)		92.89(7)	
**O–Cu–L1**	94.35(7)		96.43(7)	
**Geometry Index**	**Index Complex 1**		**Index Complex 2**	
**τ_4**	0.14		0.10	

Table S12. Crystallographic data for **[{(^{cat}ONO)Cu}$_2$(μ-tmpda)][CoCp$_2$]$_2$** (**5-cat**).

[{(^{cat}ONO)Cu}$_2$(μ-tmpda)][CoCp$_2$]$_2$ (5-cat) Crystal Data	
Chemical formula	$C_{63}H_{98}Cu_2N_4O_4 \cdot 2(C_{10}H_{10}Co) \cdot 2(C_4H_{10}O) \cdot C_2H_3N$
M_r	1670.04
Crystal system, space group	Triclinic, *P*-1
Temperature (K)	110
a, *b*, *c* (Å)	14.7292 (5), 16.1018 (4), 21.4109 (6)
α, β, γ (°)	70.025 (2), 83.828 (3), 88.017 (2)
V (Å^3)	4744.8 (2)
Z	2
Radiation type	Cu $K\alpha$
μ (mm^{-1})	3.58
Crystal size (mm)	0.15 × 0.11 × 0.03
**Data collection**	
Diffractometer	XtaLAB Synergy R, HyPix
Absorption correction	Analytical *CrysAlis PRO* 1.171.43.90 (Rigaku Oxford Diffraction, 2023) Analytical numeric absorption correction using a multifaceted crystal model based on expressions derived by R.C. Clark & J.S. Reid. (Clark, R. C. & Reid, J. S. (1995). Acta Cryst. A51, 887-897) Empirical absorption correction using spherical harmonics, implemented in SCALE3 ABSPACK scaling algorithm.
T_{min}, T_{max}	0.678, 0.897
No. of measured, independent and observed [$I > 2\sigma(I)$] reflections	110228, 18587, 15569
R_{int}	0.050
$(\sin \theta/\lambda)_{max}$ (Å^{-1})	0.617
**Refinement**	
$R[F^2 > 2\sigma(F^2)]$, $wR(F^2)$, S	0.042, 0.114, 1.04
No. of reflections	18587
No. of parameters	1068
No. of restraints	240
H-atom treatment	H-atom parameters constrained
$\Delta\rho_{max}$, $\Delta\rho_{min}$ (e Å^{-3})	0.66, -0.54

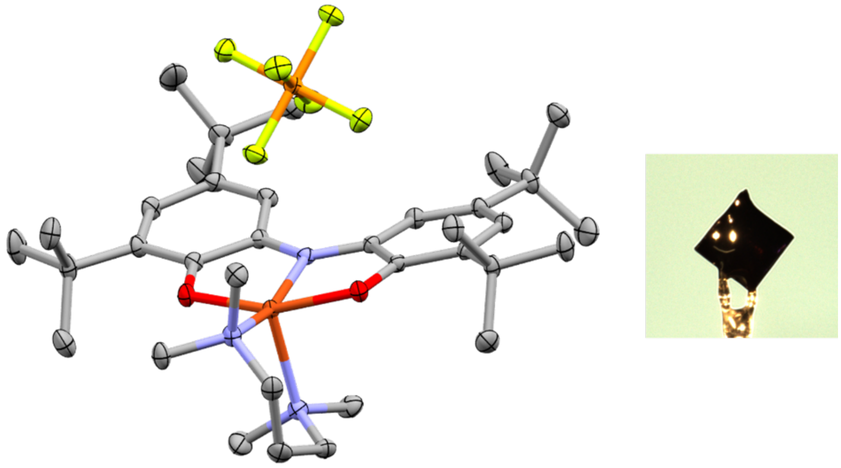

Figure S7. Displacement ellipsoid plots (50% probability level) of **[(^{bq}ONO)Cu(tmpda)]PF_6 (5-bq)** at 110 K. Selected crystallographic bond lengths and bond angles are given in **Table S13**. A photograph of the single crystal used for analysis is shown on the right.

Crystallization: Crystals of **5-bq** suitable for X-ray diffraction analysis were obtained layering hexane onto a toluene solution of the complex in an NMR tube under anaerobic conditions.

Crystal Structure Solving: Reflection intensities were measured at 110.00(10) K using a Rigaku XtaLAB Synergy R (equipped with a rotating-anode X-ray source and HyPix-6000HE detector) with Cu *K*α radiation (λ = 1.54178 Å). The structure was solved with the program SHELXT-2018/2 (Sheldrick, 2018) and was refined on F^2 with SHELXL-2019/3 (Sheldrick, 2018). Analytical numeric absorption correction using a multifaceted crystal model was applied using the program CrysAlisPro. The H atoms were placed at calculated positions using the instructions AFIX 23, AFIX 43 or AFIX 137 with isotropic displacement parameters having values 1.2 or 1.5 U_{eq} of the attached C atoms.

Structural Description: The structure is ordered.

Table S13. Selected crystallographic bond lengths, angles, and geometry index for **[(^{bq}ONO)Cu(tmpda)]PF$_6$** (**5-bq**).

Bond	Bond Length (Å)	
	A ring	*B ring*
**C1–N**	1.341(2)	1.351(2)
**C2–O**	1.274(1)	1.282(2)
**C1–C2**	1.468(2)	1.455(2)
**C2–C3**	1.447(2)	1.448(2)
**C3–C4**	1.366(2)	1.369(2)
**C4–C5**	1.448(2)	1.437(2)
**C5–C6**	1.360(2)	1.360(2)
**C6–C1**	1.425(2)	1.422(2)
**Cu–O**	1.9795(9)	1.9548(9)
**Cu–N**	1.961(1)	
**Cu–L1**	2.012(1)	
**Cu–L2**	2.220(1)	
**Bond Angle**	**Angle (°)**	
**O–Cu–O**	159.96(4)	
**N–Cu–L1**	161.60(4)	
**N–Cu–L2**	99.18(4)	
**O–Cu–N**	82.12(4)	
**O–Cu–N**	83.32(4)	
**O–Cu–L1**	93.12(4)	
**O–Cu–L1**	96.40(4)	
**O–Cu–L2**	94.50(4)	
**O–Cu–L2**	101.37(4)	
**L1–Cu–L2**	98.90(4)	
**Geometry Index**	**Index**	
**τ_5**	0.03	

Table S14. Crystallographic data for **[(^{bq}ONO)Cu(tmpda)]PF_6 (5-bq)**.

[(^{bq}ONO)Cu(tmpda)]PF_6 (5-bq) Crystal Data	
Chemical formula	$C_{35}H_{58}CuN_3O_2·F_6P$
M_r	761.35
Crystal system, space group	Monoclinic, $P2_1/n$
Temperature (K)	110
a, *b*, *c* (Å)	14.56792 (8), 13.91108 (7), 19.72319 (11)
β (°)	100.8204 (5)
V (Å^3)	3925.95 (4)
Z	4
Radiation type	Cu $K\alpha$
μ (mm^{-1})	1.70
Crystal size (mm)	0.36 × 0.32 × 0.04
**Data collection**	
Diffractometer	XtaLAB Synergy R, HyPix
Absorption correction	Analytical *CrysAlis PRO* 1.171.43.90 (Rigaku Oxford Diffraction, 2023) Analytical numeric absorption correction using a multifaceted crystal model based on expressions derived by R.C. Clark & J.S. Reid. (Clark, R. C. & Reid, J. S. (1995). Acta Cryst. A51, 887-897) Empirical absorption correction using spherical harmonics, implemented in SCALE3 ABSPACK scaling algorithm.
T_{min}, T_{max}	0.631, 0.943
No. of measured, independent and observed [$I > 2\sigma(I)$] reflections	80035, 7693, 7307
R_{int}	0.029
$(\sin \theta/\lambda)_{max}$ (Å^{-1})	0.616
**Refinement**	
$R[F^2 > 2\sigma(F^2)]$, $wR(F^2)$, S	0.026, 0.069, 1.05
No. of reflections	7693
No. of parameters	450
H-atom treatment	H-atom parameters constrained
$\Delta\rho_{max}$, $\Delta\rho_{min}$ (e Å^{-3})	0.39, -0.38

3. UV-vis Spectra

3.1 UV-vis Oxidation and Reduction of Cu-iminosemiquinone Complexes

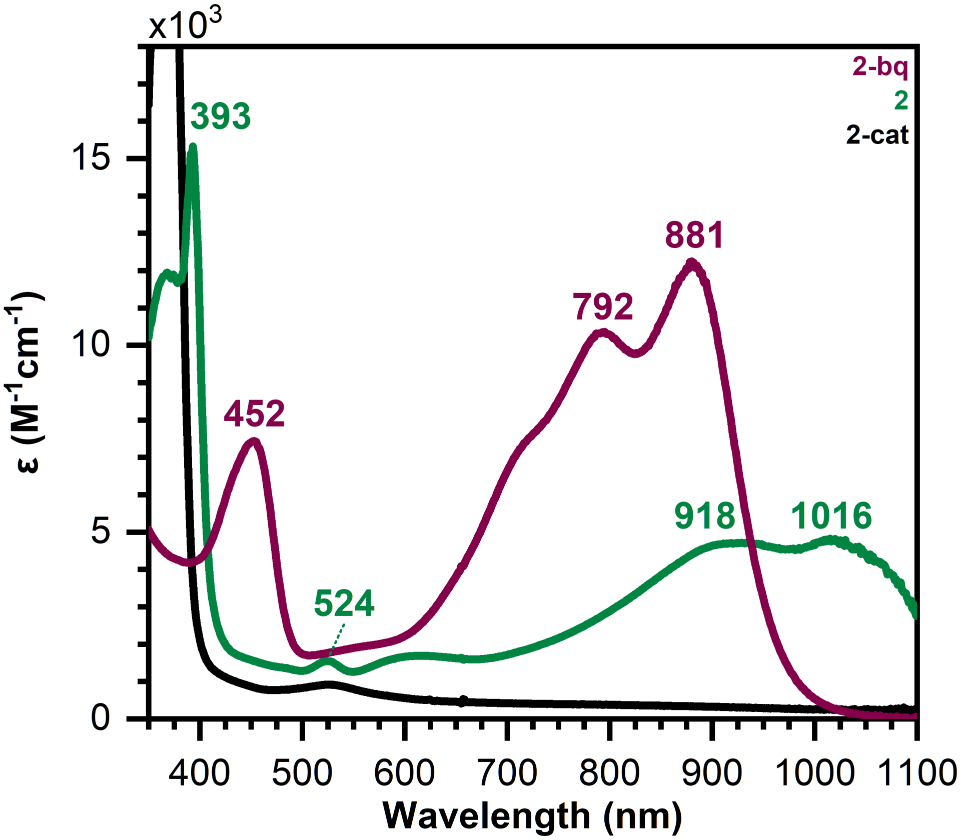

Figure S8. UV-vis spectra of **(^{sq}ONO)Cu(NMI) (2)** and its 1-electron oxidized (**2-bq**, maroon trace) and reduced (**2-cat**, black trace) forms. Oxidation and reduction of **2** was achieved by the addition of 1 equiv. of $FcPF_6$ or $CoCp_2$, respectively.

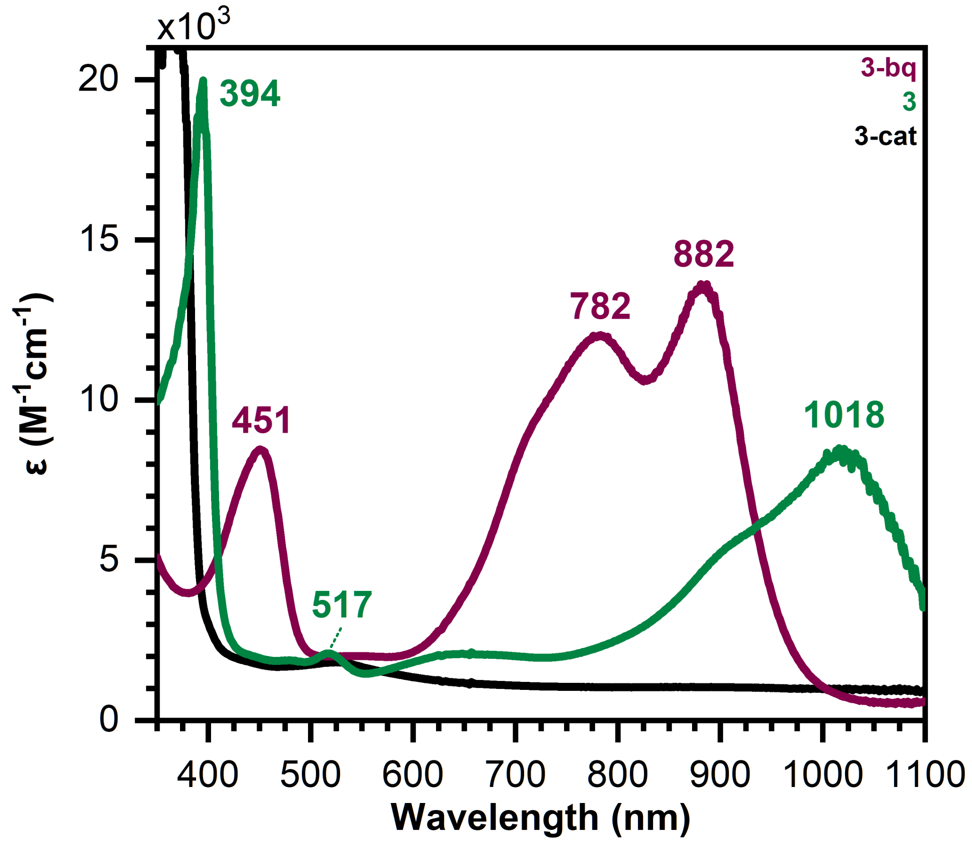

Figure S9. UV-vis spectra of **(^sqONO)Cu(py) (3)** and its 1-electron oxidized (**3-bq**, maroon trace) and reduced (**3-cat**, black trace) forms. Oxidation and reduction of **3** was achieved by the addition of 1 equiv. of $FcPF_6$ or $CoCp_2$, respectively.

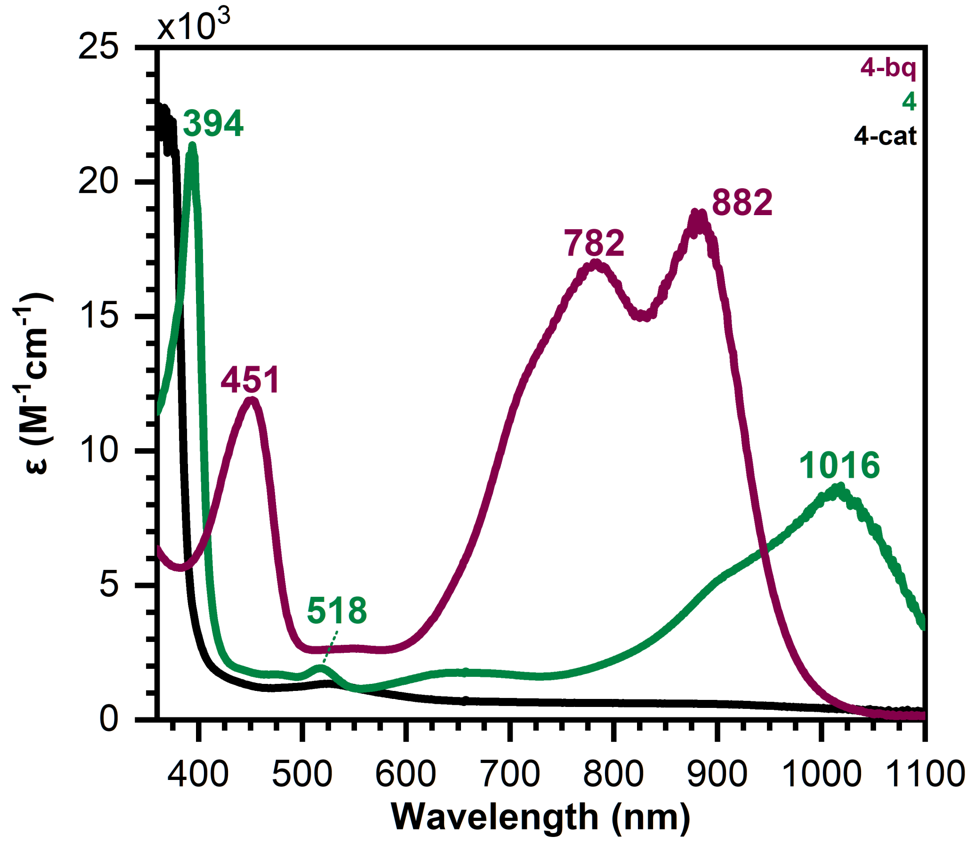

Figure S10. UV-vis spectra of **(^{sq}ONO)Cu(py)$_2$ (4)** and its 1-electron oxidized (**4-bq**, maroon trace) and reduced (**4-cat**, black trace) forms. Oxidation and reduction of **4** was achieved by the addition of 1 equiv. of $FcPF_6$ or $CoCp_2$, respectively.

3.2 UV-vis Absorption Data

Table S15. The UV-vis absorption data for the Cu-iminosemiquinone complexes **1–5** in DMF. The λ_{max} (nm) and molar absorption coefficients (ε, $M^{-1}cm^{-1}$; in parenthesis) of the main absorption features for each complex are given. *Note:* The molar absorption coefficients are based on the average of at least three independent experiments.

	λ_{max} [nm] (ε [$M^{-1}cm^{-1}$])			
**Complex**	λ^1	λ^2	λ^3	λ^4
**(^{sq}ONO)Cu(NEt_3) (1)**	394 (18960)	517 (1670)		1018 (7540)
**(^{sq}ONO)Cu(NMI) (2)**	393 (15400)	524 (1800)	918 (4990)	1016 (4960)
**(^{sq}ONO)Cu(py) (3)**	394 (20000)	517 (1730)		1018 (8160)
**(^{sq}ONO)Cu$(py)_2$ (4)**	394 (21000)	518 (2010)		1016 (9330)
**(^{sq}ONO)Cu(tmpda) (5)**	397 (20200)	510 (1570)		993 (9510)

Table S16. The UV-vis absorption data for the oxidized complexes **1-bq**–**5-bq** in DMF. The λ_{max} (nm) and molar absorption coefficients (ε, $M^{-1}cm^{-1}$; in parenthesis) of the main absorption features for each complex are given. *Note:* The molar absorption coefficients are based on the average of at least three independent experiments.

	λ_{max} [nm] (ε [$M^{-1}cm^{-1}$])		
**Complex**[a]	λ^1	λ^2	λ^4
**1-bq**	451 (8450)	781 (11850)	882 (12900)
**2-bq**	452 (7700)	792 (10250)	881 (12090)
**3-bq**	451 (8460)	782 (11890)	882 (13520)
**4-bq**[b]	451 (11900)	782 (17010)	882 (18490)
**5-bq**	455 (8800)	787 (11060)	875 (12200)

[a] The oxidized complexes were generated by addition of 1 equiv. of $FcPF_6$ to the corresponding Cu-iminosemiquinone complex. [b] The molar absorptivity for **4-bq** was based on only two measurements and may have a higher associated uncertainty.

4. EPR Spectroscopy

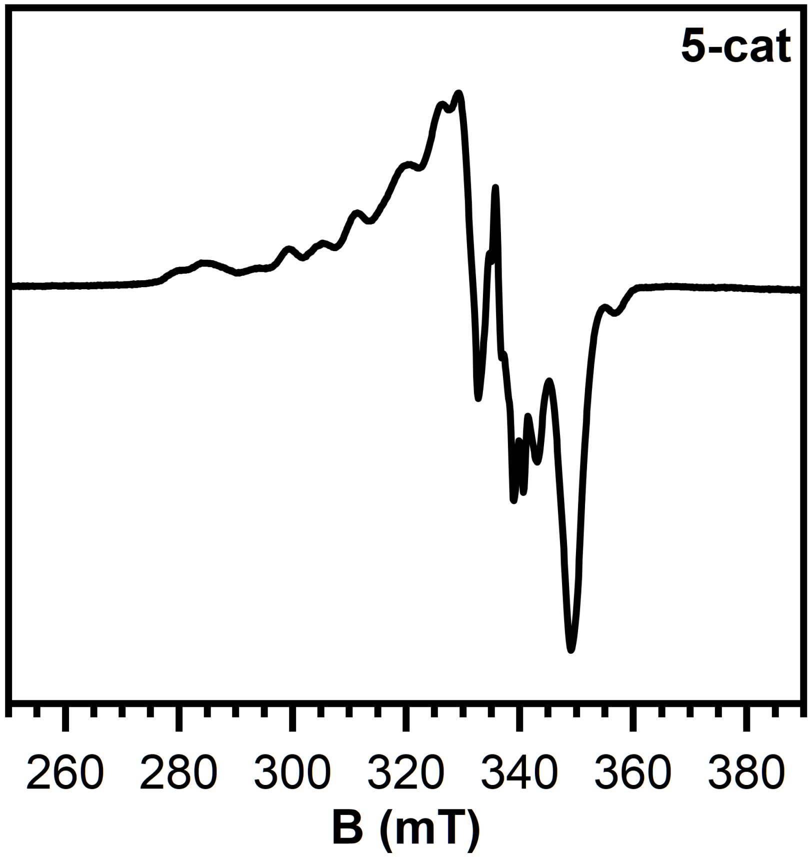

Figure S11. X-band EPR spectrum of **5-cat** (generated by addition of 1 equiv. of $CoCp_2$ to complex **5**) in a frozen 1:1 (v/v) DMF/toluene solution at 19 K. The presence of multiple distinct Cu^{II} species is evident by the observation of multiple sets of Cu hyperfine splittings.

5. Evans Method Magnetic Susceptibility

The Evans method was used to measure the bulk magnetic susceptibility of the complexes in solution. The molar magnetic susceptibility of the analyte was calculated using equation (1):[2]

$$\chi_m = 477 \frac{\Delta f}{2c\nu_0} \tag{1}$$

Where X_m is the molar magnetic susceptibility of the analyte in cm^3mol^{-1}, *c* is the molar concentration of the analyte in $molL^{-1}$, Δf is the peak separation between the internal standard resonances in Hz, and ν_0 is the frequency of the NMR spectrometer in Hz.

Diamagnetic corrections were applied using Pascal's constants to estimate the paramagnetic contribution (X_P) to the molar magnetic susceptibility.[3] The effective magnetic moment (μ_{eff}, in μ_B) of the analyte in solution at temperature *T* (in K) was calculated using equation (2):[4]

$$\mu_{\mathrm{eff}} = \sqrt{8\chi_P T} \tag{2}$$

Table S17. Effective magnetic moment (μ_{eff}) for the Cu-iminosemiquinone complexes as determined by Evans Method in C_6D_6 at 298 K (500 MHz NMR spectrometer).

Complex	μ_{eff} [μ_B]
**(^{sq}ONO)Cu(NEt$_3$) (1)**	2.09
**(^{sq}ONO)Cu(NMI) (2)**	2.28
**(^{sq}ONO)Cu(py) (3)**	2.52
**(^{sq}ONO)Cu(tmpda) (5)**	2.95

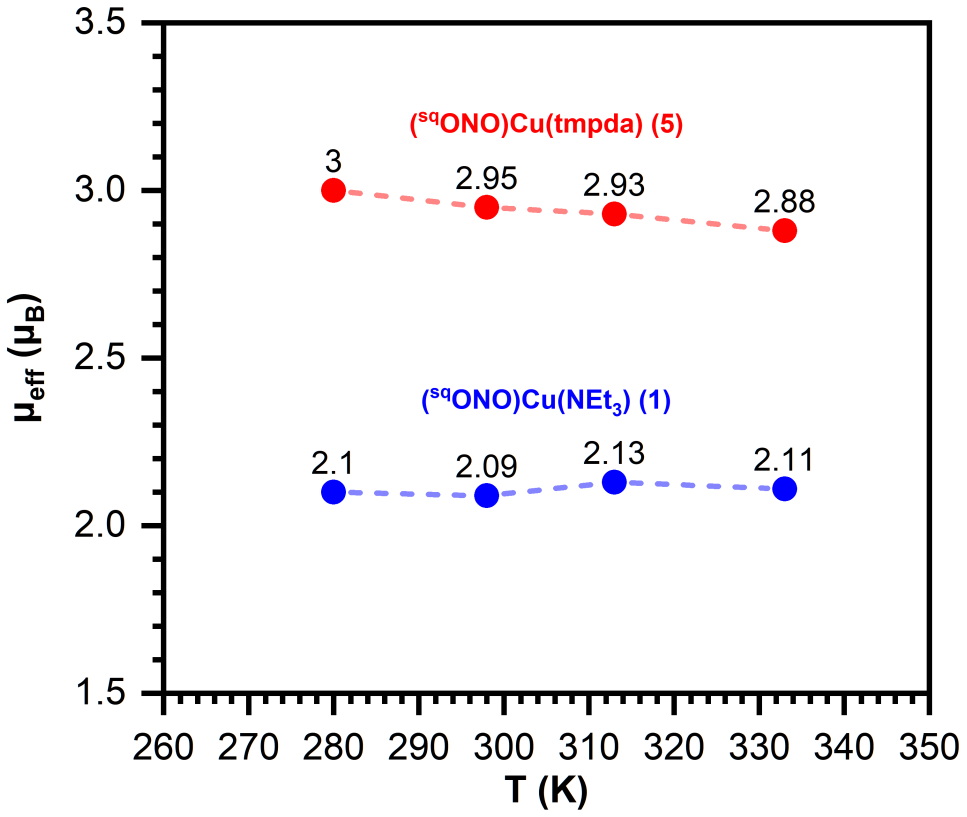

Figure S12. Variable-temperature Evans method magnetic susceptibility measurements for complexes **1** and **5** in C_6D_6 (500 MHz NMR spectrometer).

6. DFT Calculations

All DFT calculations were performed with the Amsterdam Density Functional (ADF)[5,6] and QUILD[7] programs, and were performed using the unrestricted Kohn-Sham scheme. Molecular orbitals were expanded in an uncontracted set of Slater type orbitals (STOs) of triple-ζ quality with double polarization functions (TZ2P).[8,9] Core electrons were not treated explicitly during the geometry optimizations (frozen core approximation[6]). An auxiliary set of s, p, d, f, and g STOs was used to fit the molecular density and to represent the coulomb and exchange potentials accurately for each SCF cycle.

Geometries of all possible spin states were optimized with the QUILD[7] program using adapted delocalized coordinates until the maximum gradient component was less than 10^{-4} a.u. Energies, gradients and Hessians[10] (for vibrational frequencies) were calculated using S12g[11], in all cases by including solvation effects through the COSMO[12] dielectric continuum model with appropriate parameters for the solvents.[13] For computing Gibbs free energies, all small frequencies were raised to 100 cm^{-1} in order to compensate for the breakdown of the harmonic oscillator model.[14,15] Scalar relativistic corrections have been included self-consistently in all calculations by using the zeroth-order regular approximation (ZORA[16]). All calculations have been performed with a Becke grid of VeryGood quality, except the vibrational frequencies for which a Normal grid was used.

All computational data will be uploaded onto the IOCHEM-BD platform (www.iochem-bd.org[17]) to facilitate data exchange and dissemination, according to the FAIR principles[18] of OpenData sharing.

6.1 Spin Density Plots

[(^{cat}ONO)Cu(NEt$_3$)]$^-$ (1-cat)

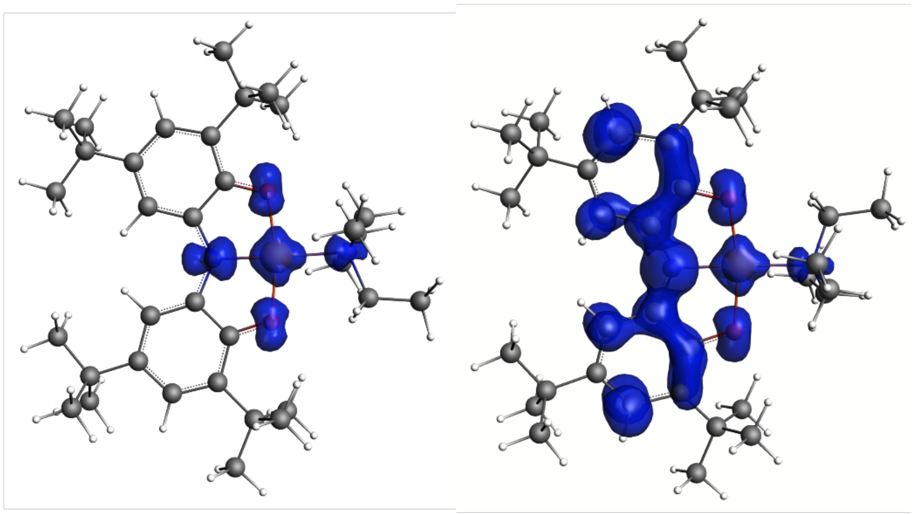

1-cat^D
S = 1/2

1-cat^Q
S = 3/2

Figure S13. Spin density plots for **1-cat** in doublet (**1-cat^D**) and quadruplet (**1-cat^Q**) spin states.

[(^{bq}ONO)Cu(NEt_3)]$^+$ (1-bq)

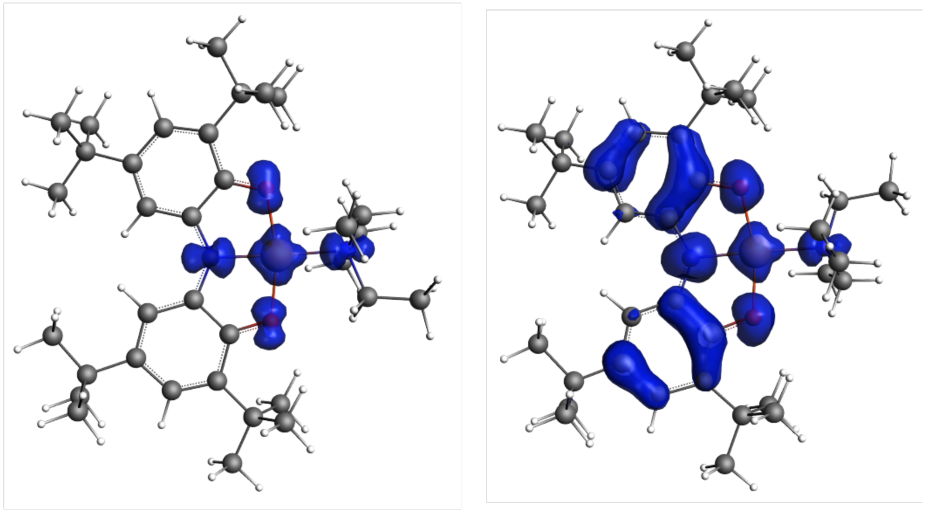

1-bq^D
S = 1/2

1-bq^Q
S = 3/2

Figure S14. Spin density plots for **1-bq** in doublet (**1-bq^D**) and quadruplet (**1-bq^Q**) spin states.

(^{sq}ONO)Cu(NEt$_3$) (1)

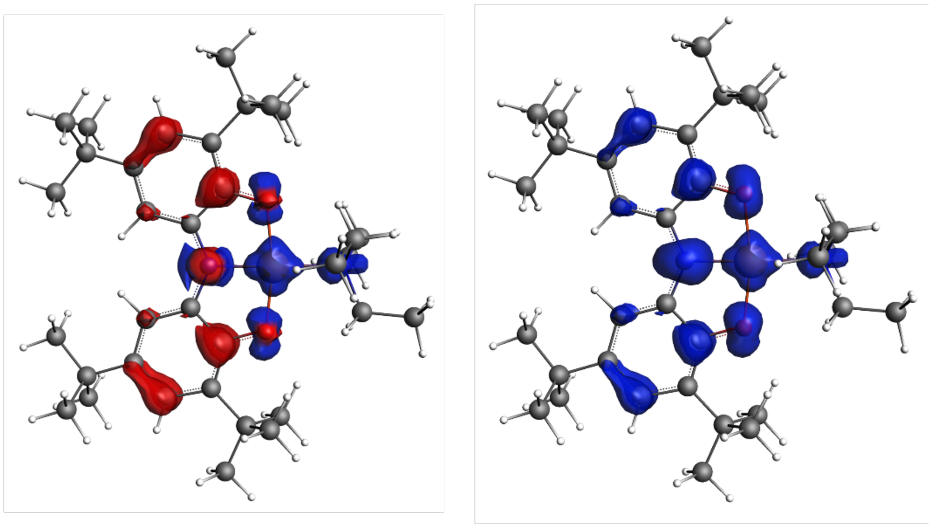

1^S
S = 0

1^T
S = 1

Figure S15. Spin density plots for **1** in open-shell singlet (**1^S**) and triplet (**1^T**) spin states. Both structures were optimized with C_S symmetry enforced.

(^{sq}ONO)Cu(NMI) (2)

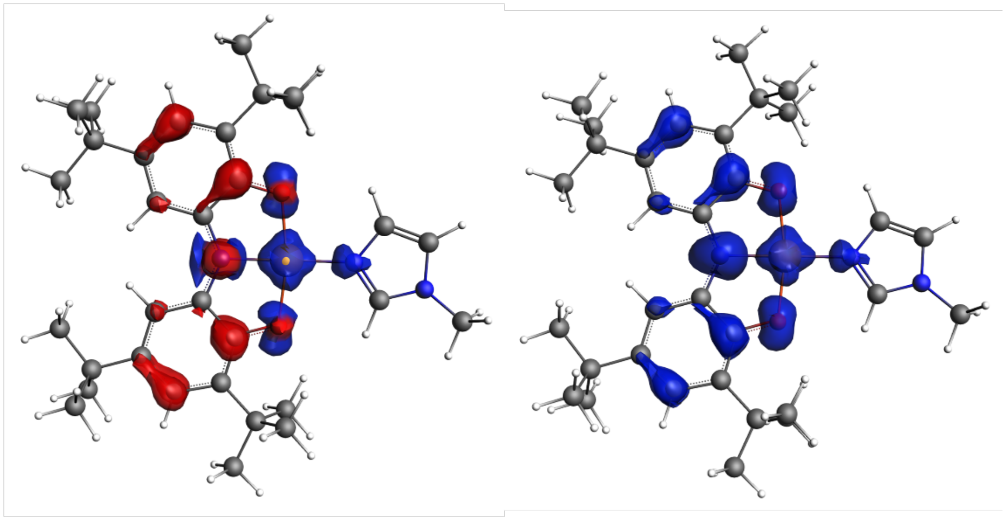

2^{S}
S = 0

2^{T}
S = 1

Figure S16. Spin density plots for **2** in open-shell singlet (**2^{S}**) and triplet (**2^{T}**) spin states. Both structures were optimized with C_S symmetry enforced.

(^{sq}ONO)Cu(py) (3)

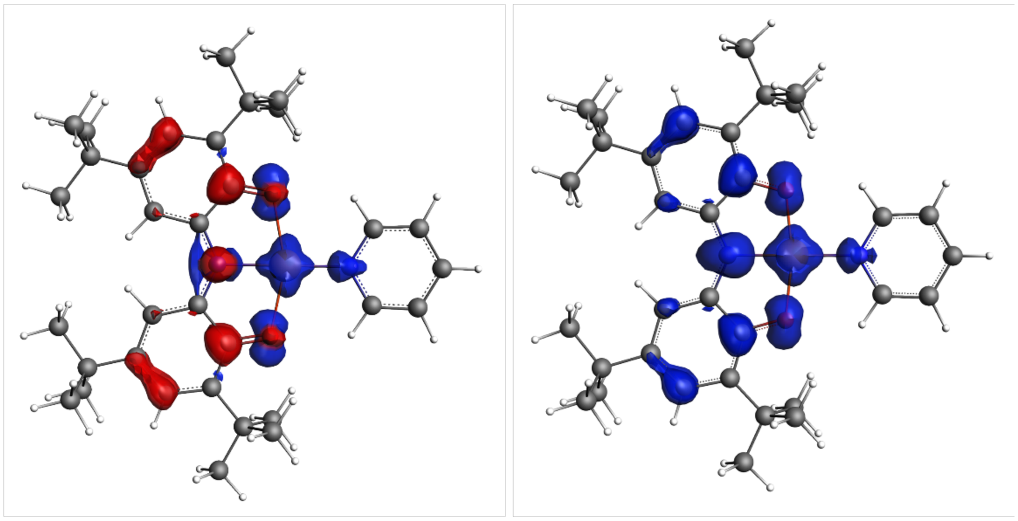

3^{S}
S = 0

3^{T}
S = 1

Figure S17. Spin density plots for **3** in open-shell singlet (**3^{S}**) and triplet (**3^{T}**) spin states. Both structures were optimized with C_{2v} symmetry enforced.

(sqONO)Cu(py)$_2$ (4)

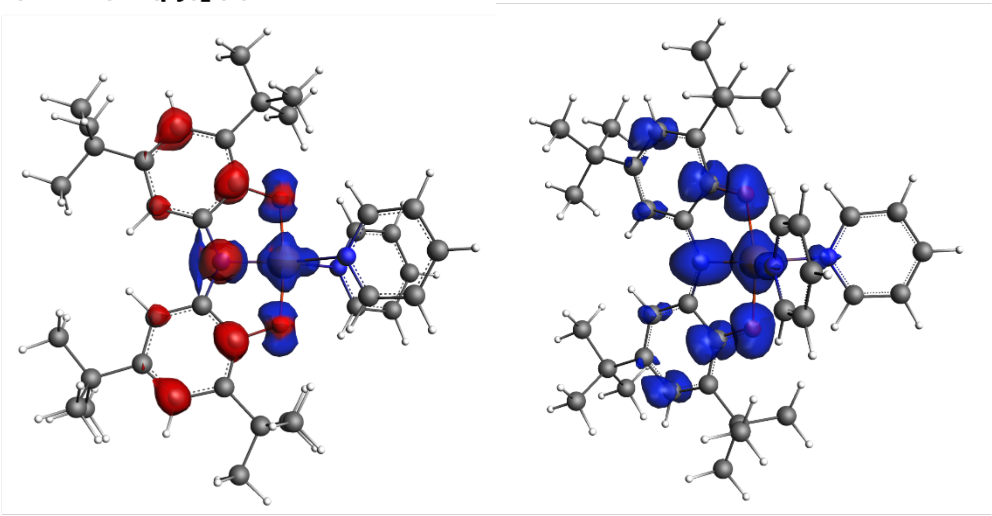

4^S
S = 0

4^T
S = 1

Figure S18. Spin density plots for **4** in open-shell singlet (**4^S**) and triplet (**4^T**) spin states. Both structures were optimized with C_{2v} symmetry enforced.

(^{sq}ONO)Cu(tmpda) (5)

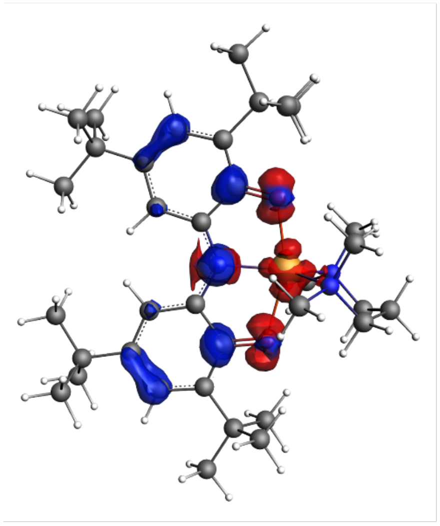

5^{S}
S = 0

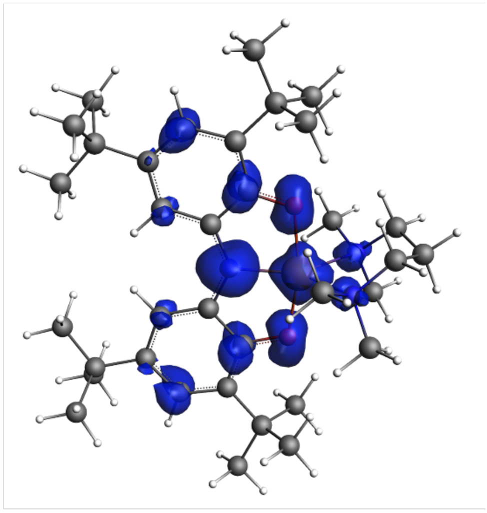

5^{T}
S = 1

Figure S19. Spin density plots for **5** in open-shell singlet (**5^{S}**) and triplet (**5^{T}**) spin states. Both structures were optimized with C_S symmetry enforced.

6.2 Spin States, Electronic Structures, and Structural Parameters

Table S18. Relative energies (E^{rel}, kcal·mol^{-1}) and structurally derived parameters (ligand metrical oxidation state and geometry indices) for the DFT-optimized Cu complexes in different spin states. Rows in bold indicate the calculated ground state electronic configuration.

Complex	Structure	S^*	E^{rel} (kcal·mol^{-1})	Ligand MOS†	Geometry Index‡ (τ)
**[(^{cat}ONO)Cu(NEt_3)]$^-$ (1-cat)**	**1-cat^D**	**1/2**	**0.0**	**-2.2**	**0.16**
	1-cat^Q	3/2	57.2	-1.9	0.10
**[(^{bq}ONO)Cu(NEt_3)]$^+$ (1-bq)**	**1-bq^D**	**1/2**	**0.0**	**-1.2**	**0.12**
	1-bq^Q	3/2	13.5	-1.6	0.12
**(^{sq}ONO)Cu(NEt_3) (1)**	1^S	0	1.05	-1.8	0.10
	**1^T**	**1**	**0.0**	**-1.8**	**0.10**
**(^{sq}ONO)Cu(NMI) (2)**	2^S	0	1.04	-1.8	0.08
	**2^T**	**1**	**0.0**	**-1.8**	**0.09**
**(^{sq}ONO)Cu(py) (3)**	3^S	0	1.07	-0.8	0.15
	**3^T**	**1**	**0.0**	**-1.8**	**0.08**
**(^{sq}ONO)Cu($py)_2$ (4)**	4^S	0	1.69	-2.7	0.56
	**4^T**	**1**	**0.0**	**-1.8**	**0.52**
**(^{sq}ONO)Cu(tmpda) (5)**	5^S	0	1.80	-1.1	0.46
	**5^T**	**1**	**0.0**	**-1.8**	**0.35**

* Open-shell singlet (S, S = 0); doublet (D, S = 1/2); triplet (T, S = 1); quadruplet (Q, S = 3/2). † Ligand metrical oxidation state (MOS). ‡ τ_4 geometry indices are given for **1-cat**, **1-bq**, and **1–3**; τ_5 geometry indices are given for **4** and **5**.

Table S19. Calculated d-orbital occupancies for **1–5** in open-shell singlet (*S* = 0, $\mathbf{1^S}$–$\mathbf{5^S}$) and triplet (*S* = 1, $\mathbf{1^T}$–$\mathbf{5^T}$) spin states.

Complex	e^-	Open-shell singlet (*S* = 0)	Triplet (*S* = 1)
**(^{sq}ONO)Cu(NEt$_3$) (1)**	α	0.960, 0.987, 0.997, 1.004, 1.012	0.960, 0.996, 1.002, 1.004, 1.012
	β	**0.417**, 0.954, 0.985, 1.002, 1.004	**0.423**, 0.955, 0.981, 0.985, 1.004
**(^{sq}ONO)Cu(NMI) (2)**	α	0.958, 0.984, 0.998, 1.001, 1.004	0.957, 0.993, 0.999, 1.003, 1.009
	β	**0.587**, 0.792, 0.952, 0.998, 1.003	**0.408**, 0.953, 0.975, 0.978, 1.003
**(^{sq}ONO)Cu(py) (3)**	α	0.979, 0.989, 0.996, 0.998, 1.003	0.977, 0.993, 0.994, 0.996, 1.003
	β	**0.544**, 0.861, 0.993, 0.997, 1.003	**0.548**, 0.806, 0.974, 0.995, 1.003
**(^{sq}ONO)Cu(py)$_2$ (4)**	α	0.920, 0.977, 0.988, 0.992, 1.001	0.991, 0.994, 0.995, 1.001, 1.005
	β	**0.632**, 0.717, 0.993, 1.001, 1.007	**0.657**, 0.713, 0.930, 0.997, 1.002
**(^{sq}ONO)Cu(tmpda) (5)**	α	**0.487**, 0.949, 0.974, 1.002, 1.005	0.982, 0.997, 1.003, 1.007, 1.008
	β	0.966, 0.993, 0.999, 1.000, 1.007	**0.448**, 0.924, 0.954, 0.979, 1.002

Note: Bolded d-orbital occupancy values (occupancy < 0.7) indicate partially filled d-orbitals.[19]

Table S20. Calculated d-orbital occupancies for **1-cat** and **1-bq** in the doublet (S = 1/2) ground state.

**Complex**	**e⁻**	**Doublet (S = 1/2)**
**[(^{cat}ONO)Cu(NEt_3)]$^-$ (1-cat^D)**	α	0.954, 0.995, 1.000, 1.003, 1.011
	β	**0.433**, 0.948, 0.994, 0.999, 1.003
**[(^{bq}ONO)Cu(NEt_3)]$^+$ (1-bq^D)**	α	0.966, 0.991, 0.998, 1.004, 1.012
	β	**0.431**, 0.962, 0.987, 0.987, 1.002

Note: Bolded d-orbital occupancy values (occupancy < 0.7) indicate partially filled d-orbitals.[19]

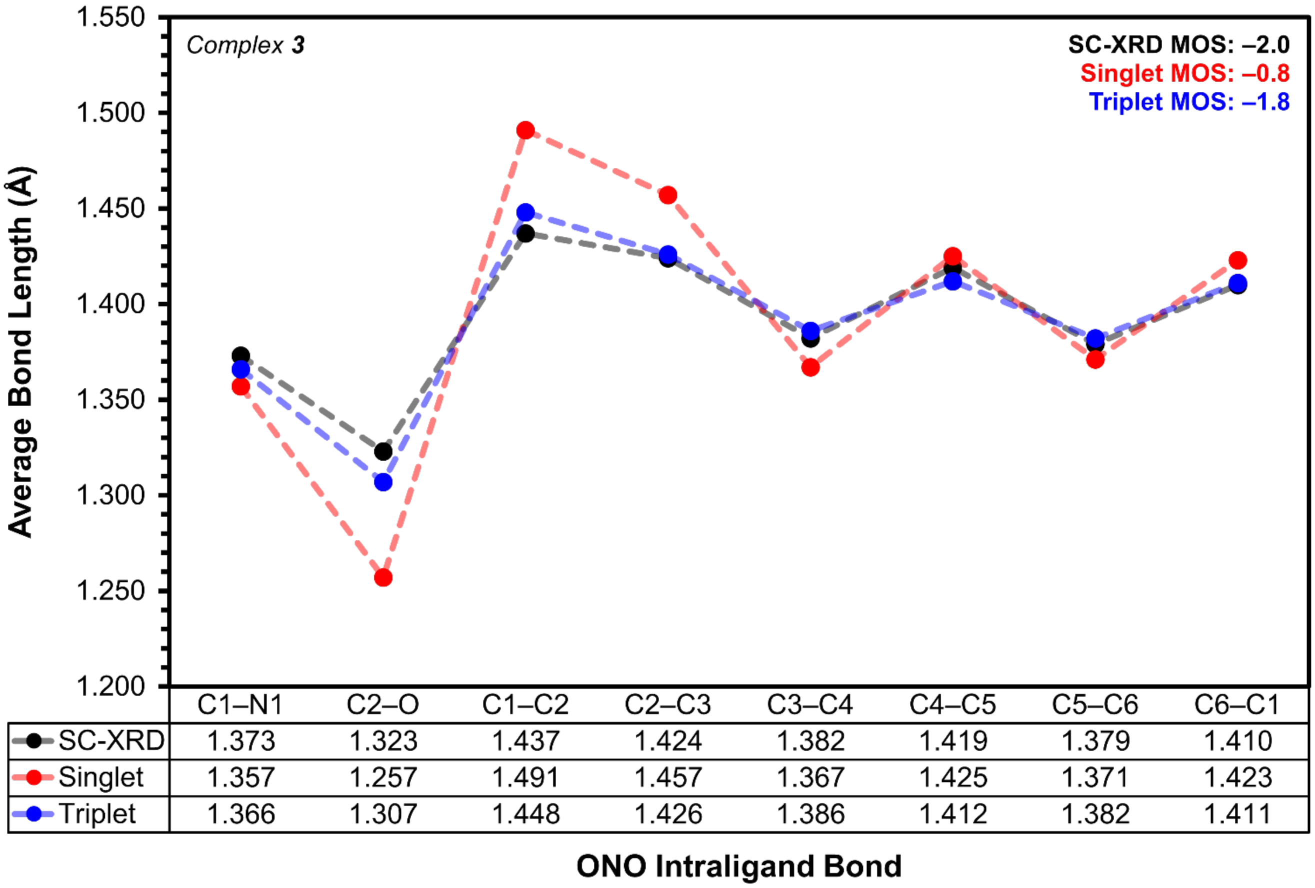

	C1–N1	C2–O	C1–C2	C2–C3	C3–C4	C4–C5	C5–C6	C6–C1
SC-XRD	1.373	1.323	1.437	1.424	1.382	1.419	1.379	1.410
Singlet	1.357	1.257	1.491	1.457	1.367	1.425	1.371	1.423
Triplet	1.366	1.307	1.448	1.426	1.386	1.412	1.382	1.411

Figure S20. Comparison of the average ONO intraligand bond lengths of the DFT-optimized structures of complex **3** in the open-shell singlet (S = 0, in red) and triplet (S = 1, in blue) spin states with the structure obtained experimentally by SC-XRD (in black). Average bond lengths (in angstroms) were derived from the chemically equivalent bonds in the ONO ligand.

The MOS value of the ONO ligand in the singlet structure of **3** ($\mathbf{3^S}$, MOS = −0.8) is significantly greater than those of the SC-XRD (MOS = −2.0) and triplet state ($\mathbf{3^T}$, MOS = −1.8) structures (**Figure S20**). This suggests the ONO ligand in $\mathbf{3^S}$ is $[^{bq}(ONO)]^{-}$, due to the contracted C2–O bonds and elongated C1–C2 and C2–C3 bonds compared to **3** and $\mathbf{3^T}$. The bond lengths determined experimentally by SC-XRD are best reproduced in the triplet structure.

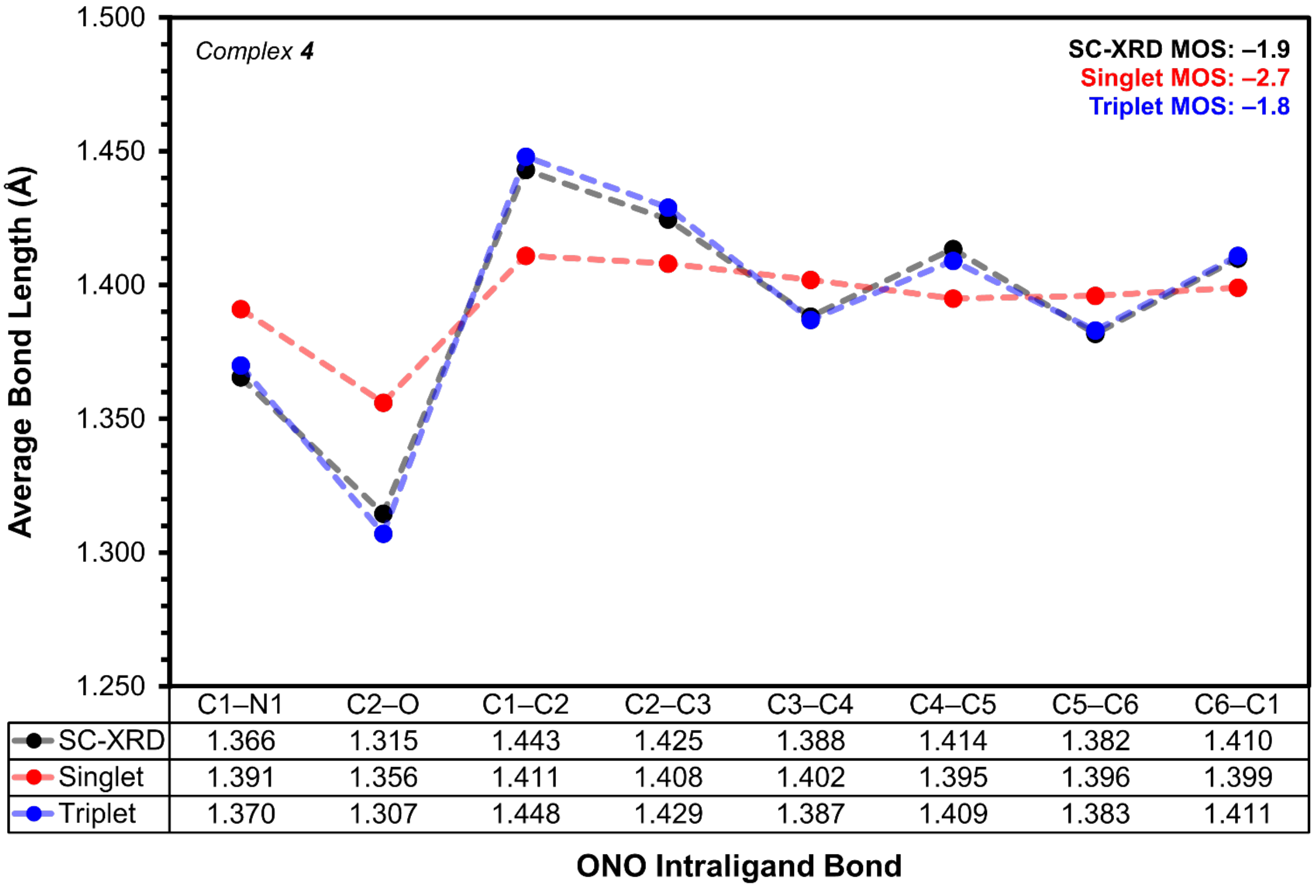

	C1–N1	C2–O	C1–C2	C2–C3	C3–C4	C4–C5	C5–C6	C6–C1
SC-XRD	1.366	1.315	1.443	1.425	1.388	1.414	1.382	1.410
Singlet	1.391	1.356	1.411	1.408	1.402	1.395	1.396	1.399
Triplet	1.370	1.307	1.448	1.429	1.387	1.409	1.383	1.411

ONO Intraligand Bond

Figure S21. Comparison of the average ONO intraligand bond lengths of the DFT-optimized structures of complex **4** in the open-shell singlet (S = 0, in red) and triplet (S = 1, in blue) spin states with the structure obtained experimentally by SC-XRD (in black). Average bond lengths (in angstroms) were derived from the chemically equivalent bonds in the ONO ligand.

The MOS value of the ONO ligand in the singlet structure of **4** (**4**$^{\mathbf{S}}$, MOS = −2.7) is significantly smaller than those of the SC-XRD (MOS = −1.9) and triplet state (**4**$^{\mathbf{T}}$, MOS = −1.8) structures (**Figure S21**). This suggests the ONO ligand in **4**$^{\mathbf{S}}$ is $[^{cat}(ONO)]^{3-}$, which is attributed to the elongated C1–N and C2–O bonds and considerable decrease in C–C bond length alternation compared to **4** and **4**$^{\mathbf{T}}$. The bond lengths determined experimentally by SC-XRD are best reproduced in the triplet structure.

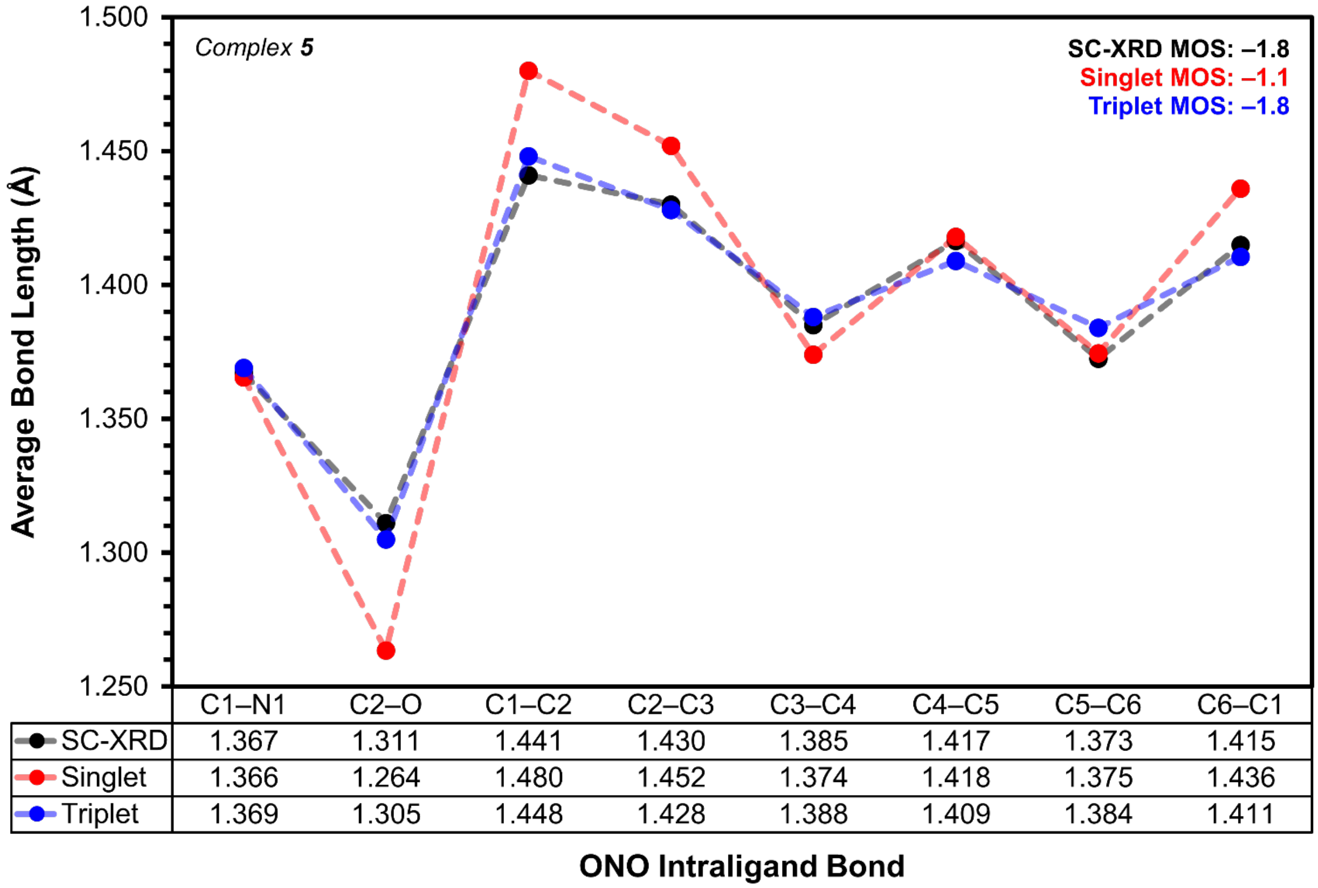

	C1–N1	C2–O	C1–C2	C2–C3	C3–C4	C4–C5	C5–C6	C6–C1
SC-XRD	1.367	1.311	1.441	1.430	1.385	1.417	1.373	1.415
Singlet	1.366	1.264	1.480	1.452	1.374	1.418	1.375	1.436
Triplet	1.369	1.305	1.448	1.428	1.388	1.409	1.384	1.411

ONO Intraligand Bond

Figure S22. Comparison of the average ONO intraligand bond lengths of the DFT-optimized structures of complex **5** in the open-shell singlet (S = 0, in red) and triplet (S = 1, in blue) spin states with the structure obtained experimentally by SC-XRD (in black). Average bond lengths (in angstroms) were derived from the chemically equivalent bonds in the ONO ligand.

The MOS value of the ONO ligand in the singlet structure of **5** (**5**^{S}, MOS = −1.1) is significantly greater than those of the SC-XRD (MOS = −1.8) and triplet state (**5**^{T}, MOS = −1.8) structures (**Figure S22**). This suggests the ONO ligand in **5**^{S} is [^{bq}(ONO)]$^{-}$, which is attributed to the contracted C1–N and C2–O bonds and elongated C1–C2 and C2–C3 bonds compared to **5** and **5**^{T}. The bond lengths determined experimentally by SC-XRD are best reproduced in the triplet structure.

6.3 Optimized Cartesian Coordinates

Table S21. Optimized Cartesian coordinates for **1-cat^D^** (*S* = ½, doublet state).

Cu	-0.042729	-1.607852	-0.060567	H	-3.503005	4.296240	2.152874
C	2.307465	-0.238031	0.003762	C	-4.027365	4.546493	-1.266546
C	3.681291	0.101968	0.008941	H	-4.950160	3.959938	-1.324731
C	4.028313	1.460093	-0.014913	H	-3.440859	4.338704	-2.169340
H	5.080396	1.724090	0.006340	H	-4.301477	5.609413	-1.273758
C	3.086728	2.491067	-0.056917	C	-1.982753	5.105282	0.021572
C	1.732196	2.144343	-0.082618	H	-2.294688	6.155916	0.035415
H	0.995584	2.931020	-0.132619	H	-1.349600	4.960682	-0.861514
C	1.310518	0.803176	-0.067657	H	-1.368760	4.930040	0.912665
C	-1.212169	0.896512	-0.058381	C	-4.776041	-0.649688	-0.035828
C	-1.533954	2.265317	-0.033760	C	-4.664504	-1.538613	-1.286798
H	-0.741183	2.997129	-0.039835	H	-5.460409	-2.294736	-1.291830
C	-2.859722	2.710021	-0.016588	H	-3.697997	-2.047322	-1.315001
C	-3.874823	1.749871	-0.023749	H	-4.767276	-0.932237	-2.195032
H	-4.905305	2.089745	-0.022600	C	-4.717890	-1.527601	1.226141
C	-3.628106	0.369544	-0.026217	H	-5.538254	-2.257290	1.220796
C	-2.282806	-0.070134	-0.007662	H	-4.821479	-0.908969	2.126147
C	4.751722	-0.996833	0.064415	H	-3.769002	-2.066386	1.279255
C	6.176479	-0.435568	0.044029	C	-6.155403	0.014436	-0.067064
H	6.378132	0.206476	0.909253	H	-6.290290	0.640324	-0.956863
H	6.376709	0.142478	-0.865588	H	-6.332430	0.635790	0.818563
H	6.892805	-1.265059	0.074264	H	-6.930052	-0.761124	-0.087007
C	4.609114	-1.926640	-1.153069	C	-0.246856	-3.453853	-2.779143
H	5.356028	-2.730123	-1.110836	H	-1.196990	-3.977429	-2.923818
H	4.768011	-1.365305	-2.081855	H	0.340662	-3.576289	-3.694914
H	3.613263	-2.374851	-1.184224	H	-0.461336	-2.385380	-2.653406
C	4.598284	-1.812189	1.360273	C	0.547938	-3.997373	-1.609399
H	4.743301	-1.166512	2.235085	H	0.703033	-5.080768	-1.732951
H	5.350398	-2.611063	1.401039	H	1.539462	-3.532638	-1.595127
H	3.604488	-2.262059	1.422476	C	0.466969	-4.012185	2.179123
C	3.554264	3.950973	-0.071549	H	0.316844	-2.938966	2.346967
C	2.389993	4.943252	-0.137180	H	1.267391	-4.347402	2.847081
H	1.788864	4.807436	-1.043754	H	-0.447290	-4.539927	2.468557
H	2.778223	5.968157	-0.147965	C	0.876366	-4.284687	0.747299
H	1.724439	4.851015	0.728812	H	1.849733	-3.823060	0.560169
C	4.354653	4.246537	1.207360	H	0.990933	-5.367712	0.586027
H	3.731933	4.088963	2.095958	C	-1.621038	-5.736165	-0.277307
H	4.705741	5.286547	1.215452	H	-1.312237	-6.112785	-1.258344
H	5.230070	3.594476	1.292687	H	-2.686970	-5.961016	-0.161837
C	4.451677	4.196345	-1.295017	H	-1.082965	-6.302993	0.489757
H	5.330037	3.542786	-1.287703	C	-1.426548	-4.235098	-0.140780
H	4.804754	5.235442	-1.316042	H	-2.033054	-3.701868	-0.875054
H	3.899316	4.004141	-2.222394	H	-1.798894	-3.889691	0.826819
C	-3.218074	4.200938	-0.005876	N	0.028224	0.302765	-0.143505
C	-4.062824	4.522460	1.237646	N	-0.040680	-3.720885	-0.274666
H	-4.988068	3.937452	1.258461	O	1.891779	-1.493036	0.076459
H	-4.335558	5.585483	1.257404	O	-1.961817	-1.352868	0.065360

Table S22. Optimized Cartesian coordinates for **1-cat**^{Q} (S = 3/2, quadruplet).

Cu	-0.116036	1.622660	0.014380	H	-3.483053	-3.977909	-2.646979
C	2.297642	0.350908	-0.036523	C	-3.672056	-4.906863	0.689846
C	3.676604	0.057814	-0.066783	H	-4.603489	-4.390578	0.943248
C	4.053708	-1.307146	0.035936	H	-3.007723	-4.846440	1.560098
H	5.108826	-1.553735	0.013469	H	-3.908299	-5.964016	0.512114
C	3.130037	-2.356958	0.269144	C	-1.742151	-5.106630	-0.853616
C	1.779754	-2.067226	0.297872	H	-2.016360	-6.152630	-1.032754
H	1.049964	-2.835172	0.514682	H	-1.030186	-5.086164	-0.020216
C	1.324936	-0.728741	0.034063	H	-1.227896	-4.739196	-1.749213
C	-1.161294	-0.927433	-0.013346	C	-4.799796	0.405393	0.375777
C	-1.404537	-2.311563	-0.319040	C	-4.645553	1.164209	1.706621
H	-0.568969	-2.944539	-0.585191	H	-5.492862	1.844811	1.864852
C	-2.690454	-2.812854	-0.273402	H	-3.722111	1.749180	1.717132
C	-3.761833	-1.932005	0.022674	H	-4.618229	0.457542	2.545218
H	-4.763303	-2.344531	0.059342	C	-4.901111	1.417870	-0.780025
C	-3.604335	-0.527965	0.163548	H	-5.756656	2.091266	-0.629648
C	-2.290807	-0.017822	0.111987	H	-5.045939	0.893543	-1.732409
C	4.710827	1.176475	-0.221963	H	-3.992000	2.019447	-0.850312
C	6.145546	0.642570	-0.262916	C	-6.128396	-0.353783	0.433950
H	6.302404	-0.043167	-1.103645	H	-6.148653	-1.079943	1.254921
H	6.412368	0.116607	0.661077	H	-6.334570	-0.889052	-0.500145
H	6.843129	1.480113	-0.382026	H	-6.947570	0.356565	0.597651
C	4.622179	2.156747	0.962326	C	-0.585673	3.678453	2.520150
H	5.360641	2.963412	0.853174	H	-1.551690	4.191868	2.552930
H	4.828442	1.633882	1.903995	H	-0.063216	3.895725	3.457194
H	3.626122	2.601333	1.025863	H	-0.777614	2.599320	2.480596
C	4.467703	1.938673	-1.537650	C	0.277821	4.135917	1.362000
H	4.582458	1.263000	-2.394036	H	0.397575	5.229510	1.391423
H	5.192471	2.755984	-1.651814	H	1.280462	3.704615	1.457705
H	3.460019	2.360778	-1.565422	C	0.477871	3.779223	-2.408074
C	3.661127	-3.777223	0.489204	H	0.439437	2.684560	-2.466379
C	2.543993	-4.792161	0.741624	H	1.296813	4.116245	-3.052118
H	1.968956	-4.546421	1.641958	H	-0.452644	4.183183	-2.818862
H	2.975295	-5.789742	0.883589	C	0.748755	4.239152	-0.991639
H	1.846797	-4.847188	-0.102621	H	1.733737	3.879865	-0.683257
C	4.434667	-4.230495	-0.759785	H	0.772637	5.337735	-0.945310
H	3.776638	-4.237004	-1.636909	C	-1.868487	5.655667	-0.245011
H	4.830989	-5.244813	-0.621518	H	-1.639345	6.121406	0.719278
H	5.276346	-3.565455	-0.978247	H	-2.933684	5.818889	-0.441226
C	4.602655	-3.798237	1.703904	H	-1.308315	6.185487	-1.022173
H	5.453867	-3.123806	1.566885	C	-1.605262	4.159470	-0.246193
H	4.994979	-4.809302	1.872116	H	-2.238847	3.658771	0.487795
H	4.068231	-3.483649	2.608335	H	-1.892005	3.726914	-1.208622
C	-2.998374	-4.288638	-0.546246	N	0.038896	-0.293749	0.011461
C	-3.946784	-4.406045	-1.750151	N	-0.209532	3.721718	0.019640
H	-4.887748	-3.875241	-1.574496	O	1.836183	1.584761	-0.074950
H	-4.183737	-5.457741	-1.955800	O	-2.029702	1.271936	0.180590

Table S23. Optimized Cartesian coordinates for $\mathbf{1}^{\mathrm{S}}$ (S = 0, open-shell singlet state).

Cu	0.110178	1.615373	0.000000	H	3.304386	-4.423727	2.163537
C	-2.286058	0.340034	0.000000	C	3.871272	-4.656027	-1.254621
C	-3.683349	0.053019	0.000000	H	4.813104	-4.099105	-1.293721
C	-4.061278	-1.281152	0.000000	H	3.304386	-4.423727	-2.163537
H	-5.121034	-1.511971	0.000000	H	4.114796	-5.725232	-1.266737
C	-3.154812	-2.363947	0.000000	C	1.792400	-5.178556	0.000000
C	-1.802328	-2.081210	0.000000	H	2.076467	-6.236407	0.000000
H	-1.098562	-2.896944	0.000000	H	1.175114	-5.003479	-0.888806
C	-1.331143	-0.751900	0.000000	H	1.175114	-5.003479	0.888806
C	1.168481	-0.948286	0.000000	C	4.802242	0.465843	0.000000
C	1.436120	-2.333975	0.000000	C	4.754781	1.348000	-1.259951
H	0.620194	-3.037892	0.000000	H	5.601651	2.045154	-1.261677
C	2.732001	-2.814662	0.000000	H	3.830079	1.927026	-1.305460
C	3.790949	-1.879448	0.000000	H	4.823067	0.729928	-2.163026
H	4.804281	-2.266398	0.000000	C	4.754781	1.348000	1.259951
C	3.616205	-0.504362	0.000000	H	5.601651	2.045154	1.261677
C	2.276923	-0.013177	0.000000	H	4.823067	0.729928	2.163026
C	-4.715331	1.185867	0.000000	H	3.830079	1.927026	1.305460
C	-6.153864	0.661523	0.000000	C	6.148216	-0.263483	0.000000
H	-6.372908	0.058973	0.888943	H	6.276693	-0.891896	-0.888842
H	-6.372908	0.058973	-0.888943	H	6.276693	-0.891896	0.888842
H	-6.845416	1.511326	0.000000	H	6.956791	0.475752	0.000000
C	-4.540785	2.050626	-1.260697	C	0.032813	3.687591	-2.520926
H	-5.273101	2.867181	-1.260779	H	1.006649	4.135681	-2.739752
H	-4.703462	1.449590	-2.163147	H	-0.649753	3.971455	-3.328221
H	-3.539674	2.483356	-1.311307	H	0.142840	2.596119	-2.548876
C	-4.540785	2.050626	1.260697	C	-0.547759	4.156615	-1.203823
H	-4.703462	1.449590	2.163147	H	-0.620710	5.253412	-1.189502
H	-5.273101	2.867181	1.260779	H	-1.563698	3.767101	-1.093328
H	-3.539674	2.483356	1.311307	C	0.032813	3.687591	2.520926
C	-3.692448	-3.795606	0.000000	H	0.142840	2.596119	2.548876
C	-2.573904	-4.839984	0.000000	H	-0.649753	3.971455	3.328221
H	-1.937459	-4.757427	-0.888609	H	1.006649	4.135681	2.739752
H	-3.010133	-5.844666	0.000000	C	-0.547759	4.156615	1.203823
H	-1.937459	-4.757427	0.888609	H	-1.563698	3.767101	1.093328
C	-4.552831	-4.015870	1.254903	H	-0.620710	5.253412	1.189502
H	-3.958401	-3.864389	2.163321	C	1.854089	5.644578	0.000000
H	-4.948036	-5.038669	1.270652	H	1.443943	6.137561	-0.887129
H	-5.404313	-3.328561	1.291389	H	2.935165	5.819019	0.000000
C	-4.552831	-4.015870	-1.254903	H	1.443943	6.137561	0.887129
H	-5.404313	-3.328561	-1.291389	C	1.614105	4.145332	0.000000
H	-4.948036	-5.038669	-1.270652	H	2.092007	3.683404	-0.866448
H	-3.958401	-3.864389	-2.163321	H	2.092007	3.683404	0.866448
C	3.052318	-4.310111	0.000000	N	-0.039492	-0.316142	0.000000
C	3.871272	-4.656027	1.254621	N	0.195410	3.695907	0.000000
H	4.813104	-4.099105	1.293721	O	-1.837115	1.566635	0.000000
H	4.114796	-5.725232	1.266737	O	2.020201	1.265935	0.000000

Table S24. Optimized Cartesian coordinates for **1**^{T} (S = 1, triplet state).

Cu	0.110168	1.615341	0.000000	H	3.303469	-4.424113	2.163519
C	-2.285992	0.339428	0.000000	C	3.870257	-4.656651	-1.254598
C	-3.683322	0.052629	0.000000	H	4.812286	-4.100050	-1.293688
C	-4.061022	-1.281575	0.000000	H	3.303469	-4.424113	-2.163519
H	-5.120771	-1.512485	0.000000	H	4.113407	-5.725941	-1.266759
C	-3.154521	-2.364027	0.000000	C	1.791170	-5.178244	0.000000
C	-1.802022	-2.080815	0.000000	H	2.074747	-6.236228	0.000000
H	-1.098014	-2.896285	0.000000	H	1.173958	-5.002912	-0.888813
C	-1.332033	-0.751401	0.000000	H	1.173958	-5.002912	0.888813
C	1.169489	-0.947933	0.000000	C	4.802140	0.465443	0.000000
C	1.435923	-2.333542	0.000000	C	4.754589	1.347628	-1.259927
H	0.619791	-3.037153	0.000000	H	5.601527	2.044699	-1.261741
C	2.731737	-2.814713	0.000000	H	3.829936	1.926748	-1.305283
C	3.790643	-1.879843	0.000000	H	4.822689	0.729586	-2.163036
H	4.803961	-2.266867	0.000000	C	4.754589	1.347628	1.259927
C	3.616121	-0.504741	0.000000	H	5.601527	2.044699	1.261741
C	2.276779	-0.013773	0.000000	H	4.822689	0.729586	2.163036
C	-4.715230	1.185494	0.000000	H	3.829936	1.926748	1.305283
C	-6.153773	0.661204	0.000000	C	6.148125	-0.263838	0.000000
H	-6.372822	0.058655	0.888942	H	6.276610	-0.892249	-0.888842
H	-6.372822	0.058655	-0.888942	H	6.276610	-0.892249	0.888842
H	-6.845300	1.511028	0.000000	H	6.956680	0.475419	0.000000
C	-4.540544	2.050254	-1.260681	C	0.032462	3.687499	-2.520640
H	-5.272921	2.866755	-1.260877	H	1.006640	4.134639	-2.739907
H	-4.703034	1.449207	-2.163154	H	-0.649929	3.971199	-3.328144
H	-3.539449	2.483057	-1.311136	H	0.141709	2.595902	-2.547940
C	-4.540544	2.050254	1.260681	C	-0.547567	4.157911	-1.203749
H	-4.703034	1.449207	2.163154	H	-0.619987	5.254767	-1.190319
H	-5.272921	2.866755	1.260877	H	-1.563753	3.769048	-1.092962
H	-3.539449	2.483057	1.311136	C	0.032462	3.687499	2.520640
C	-3.691629	-3.795955	0.000000	H	0.141709	2.595902	2.547940
C	-2.572620	-4.839843	0.000000	H	-0.649929	3.971199	3.328144
H	-1.936208	-4.757026	-0.888616	H	1.006640	4.134639	2.739907
H	-3.008391	-5.844724	0.000000	C	-0.547567	4.157911	1.203749
H	-1.936208	-4.757026	0.888616	H	-1.563753	3.769048	1.092962
C	-4.551910	-4.016608	1.254879	H	-0.619987	5.254767	1.190319
H	-3.957529	-3.864944	2.163303	C	1.854324	5.646218	0.000000
H	-4.946824	-5.039523	1.270632	H	1.444204	6.139268	-0.887109
H	-5.403596	-3.329543	1.291389	H	2.935404	5.820682	0.000000
C	-4.551910	-4.016608	-1.254879	H	1.444204	6.139268	0.887109
H	-5.403596	-3.329543	-1.291389	C	1.614150	4.146994	0.000000
H	-4.946824	-5.039523	-1.270632	H	2.092197	3.685098	-0.866413
H	-3.957529	-3.864944	-2.163303	H	2.092197	3.685098	0.866413
C	3.051476	-4.310358	0.000000	N	-0.039375	-0.314749	0.000000
C	3.870257	-4.656651	1.254598	N	0.195570	3.697186	0.000000
H	4.812286	-4.100050	1.293688	O	-1.836644	1.566277	0.000000
H	4.113407	-5.725941	1.266759	O	2.019605	1.265673	0.000000

Table S25. Optimized Cartesian coordinates for **1-bq^{D}** (S = ½, doublet state).

Cu	-0.120603	1.664822	-0.017593	H	-3.413704	-4.020516	-2.618271
C	2.272583	0.363456	-0.034762	C	-3.696581	-4.814400	0.755245
C	3.676258	0.057559	-0.067879	H	-4.631984	-4.285032	0.962732
C	4.014910	-1.266198	0.069137	H	-3.054560	-4.727957	1.638898
H	5.068992	-1.521854	0.056660	H	-3.941021	-5.872763	0.610001
C	3.090962	-2.341631	0.261055	C	-1.727688	-5.107548	-0.737756
C	1.751594	-2.058569	0.252123	H	-2.012294	-6.153450	-0.890664
H	1.031399	-2.839694	0.440805	H	-1.042396	-5.076513	0.117522
C	1.303276	-0.729580	0.060909	H	-1.184816	-4.780530	-1.631851
C	-1.146876	-0.924259	-0.088393	C	-4.808539	0.402450	0.317799
C	-1.382902	-2.305356	-0.290325	C	-4.644524	1.172001	1.640916
H	-0.552651	-2.958961	-0.509727	H	-5.511081	1.824712	1.796079
C	-2.660126	-2.796974	-0.271704	H	-3.745813	1.792499	1.643120
C	-3.737934	-1.884680	-0.036472	H	-4.587468	0.477771	2.486993
H	-4.736749	-2.306072	0.001811	C	-4.916484	1.389337	-0.858681
C	-3.611488	-0.525473	0.110079	H	-5.788566	2.037435	-0.714601
C	-2.275607	0.001208	0.036366	H	-5.047500	0.850666	-1.804064
C	4.714539	1.167445	-0.229561	H	-4.030587	2.021917	-0.941290
C	6.139412	0.610550	-0.264267	C	-6.122888	-0.377541	0.392687
H	6.291264	-0.079565	-1.102089	H	-6.130951	-1.092369	1.223490
H	6.403582	0.093818	0.665393	H	-6.334428	-0.919998	-0.535758
H	6.843590	1.439525	-0.389172	H	-6.946411	0.325331	0.555545
C	4.622887	2.142869	0.957692	C	-0.509964	3.545403	2.548214
H	5.380474	2.927455	0.848891	H	-1.509397	3.985296	2.610206
H	4.809833	1.619788	1.902281	H	0.012413	3.780391	3.480265
H	3.643005	2.620713	1.017031	H	-0.618575	2.454217	2.501482
C	4.473252	1.915398	-1.553171	C	0.300651	4.089193	1.390699
H	4.564638	1.231618	-2.404657	H	0.366853	5.183688	1.451523
H	5.223566	2.705595	-1.670734	H	1.326165	3.709589	1.445631
H	3.484019	2.376451	-1.588068	C	0.447996	3.782094	-2.396618
C	3.637771	-3.748639	0.468033	H	0.436897	2.687195	-2.473282
C	2.525957	-4.778643	0.671352	H	1.244891	4.145077	-3.053014
H	1.921358	-4.558177	1.558460	H	-0.499575	4.164684	-2.786208
H	2.968718	-5.769638	0.813436	C	0.735322	4.242287	-0.984608
H	1.860806	-4.839244	-0.198088	H	1.733057	3.911383	-0.686600
C	4.451953	-4.150863	-0.773970	H	0.734287	5.338312	-0.928623
H	3.821308	-4.146682	-1.669989	C	-1.859195	5.648908	-0.184241
H	4.855164	-5.161302	-0.642085	H	-1.601392	6.110208	0.774062
H	5.297517	-3.478440	-0.951105	H	-2.928508	5.815108	-0.349785
C	4.546592	-3.761204	1.708601	H	-1.320162	6.173603	-0.978492
H	5.393212	-3.074522	1.608263	C	-1.611022	4.151956	-0.195353
H	4.952927	-4.767323	1.861710	H	-2.229306	3.662491	0.558534
H	3.984518	-3.477441	2.605384	H	-1.919562	3.725091	-1.153412
C	-2.982896	-4.269089	-0.493457	N	0.035211	-0.293139	-0.015797
C	-3.904991	-4.404181	-1.717169	N	-0.205794	3.707532	0.040738
H	-4.846638	-3.861209	-1.587158	O	1.847388	1.570563	-0.099093
H	-4.151174	-5.459110	-1.882709	O	-2.047970	1.259872	0.089190

Table S26. Optimized Cartesian coordinates for **1-bq^{Q}** (S = 3/2, quadruplet state).

Cu	-0.117511	1.640703	-0.005349
C	2.285920	0.339029	-0.058930
C	3.690738	0.052919	-0.072914
C	4.048757	-1.271244	0.075379
H	5.103124	-1.521517	0.071044
C	3.125293	-2.331374	0.260683
C	1.763050	-2.049307	0.248281
H	1.052349	-2.841147	0.428779
C	1.314433	-0.744121	0.044455
C	-1.155766	-0.939118	-0.072341
C	-1.397049	-2.294914	-0.291635
H	-0.574754	-2.960848	-0.504243
C	-2.697963	-2.789080	-0.277273
C	-3.772284	-1.890902	-0.054413
H	-4.773544	-2.304864	-0.032924
C	-3.624609	-0.528952	0.111196
C	-2.282489	-0.024463	0.074296
C	4.716092	1.172675	-0.230415
C	6.148561	0.634700	-0.254280
H	6.314706	-0.055525	-1.089080
H	6.413190	0.124176	0.678454
H	6.842206	1.472795	-0.376996
C	4.601911	2.148758	0.955368
H	5.352932	2.940379	0.852678
H	4.784218	1.628755	1.902476
H	3.616355	2.616071	1.003163
C	4.473835	1.916670	-1.556771
H	4.573469	1.232406	-2.406877
H	5.219035	2.711901	-1.672115
H	3.481123	2.369916	-1.595582
C	3.653303	-3.745131	0.460815
C	2.535083	-4.771536	0.651387
H	1.927324	-4.556371	1.537449
H	2.974371	-5.764656	0.788605
H	1.874387	-4.825056	-0.221770
C	4.473941	-4.145877	-0.778742
H	3.851445	-4.126263	-1.680112
H	4.860590	-5.163125	-0.650169
H	5.328968	-3.482498	-0.940869
C	4.555665	-3.770922	1.707411
H	5.410172	-3.094044	1.612672
H	4.946838	-4.783498	1.857356
H	3.991784	-3.484293	2.601984
C	-3.000065	-4.266267	-0.485824
C	-3.912900	-4.422093	-1.715485
H	-4.861433	-3.889866	-1.595437
H	-4.142171	-5.482285	-1.871154

H	-3.419920	-4.041157	-2.616657
C	-3.720479	-4.804052	0.763902
H	-4.664879	-4.284644	0.952542
H	-3.089360	-4.696811	1.653018
H	-3.945882	-5.867854	0.628498
C	-1.738199	-5.100938	-0.711562
H	-2.018263	-6.149481	-0.853734
H	-1.059026	-5.058239	0.147927
H	-1.190863	-4.783662	-1.606279
C	-4.810870	0.411162	0.310656
C	-4.661058	1.159968	1.648050
H	-5.524630	1.817755	1.798022
H	-3.756598	1.771577	1.673102
H	-4.624050	0.453081	2.484655
C	-4.877828	1.417367	-0.853496
H	-5.744478	2.074625	-0.719024
H	-4.993615	0.895101	-1.809930
H	-3.981479	2.038362	-0.904813
C	-6.138971	-0.348198	0.348232
H	-6.175760	-1.072663	1.169613
H	-6.339608	-0.875684	-0.590998
H	-6.954038	0.366270	0.502905
C	-0.476012	3.561146	2.558017
H	-1.473030	4.005759	2.626212
H	0.058819	3.808044	3.479951
H	-0.589138	2.470190	2.527285
C	0.321312	4.086248	1.382816
H	0.391647	5.181240	1.428821
H	1.346452	3.704597	1.430136
C	0.411157	3.768496	-2.406048
H	0.386309	2.674335	-2.486385
H	1.202427	4.125729	-3.072422
H	-0.537460	4.163466	-2.780591
C	0.723719	4.220951	-0.996856
H	1.724471	3.883664	-0.716231
H	0.730800	5.316911	-0.937814
C	-1.860267	5.629568	-0.166314
H	-1.595942	6.092942	0.789208
H	-2.930849	5.794976	-0.324494
H	-1.327266	6.153544	-0.965191
C	-1.610733	4.132882	-0.176832
H	-2.220251	3.645005	0.585348
H	-1.929957	3.704431	-1.130635
N	0.035939	-0.287188	-0.018972
N	-0.202634	3.688459	0.043439
O	1.833812	1.545786	-0.125567
O	-2.024473	1.236443	0.160943

Table S27. Optimized Cartesian coordinates for $\mathbf{2^S}$ (S = 0, open-shell singlet state).

Cu	-0.440371	-1.570399	0.000000	H	4.752135	2.854790	-2.163248
C	2.183142	-0.873362	0.000000	C	-1.952225	4.862174	0.000000
C	3.609802	-0.905008	0.000000	C	-2.668405	5.387454	1.254888
C	4.275413	0.311328	0.000000	H	-3.713211	5.062584	1.294664
H	5.359976	0.301538	0.000000	H	-2.658908	6.484023	1.267465
C	3.632467	1.568738	0.000000	H	-2.169379	5.030384	2.163195
C	2.250851	1.594744	0.000000	C	-2.668405	5.387454	-1.254888
H	1.748882	2.547661	0.000000	H	-3.713211	5.062584	-1.294664
C	1.494516	0.403722	0.000000	H	-2.169379	5.030384	-2.163195
C	-0.899100	1.155204	0.000000	H	-2.658908	6.484023	-1.267465
C	-0.839394	2.564730	0.000000	C	-0.525119	5.414573	0.000000
H	0.117137	3.059785	0.000000	H	-0.556012	6.509501	0.000000
C	-1.988009	3.333249	0.000000	H	0.034779	5.101016	-0.888780
C	-3.234293	2.669232	0.000000	H	0.034779	5.101016	0.888780
H	-4.130383	3.280452	0.000000	C	-4.761918	0.623767	0.000000
C	-3.382489	1.290818	0.000000	C	-4.921692	-0.245223	-1.259539
C	-2.194016	0.502110	0.000000	H	-5.908550	-0.724105	-1.262663
C	4.362482	-2.239754	0.000000	H	-4.158078	-1.024776	-1.305358
C	5.881388	-2.047111	0.000000	H	-4.841435	0.370625	-2.163153
H	6.228041	-1.507292	0.888784	C	-4.921692	-0.245223	1.259539
H	6.228041	-1.507292	-0.888784	H	-5.908550	-0.724105	1.262663
H	6.368069	-3.028693	0.000000	H	-4.841435	0.370625	2.163153
C	3.998012	-3.045974	-1.258789	H	-4.158078	-1.024776	1.305358
H	4.541177	-3.998971	-1.263697	C	-5.900348	1.647577	0.000000
H	4.277188	-2.492945	-2.163521	H	-5.877899	2.288756	-0.888652
H	2.927211	-3.257460	-1.298996	H	-5.877899	2.288756	0.888652
C	3.998012	-3.045974	1.258789	H	-6.859482	1.118060	0.000000
H	4.277188	-2.492945	2.163521	O	-2.239401	-0.803044	0.000000
H	4.541177	-3.998971	1.263697	N	0.136582	0.265365	0.000000
H	2.927211	-3.257460	1.298996	O	1.473757	-1.969401	0.000000
C	4.476894	2.843872	0.000000	N	-1.045284	-3.434594	0.000000
C	3.621468	4.112722	0.000000	C	-0.267496	-4.565890	0.000000
H	2.982766	4.175747	-0.888661	C	-2.310187	-3.841605	0.000000
H	4.273103	4.993207	0.000000	H	0.812288	-4.506074	0.000000
H	2.982766	4.175747	0.888661	C	-1.084976	-5.661913	0.000000
C	5.365240	2.865716	1.254622	N	-2.371212	-5.183645	0.000000
H	4.752135	2.854790	2.163248	H	-3.173732	-3.190790	0.000000
H	5.982223	3.772300	1.268426	H	-0.870323	-6.721552	0.000000
H	6.039102	2.003594	1.292478	C	-3.577202	-5.988968	0.000000
C	5.365240	2.865716	-1.254622	H	-4.445630	-5.328780	0.000000
H	6.039102	2.003594	-1.292478	H	-3.603013	-6.619756	-0.892442
H	5.982223	3.772300	-1.268426	H	-3.603013	-6.619756	0.892442

Table S28. Optimized Cartesian coordinates for $\mathbf{2^T}$ (S = 1, triplet state).

Cu	-0.060828	1.633463	0.000000	H	4.014446	-3.834332	2.163425
C	2.342616	0.369868	0.000000	C	-2.968317	-4.302127	0.000000
C	3.740697	0.082647	0.000000	C	-3.783832	-4.655763	-1.254635
C	4.118270	-1.251260	0.000000	H	-4.731039	-4.107983	-1.293568
H	5.177919	-1.482747	0.000000	H	-4.016962	-5.727267	-1.266973
C	3.211055	-2.333349	0.000000	H	-3.219224	-4.417846	-2.163558
C	1.858420	-2.050122	0.000000	C	-3.783832	-4.655763	1.254635
H	1.156042	-2.866554	0.000000	H	-4.731039	-4.107983	1.293568
C	1.387527	-0.720832	0.000000	H	-3.219224	-4.417846	2.163558
C	-1.115302	-0.923157	0.000000	H	-4.016962	-5.727267	1.266973
C	-1.371116	-2.310517	0.000000	C	-1.699970	-5.158222	0.000000
H	-0.549959	-3.007620	0.000000	H	-1.973778	-6.218789	0.000000
C	-2.662627	-2.803547	0.000000	H	-1.084378	-4.977260	0.888818
C	-3.729219	-1.878433	0.000000	H	-1.084378	-4.977260	-0.888818
H	-4.739250	-2.273878	0.000000	C	-4.761040	0.457584	0.000000
C	-3.565937	-0.501685	0.000000	C	-4.722486	1.339496	1.260199
C	-2.231033	0.001180	0.000000	H	-5.573025	2.032160	1.259812
C	4.772098	1.216043	0.000000	H	-3.800694	1.923097	1.311415
C	6.210506	0.691504	0.000000	H	-4.790431	0.720884	2.162951
H	6.429445	0.088744	-0.888894	C	-4.722486	1.339496	-1.260199
H	6.429445	0.088744	0.888894	H	-5.573025	2.032160	-1.259812
H	6.902307	1.541115	0.000000	H	-4.790431	0.720884	-2.162951
C	4.596273	2.082062	1.259521	H	-3.800694	1.923097	-1.311415
H	5.333206	2.894538	1.261579	C	-6.100503	-0.283654	0.000000
H	4.753200	1.481162	2.163199	H	-6.223348	-0.913121	0.888844
H	3.596840	2.520458	1.304592	H	-6.223348	-0.913121	-0.888844
C	4.596273	2.082062	-1.259521	H	-6.915493	0.448522	0.000000
H	4.753200	1.481162	-2.163199	O	-1.983270	1.283645	0.000000
H	5.333206	2.894538	-1.261579	N	0.093211	-0.283994	0.000000
H	3.596840	2.520458	-1.304592	O	1.894760	1.596657	0.000000
C	3.748066	-3.765379	0.000000	N	-0.243468	3.586114	0.000000
C	2.629131	-4.809364	0.000000	C	0.761493	4.521343	0.000000
H	1.992672	-4.726640	0.888614	C	-1.389717	4.258657	0.000000
H	3.065103	-5.814173	0.000000	H	1.802671	4.228380	0.000000
H	1.992672	-4.726640	-0.888614	C	0.201772	5.769104	0.000000
C	4.608553	-3.986047	-1.254807	N	-1.157756	5.581598	0.000000
H	4.014446	-3.834332	-2.163425	H	-2.374295	3.811354	0.000000
H	5.003229	-5.009086	-1.270436	H	0.641107	6.756994	0.000000
H	5.460541	-3.299300	-1.291091	C	-2.159887	6.629779	0.000000
C	4.608553	-3.986047	1.254807	H	-3.151218	6.174548	0.000000
H	5.460541	-3.299300	1.291091	H	-2.047739	7.250815	0.892525
H	5.003229	-5.009086	1.270436	H	-2.047739	7.250815	-0.892525

Table S29. Optimized Cartesian coordinates for **3**^{S} (S = 0, open-shell singlet state).

Cu	0.000000	0.000000	-1.892741
C	-2.367759	0.000000	-0.238351
C	-3.733995	0.000000	0.269053
C	-3.920325	0.000000	1.623584
H	-4.934556	0.000000	2.009044
C	-2.858192	0.000000	2.573354
C	-1.566343	0.000000	2.114076
H	-0.788669	0.000000	2.853769
C	-1.233596	0.000000	0.730089
C	1.233596	-0.000000	0.730089
C	1.566343	-0.000000	2.114076
H	0.788669	-0.000000	2.853769
C	2.858192	-0.000000	2.573354
C	3.920325	-0.000000	1.623584
H	4.934556	-0.000000	2.009044
C	3.733995	-0.000000	0.269053
C	2.367759	-0.000000	-0.238351
C	-4.910892	0.000000	-0.711904
C	-6.260344	0.000000	0.011750
H	-6.392795	0.889181	0.638989
H	-6.392795	-0.889181	0.638989
H	-7.064677	0.000000	-0.731776
C	-4.863962	-1.260651	-1.594084
H	-5.718398	-1.264176	-2.281751
H	-4.924799	-2.164435	-0.976233
H	-3.945472	-1.302609	-2.182474
C	-4.863962	1.260651	-1.594084
H	-4.924799	2.164435	-0.976233
H	-5.718398	1.264176	-2.281751
H	-3.945472	1.302609	-2.182474
C	-3.208522	0.000000	4.059518
C	-1.970184	0.000000	4.956461
H	-1.349372	-0.888954	4.796574
H	-2.278988	0.000000	6.007122
H	-1.349372	0.888954	4.796574
C	-4.035838	1.255877	4.382995
H	-3.462341	2.164273	4.165864
H	-4.305161	1.266128	5.445669
H	-4.964723	1.297186	3.804921
C	-4.035838	-1.255877	4.382995
H	-4.964723	-1.297186	3.804921
H	-4.305161	-1.266128	5.445669
H	-3.462341	-2.164273	4.165864
C	3.208522	-0.000000	4.059518
C	4.035838	1.255877	4.382995
H	4.964723	1.297186	3.804921
H	4.305161	1.266128	5.445669
H	3.462341	2.164273	4.165864
C	4.035838	-1.255877	4.382995
H	4.964723	-1.297186	3.804921
H	3.462341	-2.164273	4.165864
H	4.305161	-1.266128	5.445669
C	1.970184	-0.000000	4.956461
H	2.278988	-0.000000	6.007122
H	1.349372	-0.888954	4.796574
H	1.349372	0.888954	4.796574
C	4.910892	-0.000000	-0.711904
C	4.863962	-1.260651	-1.594084
H	5.718398	-1.264176	-2.281751
H	3.945472	-1.302609	-2.182474
H	4.924799	-2.164435	-0.976233
C	4.863962	1.260651	-1.594084
H	5.718398	1.264176	-2.281751
H	4.924799	2.164435	-0.976233
H	3.945472	1.302609	-2.182474
C	6.260344	-0.000000	0.011750
H	6.392795	-0.889181	0.638989
H	6.392795	0.889181	0.638989
H	7.064677	-0.000000	-0.731776
O	2.155379	-0.000000	-1.477408
N	0.000000	0.000000	0.164150
H	0.000000	0.000000	-7.722019
O	-2.155379	0.000000	-1.477408
N	0.000000	0.000000	-3.845444
C	1.155167	-0.000000	-4.538539
C	-1.155167	0.000000	-4.538539
C	1.196466	-0.000000	-5.924583
C	-1.196466	0.000000	-5.924583
C	0.000000	0.000000	-6.634397
H	2.062927	-0.000000	-3.938876
H	-2.062927	0.000000	-3.938876
H	-2.156348	0.000000	-6.434529
H	2.156348	-0.000000	-6.434529

Table S30. Optimized Cartesian coordinates for **3**$^{\mathbf{T}}$ (S = 1, triplet state).

Cu	0.000000	0.000000	-1.678017
C	0.000000	2.292021	-0.219150
C	0.000000	3.663452	0.172529
C	0.000000	3.936314	1.531583
H	0.000000	4.974902	1.844479
C	0.000000	2.948187	2.540251
C	0.000000	1.621249	2.154396
H	0.000000	0.858237	2.914666
C	0.000000	1.255148	0.792037
C	-0.000000	-1.255148	0.792037
C	-0.000000	-1.621249	2.154396
H	-0.000000	-0.858237	2.914666
C	-0.000000	-2.948187	2.540251
C	-0.000000	-3.936314	1.531583
H	-0.000000	-4.974902	1.844479
C	-0.000000	-3.663452	0.172529
C	-0.000000	-2.292021	-0.219150
C	0.000000	4.779214	-0.878209
C	0.000000	6.173113	-0.244125
H	0.888963	6.344915	0.373585
H	-0.888963	6.344915	0.373585
H	0.000000	6.928103	-1.037958
C	-1.260400	4.672687	-1.754251
H	-1.259222	5.466672	-2.510893
H	-2.162957	4.789673	-1.142762
H	-1.313539	3.708815	-2.264936
C	1.260400	4.672687	-1.754251
H	2.162957	4.789673	-1.142762
H	1.259222	5.466672	-2.510893
H	1.313539	3.708815	-2.264936
C	0.000000	3.376772	4.008092
C	0.000000	2.183966	4.966390
H	-0.888803	1.555548	4.837070
H	0.000000	2.544777	6.000580
H	0.888803	1.555548	4.837070
C	1.254999	4.218603	4.291271
H	2.163545	3.635347	4.101909
H	1.267577	4.541726	5.339155
H	1.294409	5.115755	3.664936
C	-1.254999	4.218603	4.291271
H	-1.294409	5.115755	3.664936
H	-1.267577	4.541726	5.339155
H	-2.163545	3.635347	4.101909
C	-0.000000	-3.376772	4.008092
C	1.254999	-4.218603	4.291271
H	1.294409	-5.115755	3.664936
H	1.267577	-4.541726	5.339155
H	2.163545	-3.635347	4.101909
C	-1.254999	-4.218603	4.291271
H	-1.294409	-5.115755	3.664936
H	-2.163545	-3.635347	4.101909
H	-1.267577	-4.541726	5.339155
C	-0.000000	-2.183966	4.966390
H	-0.000000	-2.544777	6.000580
H	-0.888803	-1.555548	4.837070
H	0.888803	-1.555548	4.837070
C	-0.000000	-4.779214	-0.878209
C	-1.260400	-4.672687	-1.754251
H	-1.259222	-5.466672	-2.510893
H	-1.313539	-3.708815	-2.264936
H	-2.162957	-4.789673	-1.142762
C	1.260400	-4.672687	-1.754251
H	1.259222	-5.466672	-2.510893
H	2.162957	-4.789673	-1.142762
H	1.313539	-3.708815	-2.264936
C	-0.000000	-6.173113	-0.244125
H	-0.888963	-6.344915	0.373585
H	0.888963	-6.344915	0.373585
H	-0.000000	-6.928103	-1.037958
O	-0.000000	-1.939327	-1.477251
N	0.000000	0.000000	0.252678
H	0.000000	0.000000	-7.546503
O	0.000000	1.939327	-1.477251
N	0.000000	0.000000	-3.678559
C	-0.000000	-1.155684	-4.364550
C	0.000000	1.155684	-4.364550
C	-0.000000	-1.195985	-5.750113
C	0.000000	1.195985	-5.750113
C	0.000000	0.000000	-6.459026
H	-0.000000	-2.060960	-3.763888
H	0.000000	2.060960	-3.763888
H	0.000000	2.156085	-6.258849
H	-0.000000	-2.156085	-6.258849

Table S31. Optimized Cartesian coordinates for $\mathbf{4}^{\mathbf{S}}$ (S = 0, open-shell singlet state).

Cu	0.000000	0.000000	-1.287222	H	-3.731733	-2.162562	4.340818
C	2.267946	-0.000000	0.087414	C	-4.317591	1.254089	4.523373
C	3.636691	-0.000000	0.402287	H	-5.202477	1.290727	3.879777
C	3.963683	-0.000000	1.765992	H	-3.731733	2.162562	4.340818
H	5.014097	-0.000000	2.036069	H	-4.658526	1.270472	5.566015
C	3.013700	-0.000000	2.789818	C	-2.298045	0.000000	5.236295
C	1.658271	-0.000000	2.443325	H	-2.679727	0.000000	6.263220
H	0.907339	-0.000000	3.217796	H	-1.666659	0.888444	5.119357
C	1.280687	-0.000000	1.096179	H	-1.666659	-0.888444	5.119357
C	-1.280687	0.000000	1.096179	C	-4.697774	0.000000	-0.701047
C	-1.658271	0.000000	2.443325	C	-4.537936	1.259404	-1.570380
H	-0.907339	0.000000	3.217796	H	-5.282122	1.259735	-2.376605
C	-3.013700	0.000000	2.789818	H	-3.541202	1.311260	-2.016896
C	-3.963683	0.000000	1.765992	H	-4.690972	2.162251	-0.967249
H	-5.014097	0.000000	2.036069	C	-4.537936	-1.259404	-1.570380
C	-3.636691	0.000000	0.402287	H	-5.282122	-1.259735	-2.376605
C	-2.267946	0.000000	0.087414	H	-4.690972	-2.162251	-0.967249
C	4.697774	-0.000000	-0.701047	H	-3.541202	-1.311260	-2.016896
C	6.122088	-0.000000	-0.140670	C	-6.122088	0.000000	-0.140670
H	6.323086	-0.888521	0.468614	H	-6.323086	0.888521	0.468614
H	6.323086	0.888521	0.468614	H	-6.323086	-0.888521	0.468614
H	6.836800	-0.000000	-0.971406	H	-6.836800	0.000000	-0.971406
C	4.537936	1.259404	-1.570380	O	-1.824664	0.000000	-1.185981
H	5.282122	1.259735	-2.376605	N	0.000000	0.000000	0.557787
H	4.690972	2.162251	-0.967249	H	-0.000000	-5.264747	-4.250068
H	3.541202	1.311260	-2.016896	O	1.824664	-0.000000	-1.185981
C	4.537936	-1.259404	-1.570380	N	-0.000000	-1.765617	-2.593232
H	4.690972	-2.162251	-0.967249	C	-0.000000	-2.879726	-1.848273
H	5.282122	-1.259735	-2.376605	C	-0.000000	-1.894995	-3.923989
H	3.541202	-1.311260	-2.016896	C	-0.000000	-4.153154	-2.399389
C	3.471146	-0.000000	4.253059	C	-0.000000	-3.129701	-4.560680
C	2.298045	-0.000000	5.236295	C	-0.000000	-4.281970	-3.784050
H	1.666659	0.888444	5.119357	H	-0.000000	-2.737750	-0.767995
H	2.679727	-0.000000	6.263220	H	-0.000000	-0.978159	-4.505389
H	1.666659	-0.888444	5.119357	H	-0.000000	-3.176149	-5.646411
C	4.317591	-1.254089	4.523373	H	-0.000000	-5.022865	-1.747861
H	3.731733	-2.162562	4.340818	N	0.000000	1.765617	-2.593232
H	4.658526	-1.270472	5.566015	C	0.000000	1.894995	-3.923989
H	5.202477	-1.290727	3.879777	C	0.000000	2.879726	-1.848273
C	4.317591	1.254089	4.523373	C	0.000000	3.129701	-4.560680
H	5.202477	1.290727	3.879777	H	0.000000	0.978159	-4.505389
H	4.658526	1.270472	5.566015	C	0.000000	4.153154	-2.399389
H	3.731733	2.162562	4.340818	H	0.000000	2.737750	-0.767995
C	-3.471146	0.000000	4.253059	C	0.000000	4.281970	-3.784050
C	-4.317591	-1.254089	4.523373	H	0.000000	3.176149	-5.646411
H	-5.202477	-1.290727	3.879777	H	0.000000	5.022865	-1.747861
H	-4.658526	-1.270472	5.566015	H	0.000000	5.264747	-4.250068

Table S32. Optimized Cartesian coordinates for $\mathbf{4^T}$ (S = 1, triplet state).

Cu	0.000000	0.000000	-1.277119
C	-2.302202	0.000000	0.209135
C	-3.670364	0.000000	0.620817
C	-3.928731	0.000000	1.983951
H	-4.963740	0.000000	2.307948
C	-2.931667	0.000000	2.979308
C	-1.609107	0.000000	2.575213
H	-0.841156	0.000000	3.329323
C	-1.255009	0.000000	1.209668
C	1.255009	-0.000000	1.209668
C	1.609107	-0.000000	2.575213
H	0.841156	-0.000000	3.329323
C	2.931667	-0.000000	2.979308
C	3.928731	-0.000000	1.983951
H	4.963740	-0.000000	2.307948
C	3.670364	-0.000000	0.620817
C	2.302202	-0.000000	0.209135
C	-4.810501	0.000000	-0.404962
C	-6.192291	0.000000	0.255851
H	-6.353279	0.888965	0.876335
H	-6.353279	-0.888965	0.876335
H	-6.961305	0.000000	-0.524461
C	-4.724941	-1.258895	-1.283356
H	-5.535597	-1.261393	-2.022071
H	-4.819578	-2.163304	-0.671034
H	-3.773927	-1.303161	-1.815763
C	-4.724941	1.258895	-1.283356
H	-4.819578	2.163304	-0.671034
H	-5.535597	1.261393	-2.022071
H	-3.773927	1.303161	-1.815763
C	-3.340843	0.000000	4.453213
C	-2.135532	0.000000	5.395803
H	-1.508641	-0.888588	5.257916
H	-2.482235	0.000000	6.434886
H	-1.508641	0.888588	5.257916
C	-4.178865	1.254625	4.748745
H	-3.598287	2.163296	4.551611
H	-4.488357	1.267482	5.800827
H	-5.083972	1.293935	4.133871
C	-4.178865	-1.254625	4.748745
H	-5.083972	-1.293935	4.133871
H	-4.488357	-1.267482	5.800827
H	-3.598287	-2.163296	4.551611
C	3.340843	-0.000000	4.453213
C	4.178865	1.254625	4.748745
H	5.083972	1.293935	4.133871
H	4.488357	1.267482	5.800827
H	3.598287	2.163296	4.551611
C	4.178865	-1.254625	4.748745
H	5.083972	-1.293935	4.133871
H	3.598287	-2.163296	4.551611
H	4.488357	-1.267482	5.800827
C	2.135532	-0.000000	5.395803
H	2.482235	-0.000000	6.434886
H	1.508641	-0.888588	5.257916
H	1.508641	0.888588	5.257916
C	4.810501	-0.000000	-0.404962
C	4.724941	-1.258895	-1.283356
H	5.535597	-1.261393	-2.022071
H	3.773927	-1.303161	-1.815763
H	4.819578	-2.163304	-0.671034
C	4.724941	1.258895	-1.283356
H	5.535597	1.261393	-2.022071
H	4.819578	2.163304	-0.671034
H	3.773927	1.303161	-1.815763
C	6.192291	-0.000000	0.255851
H	6.353279	-0.888965	0.876335
H	6.353279	0.888965	0.876335
H	6.961305	-0.000000	-0.524461
O	1.959665	-0.000000	-1.052046
N	0.000000	0.000000	0.659793
H	0.000000	3.888559	-5.888389
O	-1.959665	0.000000	-1.052046
N	0.000000	1.511361	-2.829388
C	1.149384	1.932254	-3.375051
C	-1.149384	1.932254	-3.375051
C	1.195848	2.784214	-4.470010
C	-1.195848	2.784214	-4.470010
C	0.000000	3.221001	-5.029512
H	2.054815	1.557749	-2.904195
H	-2.054815	1.557749	-2.904195
H	-2.156333	3.094209	-4.873753
H	2.156333	3.094209	-4.873753
N	-0.000000	-1.511361	-2.829388
C	-1.149384	-1.932254	-3.375051
C	1.149384	-1.932254	-3.375051
C	-1.195848	-2.784214	-4.470010
H	-2.054815	-1.557749	-2.904195
C	1.195848	-2.784214	-4.470010
H	2.054815	-1.557749	-2.904195
C	-0.000000	-3.221001	-5.029512
H	-2.156333	-3.094209	-4.873753
H	2.156333	-3.094209	-4.873753
H	-0.000000	-3.888559	-5.888389

Table S33. Optimized Cartesian coordinates for $\mathbf{5^S}$ (S = 0, open-shell singlet state).

Cu	0.114562	1.803842	0.000000
C	-2.378803	0.409837	0.000000
C	-3.789337	0.067455	0.000000
C	-4.138264	-1.260646	0.000000
H	-5.191350	-1.522332	0.000000
C	-3.197704	-2.322048	0.000000
C	-1.857749	-2.008851	0.000000
H	-1.168593	-2.831759	0.000000
C	-1.372603	-0.674012	0.000000
C	1.084013	-0.950886	0.000000
C	1.253377	-2.361996	0.000000
H	0.394663	-3.006141	0.000000
C	2.485609	-2.974812	0.000000
C	3.644492	-2.157324	0.000000
H	4.609660	-2.653677	0.000000
C	3.608069	-0.785352	0.000000
C	2.313549	-0.124641	0.000000
C	-4.845500	1.177844	0.000000
C	-6.271758	0.619853	0.000000
H	-6.476917	0.011932	0.888836
H	-6.476917	0.011932	-0.888836
H	-6.983222	1.453090	0.000000
C	-4.693609	2.049891	-1.259309
H	-5.457182	2.837624	-1.263755
H	-4.827721	1.444686	-2.163934
H	-3.708267	2.518118	-1.295928
C	-4.693609	2.049891	1.259309
H	-4.827721	1.444686	2.163934
H	-5.457182	2.837624	1.263755
H	-3.708267	2.518118	1.295928
C	-3.708245	-3.763170	0.000000
C	-2.574437	-4.789709	0.000000
H	-1.938987	-4.697411	-0.888164
H	-2.994419	-5.801322	0.000000
H	-1.938987	-4.697411	0.888164
C	-4.565122	-3.998445	1.255064
H	-3.971681	-3.841270	2.163241
H	-4.946435	-5.026581	1.268603
H	-5.426232	-3.323380	1.294870
C	-4.565122	-3.998445	-1.255064
H	-5.426232	-3.323380	-1.294870
H	-4.946435	-5.026581	-1.268603
H	-3.971681	-3.841270	-2.163241
C	2.655246	-4.494503	0.000000
C	3.436085	-4.918810	1.255251
H	4.426655	-4.454318	1.298657
H	3.577244	-6.006318	1.266277
H	2.891225	-4.635093	2.163182
C	3.436085	-4.918810	-1.255251
H	4.426655	-4.454318	-1.298657
H	2.891225	-4.635093	-2.163182
H	3.577244	-6.006318	-1.266277
C	1.318777	-5.237483	0.000000
H	1.499320	-6.317872	0.000000
H	0.720889	-5.004029	-0.888424
H	0.720889	-5.004029	0.888424
C	4.889532	0.055315	0.000000
C	4.942338	0.938797	-1.259531
H	5.869378	1.525476	-1.267555
H	4.094663	1.625715	-1.294150
H	4.926299	0.319236	-2.164222
C	4.942338	0.938797	1.259531
H	5.869378	1.525476	1.267555
H	4.926299	0.319236	2.164222
H	4.094663	1.625715	1.294150
C	6.150243	-0.814086	0.000000
H	6.210791	-1.453065	-0.888644
H	6.210791	-1.453065	0.888644
H	7.033384	-0.165570	0.000000
N	-0.079880	-0.235043	0.000000
O	2.238444	1.135316	0.000000
O	-2.009095	1.620053	0.000000
C	1.552154	4.048288	1.290067
C	1.407719	4.841009	0.000000
H	2.459516	3.438582	1.227918
H	1.680535	4.751567	2.131638
N	0.443130	3.124210	1.607717
C	1.552154	4.048288	-1.290067
H	0.489969	5.439881	0.000000
H	2.223099	5.574990	0.000000
C	-0.780218	3.859188	1.951343
C	0.833056	2.288893	2.752740
H	2.459516	3.438582	-1.227918
H	1.680535	4.751567	-2.131638
N	0.443130	3.124210	-1.607717
H	-1.122076	4.454967	1.103842
H	-1.569341	3.144419	2.192300
H	-0.613565	4.527584	2.812041
H	1.723438	1.712799	2.492090
H	0.021479	1.593818	2.984718
H	1.040921	2.903370	3.644743
C	-0.780218	3.859188	-1.951343
C	0.833056	2.288893	-2.752740
H	-1.122076	4.454967	-1.103842
H	-1.569341	3.144419	-2.192300
H	-0.613565	4.527584	-2.812041
H	1.723438	1.712799	-2.492090
H	0.021479	1.593818	-2.984718
H	1.040921	2.903370	-3.644743

Table S34. Optimized Cartesian coordinates for **5^{T}** (S = 1, triplet state).

Cu	-0.091025	-1.577417	0.000000
C	2.321394	-0.305010	0.000000
C	3.721092	-0.019049	0.000000
C	4.103170	1.315227	0.000000
H	5.163475	1.543363	0.000000
C	3.200595	2.397531	0.000000
C	1.845942	2.115347	0.000000
H	1.146736	2.934193	0.000000
C	1.370580	0.787872	0.000000
C	-1.128664	1.007323	0.000000
C	-1.371837	2.396916	0.000000
H	-0.544906	3.086814	0.000000
C	-2.659333	2.905961	0.000000
C	-3.733587	1.994192	0.000000
H	-4.739786	2.399299	0.000000
C	-3.582482	0.614405	0.000000
C	-2.252886	0.096480	0.000000
C	4.757732	-1.148364	0.000000
C	6.195136	-0.620773	0.000000
H	6.413337	-0.017651	0.888841
H	6.413337	-0.017651	-0.888841
H	6.888138	-1.469533	0.000000
C	4.584352	-2.017298	-1.256973
H	5.330260	-2.821732	-1.265194
H	4.723772	-1.416304	-2.163471
H	3.590389	-2.466315	-1.287202
C	4.584352	-2.017298	1.256973
H	4.723772	-1.416304	2.163471
H	5.330260	-2.821732	1.265194
H	3.590389	-2.466315	1.287202
C	3.739819	3.829291	0.000000
C	2.622741	4.875262	0.000000
H	1.985837	4.793095	-0.888322
H	3.059910	5.879662	0.000000
H	1.985837	4.793095	0.888322
C	4.600606	4.049975	1.254479
H	4.006049	3.898941	2.163008
H	4.997378	5.072349	1.270812
H	5.450895	3.361090	1.290832
C	4.600606	4.049975	-1.254479
H	5.450895	3.361090	-1.290832
H	4.997378	5.072349	-1.270812
H	4.006049	3.898941	-2.163008
C	-2.947696	4.408705	0.000000
C	-3.758945	4.773215	1.254217
H	-4.711945	4.235536	1.293265
H	-3.980303	5.847360	1.266989
H	-3.197064	4.528845	2.163208
C	-3.758945	4.773215	-1.254217
H	-4.711945	4.235536	-1.293265
H	-3.197064	4.528845	-2.163208
H	-3.980303	5.847360	-1.266989
C	-1.669863	5.250570	0.000000
H	-1.931236	6.314378	0.000000
H	-1.056143	5.062212	-0.888559
H	-1.056143	5.062212	0.888559
C	-4.791052	-0.328521	0.000000
C	-4.765006	-1.212418	-1.258769
H	-5.629720	-1.887433	-1.264557
H	-3.856199	-1.815526	-1.298212
H	-4.809944	-0.594677	-2.163533
C	-4.765006	-1.212418	1.258769
H	-5.629720	-1.887433	1.264557
H	-4.809944	-0.594677	2.163533
H	-3.856199	-1.815526	1.298212
C	-6.121517	0.429217	0.000000
H	-6.236638	1.060372	-0.888689
H	-6.236638	1.060372	0.888689
H	-6.945557	-0.292892	0.000000
N	0.074121	0.351948	0.000000
O	-2.016911	-1.186793	0.000000
O	1.867457	-1.528242	0.000000
C	-1.379661	-4.066090	1.289495
C	-1.178813	-4.846510	0.000000
H	-2.320330	-3.510339	1.218936
H	-1.481771	-4.777136	2.128339
N	-0.328712	-3.084612	1.624447
C	-1.379661	-4.066090	-1.289495
H	-0.221831	-5.380049	0.000000
H	-1.941059	-5.635581	0.000000
C	0.930658	-3.751894	1.964835
C	-0.771138	-2.287960	2.776098
H	-2.320330	-3.510339	-1.218936
H	-1.481771	-4.777136	-2.128339
N	-0.328712	-3.084612	-1.624447
H	1.327971	-4.291738	1.104067
H	1.666865	-2.999431	2.251809
H	0.793113	-4.460771	2.797644
H	-1.690233	-1.756455	2.519219
H	-0.000603	-1.552867	3.025926
H	-0.953628	-2.922226	3.659485
C	0.930658	-3.751894	-1.964835
C	-0.771138	-2.287960	-2.776098
H	1.327971	-4.291738	-1.104067
H	1.666865	-2.999431	-2.251809
H	0.793113	-4.460771	-2.797644
H	-1.690233	-1.756455	-2.519219
H	-0.000603	-1.552867	-3.025926
H	-0.953628	-2.922226	-3.659485

7. References

(1) Speier, G.; Csihony, J.; Whalen, A. M.; Pierpont, C. G. Studies on Aerobic Reactions of Ammonia/3,5-Di-Tert-Butylcatechol Schiff-Base Condensation Products with Copper, Copper(I), and Copper(II). Strong Copper(II)−Radical Ferromagnetic Exchange and Observations on a Unique N−N Coupling Reaction. *Inorg. Chem.* **1996**, *35* (12), 3519–3524. https://doi.org/10.1021/ic950805l.

(2) Girolami, G. S.; Rauchfuss, T. B.; Angelici, R. J. *Synthesis and Technique in Inorganic Chemistry, 3rd Edition*, 3rd ed.; University Science Books: Sausalito, CA, 1999.

(3) Bain, G. A.; Berry, J. F. Diamagnetic Corrections and Pascal's Constants. *J. Chem. Educ.* **2008**, *85* (4), 532. https://doi.org/10.1021/ed085p532.

(4) Uchida, M.; Cortney, C. H.; Bustos, K.; Manzo, E.; Sauls, E.; Bouchard, J.; Fukazawa, R.; Krishnan, V. V. Discovery-Based Approach to Identify Multiple Factors That Affect the Spin State of Coordination Complexes Using the Evans NMR Method. *J. Chem. Educ.* **2023**, *100* (12), 4822–4827. https://doi.org/10.1021/acs.jchemed.3c00738.

(5) *ADF2019*; SCM, Theoretical Chemistry, Vrije Universiteit, Amsterdam, The Netherlands, 2019.

(6) te Velde, G.; Bickelhaupt, F. M.; Baerends, E. J.; Fonseca Guerra, C.; van Gisbergen, S. J. A.; Snijders, J. G.; Ziegler, T. Chemistry with ADF. *J. Comput. Chem.* **2001**, *22* (9), 931–967. https://doi.org/10.1002/jcc.1056.

(7) Swart, M.; Bickelhaupt, F. M. QUILD: QUantum-Regions Interconnected by Local Descriptions. *J. Comput. Chem.* **2008**, *29* (5), 724–734. https://doi.org/10.1002/jcc.20834.

(8) Van Lenthe, E.; Baerends, E. J. Optimized Slater-Type Basis Sets for the Elements 1-118. *J. Comput. Chem.* **2003**, *24* (9), 1142–1156. https://doi.org/10.1002/jcc.10255.

(9) Chong, D. P.; van Lenthe, E.; Van Gisbergen, S.; Baerends, E. J. Even-Tempered Slater-Type Orbitals Revisited: From Hydrogen to Krypton. *J. Comput. Chem.* **2004**, *25* (8), 1030–1036. https://doi.org/10.1002/jcc.20030.

(10) Wolff, S. K. Analytical Second Derivatives in the Amsterdam Density Functional Package. *Int. J. Quantum Chem.* **2005**, *104* (5), 645–659. https://doi.org/10.1002/qua.20653.

(11) Swart, M. A New Family of Hybrid Density Functionals. *Chem. Phys. Lett.* **2013**, *580*, 166–171. https://doi.org/10.1016/j.cplett.2013.06.045.

(12) Klamt, A.; Schüürmann, G. COSMO: A New Approach to Dielectric Screening in Solvents with Explicit Expressions for the Screening Energy and Its Gradient. *Journal of the Chemical Society, Perkin Transactions 2* **1993**, *0* (5), 799–805. https://doi.org/10.1039/P29930000799.

(13) Swart, M.; Rösler, E.; Bickelhaupt, F. M. Proton Affinities in Water of Maingroup-element Hydrides – Effects of Hydration and Methyl Substitution. *Eur. J. Inorg. Chem.* **2007**, *2007* (23), 3646–3654. https://doi.org/10.1002/ejic.200700228.

(14) Averkiev, B. B.; Truhlar, D. G. Free Energy of Reaction by Density Functional Theory: Oxidative Addition of Ammonia by an Iridium Complex with PCP Pincer Ligands. *Catalysis Science & Technology* **2011**, *1* (8), 1526–1529. https://doi.org/10.1039/C1CY00227A.

(15) Klein, J. E. M. N.; Dereli, B.; Que, L.; Cramer, C. J. Why Metal-Oxos React with Dihydroanthracene and Cyclohexadiene at Comparable Rates, despite Having Different C-H Bond Strengths. A Computational Study. *Chem. Commun. (Camb.)* **2016**, *52* (69), 10509–10512. https://doi.org/10.1039/c6cc05395e.

(16) van Lenthe, E.; Baerends, E. J.; Snijders, J. G. Relativistic Regular Two-Component Hamiltonians. *J. Chem. Phys.* **1993**, *99* (6), 4597–4610. https://doi.org/10.1063/1.466059.

(17) Álvarez-Moreno, M.; de Graaf, C.; López, N.; Maseras, F.; Poblet, J. M.; Bo, C. Managing the Computational Chemistry Big Data Problem: The IoChem-BD Platform. *J. Chem. Inf. Model.* **2015**, *55* (1), 95–103. https://doi.org/10.1021/ci500593j.

(18) Wilkinson, M. D.; Dumontier, M.; Aalbersberg, I. J. J.; Appleton, G.; Axton, M.; Baak, A.; Blomberg, N.; Boiten, J.-W.; da Silva Santos, L. B.; Bourne, P. E.; Bouwman, J.; Brookes, A. J.; Clark, T.; Crosas, M.; Dillo, I.; Dumon, O.; Edmunds, S.; Evelo, C. T.; Finkers, R.; Gonzalez-Beltran, A.; Gray, A. J. G.; Groth, P.; Goble, C.; Grethe, J. S.; Heringa, J.; 't Hoen, P. A. C.; Hooft, R.; Kuhn, T.; Kok, R.; Kok, J.; Lusher, S. J.; Martone, M. E.; Mons, A.; Packer, A. L.; Persson, B.; Rocca-Serra, P.; Roos, M.; van Schaik, R.; Sansone, S.-A.; Schultes, E.; Sengstag, T.; Slater, T.; Strawn, G.; Swertz, M. A.; Thompson, M.; van der Lei, J.; van Mulligen, E.; Velterop, J.; Waagmeester, A.; Wittenburg, P.; Wolstencroft, K.; Zhao, J.; Mons, B. The FAIR Guiding Principles for Scientific Data Management and Stewardship. *Sci. Data* **2016**, *3*, 160018. https://doi.org/10.1038/sdata.2016.18.

(19) Sit, P. H.-L.; Car, R.; Cohen, M. H.; Selloni, A. Simple, Unambiguous Theoretical Approach to Oxidation State Determination via First-Principles Calculations. *Inorg. Chem.* **2011**, *50* (20), 10259–10267. https://doi.org/10.1021/ic2013107.

8. Appendix

8.1 Abbreviations

- NMI (N-methylimidazole)
- py (pyridine)
- tmpda (N,N,N’,N’-tetramethylpropane-1,3-diamine)
